# The human posterior cingulate, retrosplenial, and medial parietal cortex effective connectome, and implications for memory and navigation

**DOI:** 10.1002/hbm.26089

**Published:** 2022-09-30

**Authors:** Edmund T. Rolls, Sylvia Wirth, Gustavo Deco, Chu‐Chung Huang, Jianfeng Feng

**Affiliations:** ^1^ Oxford Centre for Computational Neuroscience Oxford UK; ^2^ Department of Computer Science University of Warwick Coventry UK; ^3^ Institute of Science and Technology for Brain Inspired Intelligence Fudan University Shanghai China; ^4^ Key Laboratory of Computational Neuroscience and Brain Inspired Intelligence Fudan University, Ministry of Education Shanghai China; ^5^ Fudan ISTBI—ZJNU Algorithm Centre for Brain‐Inspired Intelligence Zhejiang Normal University Jinhua China; ^6^ Institut des Sciences Cognitives Marc Jeannerod, UMR 5229 CNRS and University of Lyon Bron France; ^7^ Center for Brain and Cognition, Computational Neuroscience Group, Department of Information and Communication Technologies Universitat Pompeu Fabra Barcelona Spain; ^8^ Brain and Cognition Pompeu Fabra University Barcelona Spain; ^9^ Institució Catalana de la Recerca i Estudis Avançats (ICREA) Universitat Pompeu Fabra Barcelona Spain; ^10^ Shanghai Key Laboratory of Brain Functional Genomics (Ministry of Education), School of Psychology and Cognitive Science East China Normal University Shanghai China

**Keywords:** hippocampus, memory, midcingulate cortex, navigation, posterior cingulate cortex, retrosplenial cortex, spatial view cells, visuo‐motor coordinate transforms

## Abstract

The human posterior cingulate, retrosplenial, and medial parietal cortex are involved in memory and navigation. The functional anatomy underlying these cognitive functions was investigated by measuring the effective connectivity of these Posterior Cingulate Division (PCD) regions in the Human Connectome Project‐MMP1 atlas in 171 HCP participants, and complemented with functional connectivity and diffusion tractography. First, the postero‐ventral parts of the PCD (31pd, 31pv, 7m, d23ab, and v23ab) have effective connectivity with the temporal pole, inferior temporal visual cortex, cortex in the superior temporal sulcus implicated in auditory and semantic processing, with the reward‐related vmPFC and pregenual anterior cingulate cortex, with the inferior parietal cortex, and with the hippocampal system. This connectivity implicates it in hippocampal episodic memory, providing routes for “what,” reward and semantic schema‐related information to access the hippocampus. Second, the antero‐dorsal parts of the PCD (especially 31a and 23d, PCV, and also RSC) have connectivity with early visual cortical areas including those that represent spatial scenes, with the superior parietal cortex, with the pregenual anterior cingulate cortex, and with the hippocampal system. This connectivity implicates it in the “where” component for hippocampal episodic memory and for spatial navigation. The dorsal–transitional–visual (DVT) and ProStriate regions where the retrosplenial scene area is located have connectivity from early visual cortical areas to the parahippocampal scene area, providing a ventromedial route for spatial scene information to reach the hippocampus. These connectivities provide important routes for “what,” reward, and “where” scene‐related information for human hippocampal episodic memory and navigation. The midcingulate cortex provides a route from the anterior dorsal parts of the PCD and the supracallosal part of the anterior cingulate cortex to premotor regions.

## Graphical abstract

First, the postero‐ventral parts of the Posterior Cingulate Division of the Human Connectome Project Multimodal Parcellation atlas (PCD; 31pd, 31pv, 7m, d23ab, and v23ab) have effective connectivity with the temporal pole, inferior temporal visual cortex, cortex in the superior temporal sulcus implicated in auditory and semantic processing, with the reward‐related vmPFC and pregenual anterior cingulate cortex, with the inferior parietal cortex, and with the hippocampal system; and this connectivity implicates these regions in hippocampal episodic memory, providing routes for “what,” reward, and semantic schema‐related information to access the hippocampus. Second, the antero‐dorsal parts of the PCD (especially 31a and 23d, PCV, and also RSC) have connectivity with early visual cortical areas including those that represent spatial scenes, with the superior parietal cortex, with the pregenual anterior cingulate cortex, and with the hippocampal system. This connectivity implicates them in the “where” component for hippocampal episodic memory and for spatial navigation. Third, the dorsal–transitional–visual (DVT) and ProStriate regions are where the retrosplenial scene area is located, have connectivity from early cortical visual areas, connect to the parahippocampal scene area, and provide a ventromedial cortex route for scene information to reach the hippocampal system.

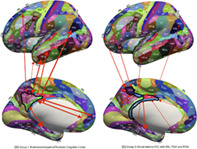

## 1 INTRODUCTION

**FIGURE 1:**
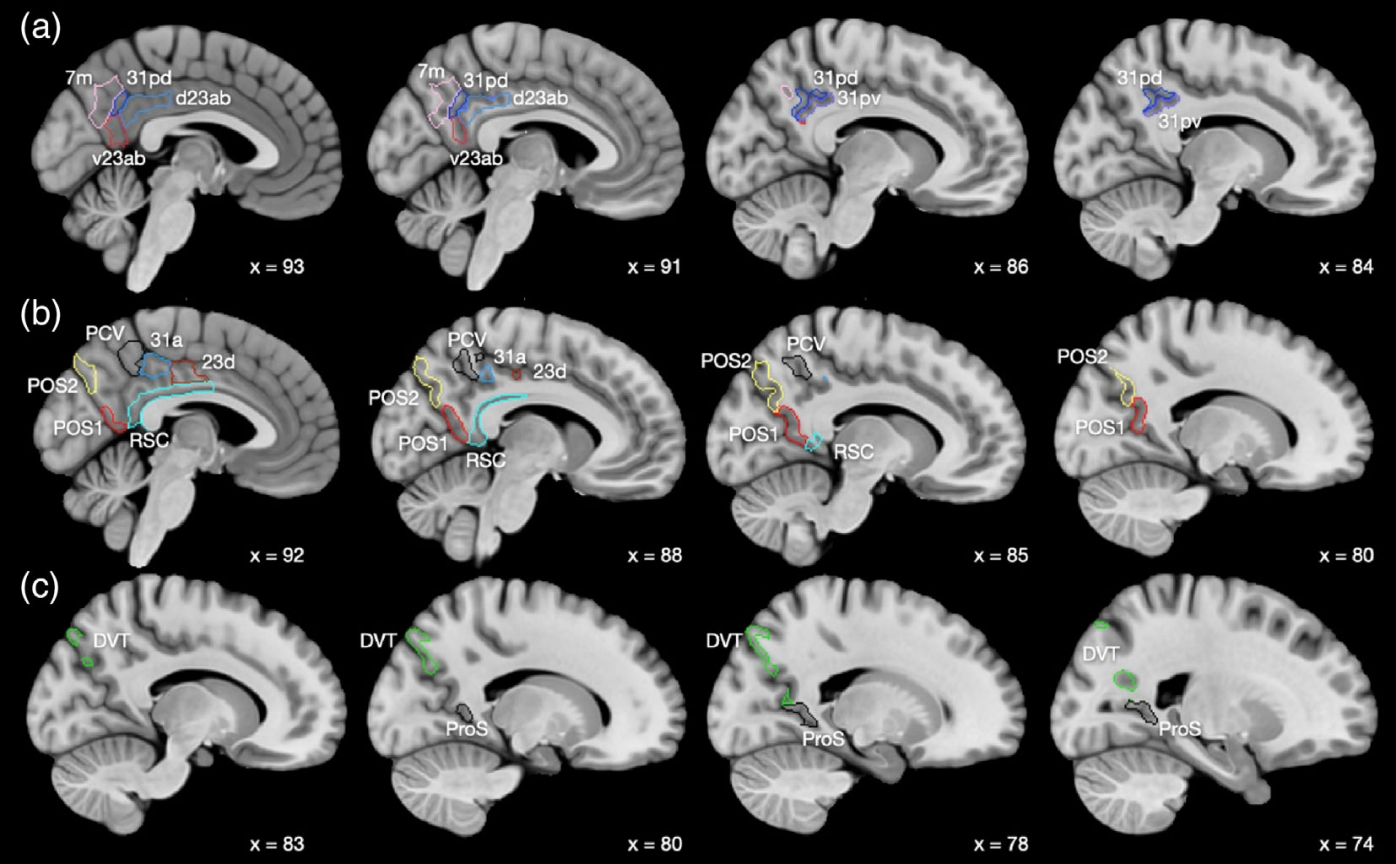
Anatomical regions of the human posterior cingulate cortex division as defined in the HCP‐MMP atlas (Glasser, Coalson, et al., 2016a), and in its extended version HCPex (Huang et al., 2022), both used here. Abbreviations are provided in Table S1. Sagittal slices are shown, with *x* = 93 at the midline, and *x* = 74 lateral. (a) The regions in Group 1 are 31pd, 31pv, 7m, d23ab, and v23ab. (b) The regions in Group 2 are RSC (retrosplenial cortex), 31a, 23d, PCV (precuneus visual region), POS2, and POS1 (regions in the parieto‐occipital sulcus). (c) The regions in Group 3 are DVT region and ProS region. The groups are based on the effective and functional connectivity with other cortical areas and on the anatomical locations of each region, as described in Section 2. The MCC (regions 23c, 24dd, and 24dv) are anterior to 23d, and are not included here for clarity.

The human posterior cingulate cortex (PCC) includes Brodmann areas 23 and 31, is also present in macaques, and is absent in rodents (Vogt, 2009). The retrosplenial cortex (RSC) Brodmann area 29/30 in humans is a small region wrapped round the splenium of the corpus callosum (Vogt, 2009, 2019a; Vogt et al., 1995; see Figures 1 and 8). The PCC has been divided into a dorsal part with connections with prefrontal and parietal areas; and a ventral part more posteriorly with connections with medial temporal lobe regions (Vogt, 2009, 2019a). The posterior cingulate cortex is part of the default mode network, which shows deactivation when humans perform tasks with external stimuli (Buckner & DiNicola, 2019). That suggests that the PCC/RSC may be more involved in internal for example memory‐related processing, and indeed tasks that require episodic memory retrieval, autobiographical remembering, and theory of mind activate the PCC (Buckner & DiNicola, 2019); and damage to the human PCC/RSC can impair episodic memory and perhaps attention (Leech & Sharp, 2014; Leech & Smallwood, 2019; Vann et al., 2009). The ventral part of the PCC is especially involved in these memory‐related functions, whereas the dorsal part of the PCC is activated during some executive function tasks such as visual search and mental arithmetic (Buckner & DiNicola, 2019; Chrastil et al., 2018; Dastjerdi et al., 2011; Foster et al., 2012, 2013; Fox et al., 2018). The ventral part may have one subpart linked to the parahippocampal cortex that is preferentially activated during episodic remembering and imagining the future; and a second subpart which includes the temporoparietal junction is activated during theory of mind (DiNicola et al., 2020). A meta‐analysis suggested that ventral portions of the posterior cingulate cortex were more likely to be activated by spatial encoding, that is, passive viewing of scenes, whereas dorsal portions of the posterior cingulate cortex were more likely to be activated by cognitive demands to recall spatial information or to produce judgments of distance or direction to nonvisible locations or landmarks (Burles et al., 2018). Part of the ventral PCC has activations related to value, with responses in macaques related, for example, to risky decisions (McCoy & Platt, 2005; Pearson et al., 2011). Indeed, the PCC has effective connectivity in humans with reward‐related brain regions including the ventromedial prefrontal cortex and pregenual anterior cingulate cortex (Rolls et al., 2022e). The human PCC/RSC is also implicated in navigation (Ekstrom et al., 2017; Teghil et al., 2021).

Given this heterogeneity of functions of different parts of the PCC/RSC, and the importance of understanding brain computations of evidence about the connectivity of different brain regions (Rolls, 2021a), the present investigation was to advance the understanding of the connections and connectivity of the human PCC/RSC and its closely related regions in the medial parietal cortex and cortex in the parieto‐occipital sulcus as defined in the Human Connectome Project Multimodal Parcellation atlas (HCP‐MMP) in what is termed the Posterior Cingulate Cortical Division in that atlas (Baker et al., 2018; Glasser, Coalson, et al., 2016a; see Table S1), and thereby to help elucidate the functioning of its different parts. All the cortical regions in the Posterior Cingulate Cortical Division of the HCP‐MMP were included in the analysis described here, partly because they have interconnectivity and related functions, and partly so that we can characterize the connectivity of all 360 cortical regions in the HCP‐MMP atlas by systematically working through each division in the HCP‐MMP atlas (Rolls et al., 2022a, 2022b, 2022c, 2022d, 2022e, 2022f), with the different divisions and the cortical regions within them shown in Table S1. To perform this research on the cortical regions in the Posterior Cingulate Cortical Division of the HCP‐MMP, we measured with Human Connectome Project data (Glasser, Smith, et al., 2016b) (i) the direct connections between brain regions using diffusion tractography (Huang et al., 2021); (ii) the functional connectivity between brain regions using the correlation between the BOLD signals in resting‐state fMRI which provides evidence about the strength of interactions; and (iii) the effective connectivity which provides evidence about the strength and direction of the causal connectivity between pairs of hundreds of brain regions with the new Hopf algorithm that enables measurement of effective connectivity in both directions between every pair of the brain regions (Rolls et al., 2022e; Rolls et al., 2022f). These measures were made between the 360 cortical regions in the Human Connectome Project multimodal parcellation atlas (HCP‐MMP; Glasser, Coalson, et al., 2016a), to which we added 66 subcortical areas in the extended HCP atlas (HCPex; Huang et al., 2022). The HCP‐MMP atlas provides the most detailed parcellation of the human cortical areas that we know, in that its 360 regions are defined using a “multimodal” combination of structural measures (cortical thickness and cortical myelin content), functional connectivity, and task‐related fMRI (Glasser, Coalson, et al., 2016a). This parcellation is the parcellation of choice for the cerebral cortex because it is based on multimodal information (Glasser, Coalson, et al., 2016a) with the definition and boundaries set out in their Glasser_2016_SuppNeuroanatomy.pdf, and it is being used as the basis for many new investigations of brain function and connectivity, which can all be cast in the same framework (Colclough et al., 2017; Rolls et al., 2022a, 2022b, 2022e, 2022f; Sulpizio et al., 2020; van Essen & Glasser, 2018; Yokoyama et al., 2021). This approach provides better categorization of cortical areas than does, for example, functional connectivity alone (Power et al., 2011).

Because we used the HCP‐MMP1 atlas in this research, we included in the brain regions investigated in this article all the regions included in the “Posterior Cingulate Division” of the human cerebral cortex in the HCP‐MMP1 atlas, which are listed in Table S1 and defined as in this Division in the HCP‐MMP1 atlas (Glasser, Coalson, et al., 2016a). This strategy facilitates systematic studies in which the connectivity of different parts of the cerebral cortex is considered brain system by brain system which is the approach being taken (Rolls et al., 2022a, 2022b, 2022e, 2022f), and minimizes the chance that individual brain regions will be omitted if the whole posterior cingulate division was not included here. Of the brain areas included in the posterior cingulate cortex division of the HCP‐MMP, 31pd, 31pv, d23ab, v23ab, 23d, and 31a are the usual parts that would be classified as posterior cingulate cortex (Vogt, 2009). The RSC (retrosplenial cortex) region in the HCP‐MMP1 atlas (Glasser, Coalson, et al., 2016a) does have a thin part that extends anteriorly (Figures 1, 8, and S1). The precuneus visual area (PCV) with 7m are regions of medial parietal cortex that correspond to the precuneus (Baker et al., 2018), which is implicated in visual and self‐referential processing (Cavanna & Trimble, 2006; Freton et al., 2014) probably partly because of connectivity with the cortex in the superior temporal sulcus which is implicated in processing socially relevant stimuli related to face expression and gesture (Hasselmo, Rolls, & Baylis, 1989a; Hasselmo, Rolls, Baylis, & Nalwa, 1989b; Lee Masson & Isik, 2021). POS2 and POS1 are parieto‐occipital sulcus visual areas (Figures 1, 8, and S1). The ProStriate region (ProS) is adjacent to V1, and the dorsal transitional visual area (DVT) is an area posterior to most of the PCC, found just lateral to POS2 (Figures 1, 8, S1, and S1‐4; Glasser, Coalson, et al., 2016a). Further, as shown here, many of these regions not parts of posterior cingulate cortex areas 23 and 31 have strong connectivity with parts of areas 23 and 31.

In addition to the PCC/RSC/medial parietal regions as specified in the HCP‐MMP atlas Posterior Cingulate Division (see Section 2 and Figures 1 and 8), the midcingulate cortical (MCC) areas were included in the analysis, as it is useful to understand whether this premotor part of the cingulate cortex (Vogt, 2016) has markedly different connectivity from all the PCC/RSC regions, and further whether any PCC/RSC/medial parietal regions have connectivity directed especially to the MCC. Much of the previous thinking about the connectivity of the PCC/RSC in humans was based on investigations in macaques, and the macaque evidence is summarized in the Section 4 for comparison with what is described here about the connectivity of the PCC/RSC/medial parietal regions in humans.

We know of no previous research on effective connectivity of the human posterior cingulate division of the HCP‐MMP atlas. Previous research on the connections of the posterior cingulate cortex and related regions including the MCC in macaques with reference to humans has been provided elsewhere (van Heukelum et al., 2020; Vogt, 2009, 2016, 2019b; Vogt & Laureys, 2009) and further studies are described in Section 4. A meta‐analysis showed that anterior retrosplenial regions had functional connectivity with the default mode network and were associated with episodic memory, whereas posterior retrosplenial regions in the parietal–occipital sulcus had functional connectivity with visual regions and were associated with scenes and navigation (Chrastil et al., 2018). An investigation with diffusion tractography and fMRI in humans of the retrosplenial cortex showed that BA29 has fiber connections with auditory cortex and functional connections with BA21 (inferior temporal visual cortex), and that BA30 has fiber connections with the visual cortex, hippocampus, and prefrontal cortex (Li et al., 2018).

Strengths of this investigation are that it utilized the multimodal HCP‐MMP1 atlas (Glasser, Coalson, et al., 2016a); HCP data from the same set of 171 participants imaged at 7T (Glasser, Smith, et al., 2016b) in whom we could calculate the connections, functional connectivity, and effective connectivity; and that it utilized a method for effective connectivity measurement between all 360 cortical regions investigated here and 66 subcortical regions defined in the HCPex version (Huang et al., 2022) of the HCP‐MMP1 atlas (Glasser, Coalson, et al., 2016a). Section 4 considers the implications for function of the new evidence on the connectome of the posterior cingulate and medial parietal regions described here, and that discussion is facilitated by the fact that some activation studies now refer to region names used in the HCP‐MMP1 atlas, allowing function to be linked to brain connectivity, which is a key aim of the approach to brain structure and function considered here and elsewhere (Rolls, 2021a).

## 2 METHODS

### 2.1 Participants and data acquisition

Multiband 7T resting‐state functional magnetic resonance images (rs‐fMRI) of 184 individuals were obtained from the publicly available S1200 release (last updated: April 2018) of the Human Connectome Project (HCP; van Essen et al., 2013). Individual written informed content was obtained from each participant, and the scanning protocol was approved by the Institutional Review Board of Washington University in St. Louis, Missouri (IRB #201204036).

Multimodal imaging was performed in a Siemens Magnetom 7T housed at the Center for Magnetic Resonance (CMRR) at the University of Minnesota in Minneapolis. For each participant, a total of four sessions of rs‐fMRI were acquired with the eyes open and fixating, with oblique axial acquisitions alternated between phase encoding in a posterior‐to‐anterior (PA) direction in sessions 1 and 3, and an anterior‐to‐posterior (AP) phase encoding direction in sessions 2 and 4. Specifically, each rs‐fMRI session was acquired using a multiband gradient‐echo EPI imaging sequence. The following parameters were used: TR = 1000 ms, TE = 22.2 ms, flip angle = 45°, field of view = 208 × 208, matrix = 130 × 130, 85 slices, voxel size = 1.6 × 1.6 × 1.6 mm^3^, and multiband factor = 5. The total scanning time for the rs‐fMRI protocol was ~16 min with 900 volumes. Further details of the 7T rs‐fMRI acquisition protocols are given in the HCP reference manual (https://humanconnectome.org/storage/app/media/documentation/s1200/HCP_S1200_Release_Reference_Manual.pdf).

The current investigation was designed to complement an investigation of effective connectivity of the hippocampus (Rolls et al., 2022f), and so the same 171 participants were used for the analyses described here (age 22–36 years, 66 males), each with four sessions of 7T rs‐fMRI.

### 2.2 Data preprocessing

The preprocessing was performed by the HCP as described in Glasser, et al. (2013), based on the updated 7T data pipeline (v3.21.0, https://github.com/Washington-University/HCPpipelines), including gradient distortion correction, head motion correction, image distortion correction, spatial transformation to the Montreal Neurological Institute space using one step spline resampling from the original functional images followed by intensity normalization. In addition, the HCP took an approach using ICA (FSL's MELODIC) combined with a more automated component classifier referred to as FIX (FMRIB's ICA‐based X‐noisifier) to remove non‐neural spatiotemporal artifact (Griffanti et al., 2014; Salimi‐Khorshidi et al., 2014; Smith, et al., 2013). This step also used 24 confound timeseries derived from the motion estimation (6 rigid‐body parameter timeseries, their backwards‐looking temporal derivatives, plus all 12 resulting regressors squared; Satterthwaite et al., 2013) to minimize noise in the data.

### 2.3 Brain atlas and seed selection

To construct the effective connectivity for the cortical regions of interest in this investigation with other cortical regions, we utilized the surface‐based HCP‐MMP1 atlas which defines 360 cortical regions (Glasser, Coalson, et al., 2016a). We were able to use the same 171 participants for whom we also had performed diffusion tractography, as described in detail (Huang et al., 2021). The brain regions in this atlas (Glasser, Coalson, et al., 2016a) are shown in Figures 1 and S1, and a list of the cortical regions in this atlas is provided in Table S1 in the reordered form used in the extended volumetric HCPex atlas (Huang et al., 2022). The timeseries for the four sessions for each participant were extracted for each region in the surface‐based atlas using the HCP protocol and software (Glasser, Coalson, et al., 2016a), and the functional and effective connectivity were measured using all four timeseries for each participant as described below. The functional connectivity and lagged functional connectivity were calculated separately for each of the four timeseries, and then the average of each set of four functional connectivities was taken. Compared with a previous study (Rolls et al., 2022f), 1 participant was excluded leaving 171 participants because all four timeseries were not of sufficient length.

For the subcortical regions, the HCPex atlas (Huang et al., 2022) was used, for with its volumetric approach it defines in addition to 180 cortical regions per hemisphere, 33 subcortical regions including the amygdala, thalamus, putamen, caudate nucleus, nucleus accumbens, globus pallidus, mammillary bodies, septal nuclei, and nucleus basalis. For the subcortical analyses, we were able to define as an extra cortical region the subiculum, as described elsewhere (Huang et al., 2022; Rolls et al., 2022e, 2022f).

Lists of the regions in these atlases (Glasser, Coalson, et al., 2016a; Huang et al., 2022) are provided in Tables S1 and S2, and coronal slices and views of the brain with the HCP parcellation with labels for each region are provided in Figure S1 (Huang et al., 2022).

In this investigation, the regions of interest (ROIs) included the following from the HCP‐MMP1 atlas, and they were grouped into three groups based partly on the similarities and differences in the connectivity of these brain regions, as shown in the correlation matrices between the effective and functional connectivities of these brain regions with all other cortical regions presented in Figures S4 and S5, but also on the anatomical locations of these brain regions. It is emphasized that the groups were purely to help the presentation of the results by describing regions with some similarity into groups, and that no analyses depended on these groups. In more detail, to explain the rationale for the groups, the Group 1 regions consisted of 31pd, 31pv, 7m, d23ab, and v23ab, and were placed together in this group because their effective and functional connectivities with other cortical regions were relatively similar to each other as illustrated in Figures S4 and S5. In further detail, the effective and functional connectivities of these regions were more highly correlated with each other than with other cortical regions (Figures S4 and S5). The similarity of the effective connectivity of these Group 1 regions was confirmed by a community analysis using the Brain Connectivity Toolbox (Rubinov & Sporns, 2010; https://www.nitrc.org/projects/bct) which placed these five cortical regions into the same community.

The DVT area and ProS area are visual cortical regions in the HCP‐MMP1 that are transitional between earlier visual cortical regions and different more anterior types of cortex (Glasser, Coalson, et al., 2016a), and because they are described as transitional in the HCP‐MMP (having architectural properties similar to their anterior neighbors and functional and connectional patterns more similar to their posterior neighbors [Glasser, Coalson, et al., 2016a]) they were placed together in their own Group 3.

The rationale was then that the remaining regions in the Posterior Cingulate Division of the HCP‐MMP1 (Glasser, Coalson, et al., 2016a) that were not placed in Groups 1 and 3 as just described made up Group 2 which consists of RSC, 31a, 23d, PCV, POS2, and POS1 (see Figure 1 in which a–c correspond to Groups 1–3; and also Figure 8).

In addition, to compare the connectivity of the posterior cingulate cortex with that of the MCC (the cingulate motor area), a fourth group is shown in most analyses, and consisted of 23c, 24dd, and 24dv which are MCC regions.

It is again emphasized that in practice, each cortical region was analyzed separately, and no analyses presented in the article depend on this grouping, which is for ease of description.

It is noted that an alternative anatomical terminology to the multimodal HCP‐MMP1 (Glasser, Coalson, et al., 2016a) for some parts of the cingulate cortex has been described (Rolls, 2019a, 2019b; Vogt, 2009, 2016, 2019b).

### 2.4 Measurement of effective connectivity

Effective connectivity measures the effect of one brain region on another, and utilizes differences detected at different times in the signals in each connected pair of brain regions to infer the effects of one brain region on another. One such approach is dynamic causal modeling, but it applies most easily to activation studies, and is typically limited to measuring the effective connectivity between just a few brain areas (Bajaj et al., 2016; Friston, 2009; Valdes‐Sosa et al., 2011), although there have been moves to extend it to resting state studies and more brain areas (Frassle et al., 2017; Razi et al., 2017). The method used here (see Rolls et al., 2022f) was developed from a Hopf algorithm to enable measurement of effective connectivity between many brain areas, described by Deco et al. (2019). A principle is that the functional connectivity is measured at time *t* and time *t + tau*, where *tau* is typically 2 s to take into account that this is the time within which a change in the BOLD signal can occur, and that *tau* should be short to capture causality, and then the effective connectivity model is trained by error correction until it can generate the functional connectivity matrices at time *t* and time *t + tau*. The algorithm, and the development that enabled it to measure the effective connectivity in each direction, are described briefly next and in more detail in the Appendix S1, and including validation elsewhere (Rolls et al., 2022a, 2022b, 2022d, 2022e, 2022f).

To infer effective connectivity, we use a whole‐brain model that allows us to simulate the BOLD activity across all brain regions and time. We use the so‐called Hopf computational model, which integrates the dynamics of Stuart–Landau oscillators, expressing the activity of each brain region, by the underlying anatomical connectivity (Deco, Kringelbach, et al., 2017b). As mentioned above, we include in the model 360 cortical brain areas (Huang et al., 2022). The local dynamics of each brain area (node) is given by Stuart–Landau oscillators which expresses the normal form of a supercritical Hopf bifurcation, describing the transition from noisy to oscillatory dynamics (Kuznetsov, 2013). During the last years, numerous studies were able to show how the Hopf whole‐brain model successfully simulates empirical electrophysiology (Freyer et al., 2011, 2012), MEG (Deco, Cabral, et al., 2017a) and fMRI (Deco, Kringelbach, et al., 2017b; Kringelbach et al., 2015; Kringelbach & Deco, 2020).

The Hopf whole‐brain model can be expressed mathematically as follows:

(1) $$\frac{dx_{i}}{\mathrm{dt}}=\overset{\text{Local Dynamics}}{\overbrace{\left[a_{i}-x_{i}^{2}-y_{i}^{2}\right]x_{i}-\omega_{i}y_{i}}}+\overset{\text{Coupling}\;}{\;\overbrace{G\sum_{j=1}^{N}C_{\mathrm{ij}}\left(x_{j}-x_{i}\right)}}+\overset{\text{Gaussian Noise}\;}{\overbrace{\beta \eta_{i}\left(t\right)}}$$

(2) $$\frac{dy_{i}}{\mathrm{dt}}=\left[a_{i}-x_{i}^{2}-y_{i}^{2}\right]y_{i}+\omega_{i}x_{i}+G\sum_{j=1}^{N}C_{\mathrm{ij}}\left(y_{j}-y_{i}\right)+\beta \eta_{i}\left(t\right)$$

Equations (1) and (2) describe the coupling of Stuart–Landau oscillators through an effective connectivity matrix *C*. The $x_{i}\left(t\right)$ term represents the simulated BOLD signal data of brain area *i*. The values of $y_{i}\left(t\right)$ are relevant to the dynamics of the system but are not part of the information read out from the system. In these equations, $\eta_{i}\left(t\right)$ provides additive Gaussian noise with standard deviation *β*. The Stuart–Landau oscillators for each brain area *i* express a Hopf normal form that has a supercritical bifurcation at $a_{i}=0$, so that if $a_{i}>0$ the system has a stable limit cycle with frequency $f_{i}=\omega_{i}/2\pi$ (where $\omega_{i}$ is the angular velocity); and when $a_{i}<0$ the system has a stable fixed point representing a low activity noisy state. The intrinsic frequency $f_{i}$ of each Stuart–Landau oscillator corresponding to a brain area is in the 0.008–0.08 Hz band (*i* = 1, …, 360). The intrinsic frequencies are fitted from the data, as given by the averaged peak frequency of the narrowband BOLD signals of each brain region. Each brain region has a distinct peak in the power spectrum at a particular frequency, there are some differences in the frequency for different brain regions, and these differences contribute to the accuracy of the effective connectivity estimation (cf. Ponce‐Alvarez et al., 2015). The coupling term representing the input received in node *i* from every other node *j*, is weighted by the corresponding effective connectivity $C_{\mathrm{ij}}$. The coupling is the canonical diffusive coupling, which approximates the simplest (linear) part of a general coupling function. *G* denotes the global coupling weight, scaling equally the total input received in each brain area. While the oscillators are weakly coupled, the periodic orbit of the uncoupled oscillators is preserved. Details are provided in the Appendix S1.

The effective connectivity matrix is derived by optimizing the conductivity of each existing anatomical connection as specified by the Structural Connectivity matrix (measured with tractography; Huang et al., 2021) to fit the empirical functional connectivity (FC) pairs and the lagged FC^tau^ pairs. By this, we are able to infer a non‐symmetric Effective Connectivity matrix (see Gilson et al. [2016]). Note that FC^tau^, that is, the lagged functional connectivity between pairs, lagged at *tau* s, breaks the symmetry and thus is fundamental for our purpose. Specifically, we compute the distance between the model FC simulated from the current estimate of the effective connectivity and the empirical data FC^emp^, as well as the simulated model FC^tau^ and empirical data FC^tau_emp^ and adjust each effective connection (entry in the effective connectivity matrix) separately with a gradient‐descent approach. The model is run repeatedly with the updated effective connectivity until the fit converges toward a stable value.

We start with the anatomical connectivity obtained with probabilistic tractography from dMRI (or from an initial zero *C* matrix as described in the Appendix S1) and use the following procedure to update each entry $C_{\mathrm{ij}}\;$ in the effective connectivity matrix

(3) $$C_{\mathrm{ij}}=C_{\mathrm{ij}}+\varepsilon \left(FC_{\mathrm{ij}}^{\mathrm{emp}}-FC_{\mathrm{ij}}+FC_{\mathrm{ij}}^{\mathrm{tau}\_\mathrm{emp}}-FC_{\mathrm{ij}}^{\mathrm{tau}}\right)$$

where ϵ is a learning rate constant, and *i* and *j* are the nodes. When updating each connection if the initial matrix is a dMRI structural connection matrix (see Appendix S1), the corresponding link to the same brain regions in the opposite hemisphere is also updated, as contralateral connections are not revealed well by dMRI. The convergence of the algorithm is illustrated by Rolls et al. (2022f), and the utility of the algorithm was validated as described below and elsewhere (Rolls et al., 2022a, 2022b, 2022d, 2022e, 2022f).

For the implementation, we set *tau* to be 2 s, selecting the appropriate number of TRs to achieve this. The maximum effective connectivity was set to a value of 0.2, and was found between V1 and V2.

The effective connectivity measurement uses a nonlinear algorithm and performs error correction to obtain optimal estimation. In this process, some links do not contribute to the optimal estimation, and are set to zero. In this situation, and given the reproducibility of the convergence of the algorithm as documented here, even effective connectivity links with low values are very likely to contribute to the optimal estimation of the effective connectivity. It is noted that the effective and functional connectivity were measured in the resting state, and so provide a baseline reference that may change when particular tasks are being performed.

### 2.5 Effective connectome

Whole‐brain effective connectivity (EC) analysis was performed between the 16 regions of interest described above shown in Figures 1 and S1 and the 360 regions defined in the surface‐based HCP‐MMP1 atlas (Glasser, Coalson, et al., 2016a) shown in Table S1 (Huang et al., 2022). This EC was computed for all 171 participants. The effective connectivity algorithm was run until it had reached the maximal value for the correspondence between the simulated and empirical functional connectivity matrices at time *t* and *t + tau* (see Appendix S1).

The effective connectivity calculated between the 360 cortical areas was checked and validated in several ways. First, in all cases, the 360 × 360 effective connectivity matrix could be used to generate by simulation 360 × 360 functional connectivity matrices for time *t* and time *t + tau* that were correlated 0.8 or more with the empirically measured functional connectivity matrices at time *t* and time *t + tau* using fMRI. Second, the effective connectivity matrices were robust with respect to the number of participants, in that when the 171 participants were separated into two groups of 86 and 85, the correlation between the effective connectivities measured for each group independently was 0.98. Third, the effective connectivities for early visual areas V1, V2, V3, and V4 were compared with the known connections for forward and backward connections involving these areas in macaques (Markov et al., 2014), and the human effective connectivity was consistent with the connections in this hierarchically organized system in macaques, with these results shown in Rolls et al. (2022d, 2022f). Fourth, the effective connectivity with in particular the corresponding brain region contralaterally was high relative to other contralateral connectivities, providing clear evidence that the effective connectivity algorithm could identify distant brain regions that could be expected to have high effective connectivity (Rolls et al., 2022d).

To test whether the vectors of effective connectivities of each of the 13 posterior cingulate division cortical regions with the 180 areas in the left hemisphere of the modified HCP atlas were significantly different, the interaction term was calculated for each pair of the 13 posterior cingulate division ROI effective connectivity vectors in separate two‐way ANOVAs (each 2 × 180) across the 171 participants, and Bonferroni correction for multiple comparisons was applied. The results were checked with the nonparametric Scheirer–Rey–Hare test (Scheirer et al., 1976; Sinha, 2022).

### 2.6 Functional connectivity

For comparison with the effective connectivity, the functional connectivity was also measured at 7T with the identical set of participants, data, and filtering of 0.008–0.08 Hz. The functional connectivity was measured by the Pearson correlation between the BOLD signal timeseries for each pair of brain regions, and is in fact the FC^emp^ referred to above. A threshold of 0.4 is used for the presentation of the findings in Figure 5, for this sets the sparseness of what is shown to a level commensurate with the effective connectivity, to facilitate comparison between the functional and the effective connectivity. The functional connectivity can provide evidence that may relate to interactions between brain regions, while providing no evidence about causal direction‐specific effects. A high functional connectivity may in this scenario thus reflect strong physiological interactions between areas, and provides a different type of evidence to effective connectivity. The effective connectivity is nonlinearly related to the functional connectivity, with effective connectivities being identified (i.e., >0) only for the links with relatively high functional connectivity.

### 2.7 Connections shown with diffusion tractography

Diffusion tractography can provide evidence about fiber pathways linking different brain regions with a method that is completely different to the ways in which effective and functional connectivity are measured, so is included here to provide complementary and supporting evidence to the effective connectivity. Diffusion tractography shows only direct connections, so comparison with effective connectivity can help to suggest which effective connectivities may be mediated directly or trans‐synaptically. Diffusion tractography does not provide evidence about the direction of connections. Diffusion tractography was performed on the same 171 HCP participants' images at 7T with methods described in detail elsewhere (Huang et al., 2021). The major parameters were: 1.05 mm isotropic voxels; a two shell acquisition scheme with *b*‐values = 1000, 2000 s/mm^2^, repetition time/echo time = 7000/71 ms, 65 unique diffusion gradient directions and 6 b0 images obtained for each phase encoding direction pair (AP and PA pairs). Preprocessing steps included distortion correction, eddy‐current correction, motion correction, and gradient nonlinearity correction. In brief, whole‐brain tractography was reconstructed for each subject in native space. To improve the tractography termination accuracy in GM, MRtrix3's 5ttgen command was used to generate multitissue segment images (5tt) using T1 images, the segmented tissues were then co‐registered with the b0 image in diffusion space. For multishell data, tissue response functions in GM, WM, and CSF were estimated by the MRtrix3' dwi2response function with the Dhollander algorithm (Dhollander et al., 2016). A Multi‐Shell Multi‐Tissue Constrained Spherical Deconvolution (MSMT‐CSD) model with *l*_max_ = 8 and prior co‐registered 5tt image was used on the preprocessed multishell DWI data to obtain the fiber orientation distribution (FOD) function (Jeurissen et al., 2014; Smith, 2002). Based on the voxel‐wise fiber orientation distribution, anatomically‐constrained tractography (ACT) using the probabilistic tracking algorithm: iFOD2 (2nd order integration based on FOD) with dynamic seeding was applied to generate the initial tractogram (1 million streamlines with maximum tract length = 250 mm and minimal tract length = 5 mm). To quantify the number of streamlines connecting pairs of regions, the updated version of the spherical‐deconvolution informed filtering of the tractograms (SIFT2) method was applied. The use of the SIFT2 algorithm helps to provide a streamline number for the estimates we provide in the figures, that is, at least proportional to the number of connections between each pair of brain regions (Smith et al., 2015).

The results for the tractography are shown in Figure 6 as the number of streamlines between areas with a threshold applied of 10 to reduce the risk of occasional noise‐related observations. The term “connections” is used when referring to what is shown with diffusion tractography, and connectivity when referring to effective or functional connectivity. The terms “projects to” and “projects from” refer to direction and therefore to effective connectivity. For tractography, the number of streamlines is a number which reflects the number of connections between brain regions, which is expected to be related to the amount of information that can be transmitted from one region to another (Rolls, 2021a; Rolls & Treves, 2011).

## 3 RESULTS

### 3.1 Effective connectivity, functional connectivity, and diffusion tractography

**FIGURE 2:**
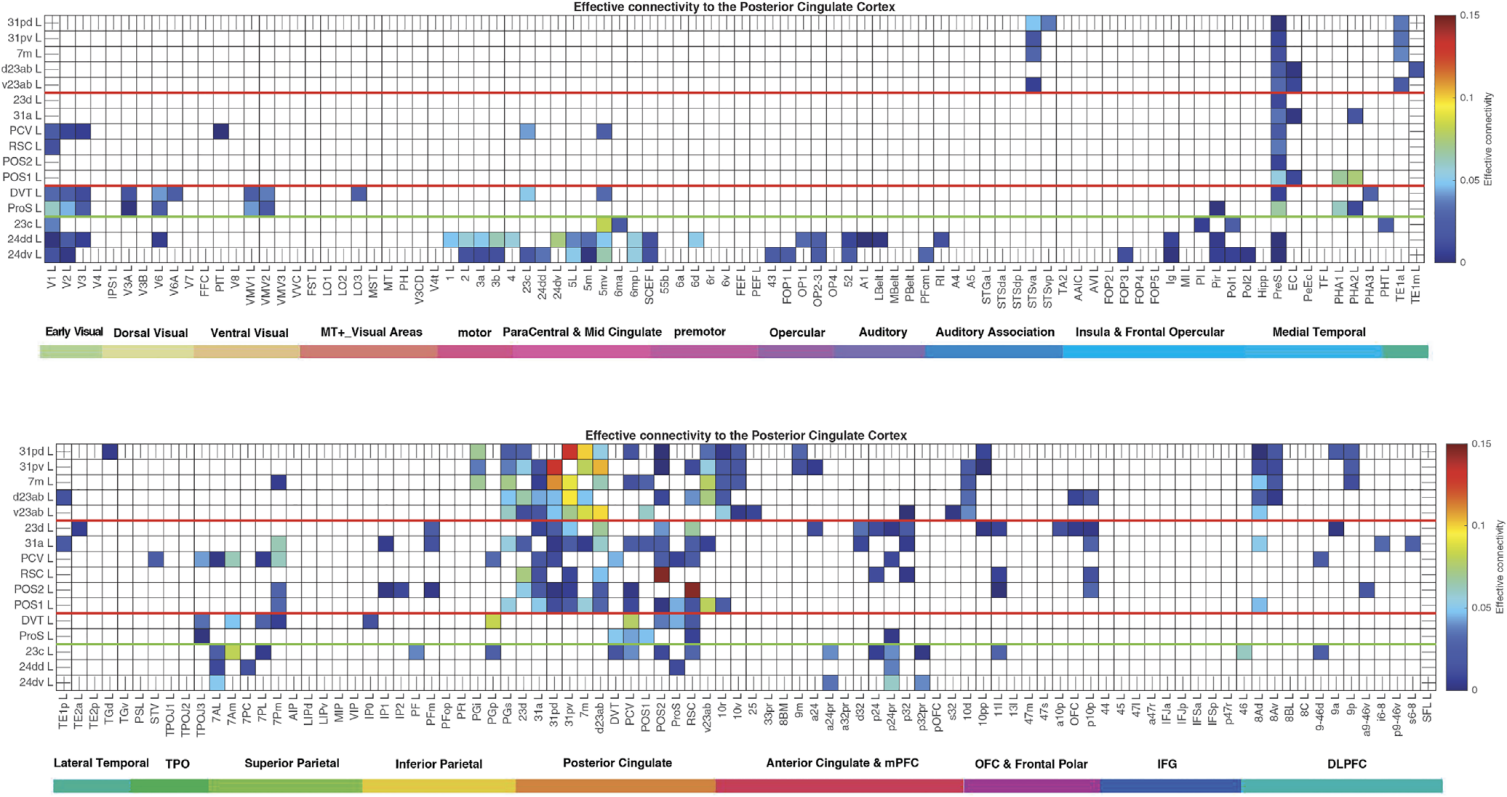
Effective connectivity *TO* the posterior cingulate cortex division regions (the rows) *FROM* 180 cortical areas (the columns) in the left hemisphere. The effective connectivity is read from column to row. Effective connectivities of 0 are shown as blank. All effective connectivity maps are scaled to show 0.15 as the maximum, as this is the highest effective connectivity found between this set of brain regions. The effective connectivity algorithm for the whole brain is set to have a maximum of 0.2, and this was for connectivity between V1 and V2. Abbreviations: See Table S1. The three groups of posterior cingulate cortex division areas as defined in HCP‐MMP1 are separated by red lines; and the MCC functional connectivity is shown below the green line. The upper matrix is for the connectivity from the first 90 cortical regions listed in Table S1, and the lower matrix is for the next 90 regions listed in Table S1.

**FIGURE 3:**
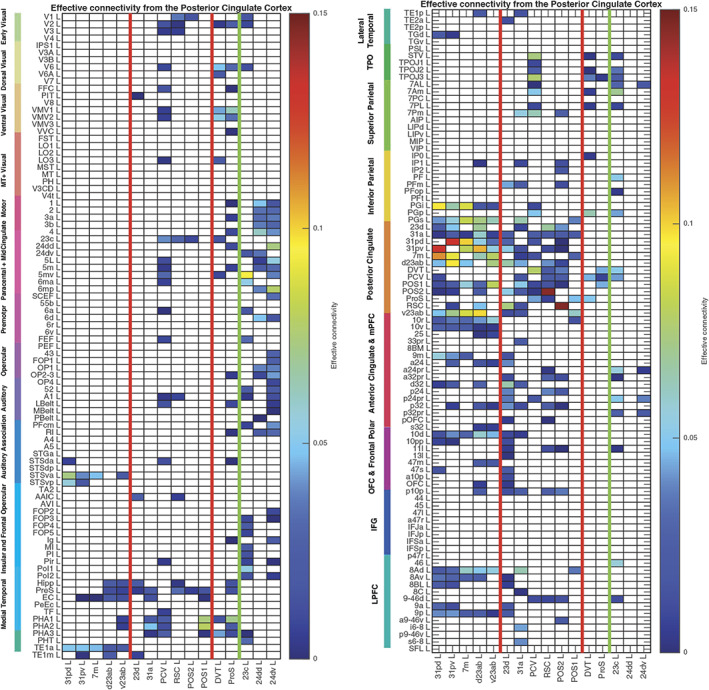
Effective connectivity *FROM* the posterior cingulate cortex division regions *TO* 180 cortical areas in the left hemisphere. The effective connectivity is read from column to row. Effective connectivities of 0 are shown as blank. Abbreviations: See Table S1. The three groups of posterior cingulate cortex division areas are separated by red lines; and the MCC functional connectivity is shown to the right of the green line. The left matrix is for the connectivity to the first 90 cortical regions listed in Table S1, and the right matrix is for the next 90 regions listed in Table S1.

The effective connectivities to the posterior cingulate cortex/RSC/medial parietal cortex from other cortical areas in the left hemisphere are shown in Figure 2. The effective connectivities from the posterior cingulate cortex division to other cortical areas in the left hemisphere are shown in Figure 3. Differences for the right hemisphere are considered later in the Section 3 and in Figures S8, S9, S2, and S3. All Figures include the MCC regions outside the green line. The vectors of effective connectivities of each of the 16 cingulate cortex ROIs with the 180 areas in the modified HCP atlas were all significantly different from each other. (Across the 171 participants the interaction term in separate 2‐way ANOVAs for the comparisons between the effective connectivity of every pair of the 13 posterior cingulate division ROIs after Bonferroni correction for multiple comparisons were all *p* < 10^−90^. The results were confirmed with the nonparametric Scheirer–Rey–Hare test [Scheirer et al., 1976; Sinha, 2022].) The functional implications of the results described next are considered in Section 4, the discussion.

**FIGURE 4:**
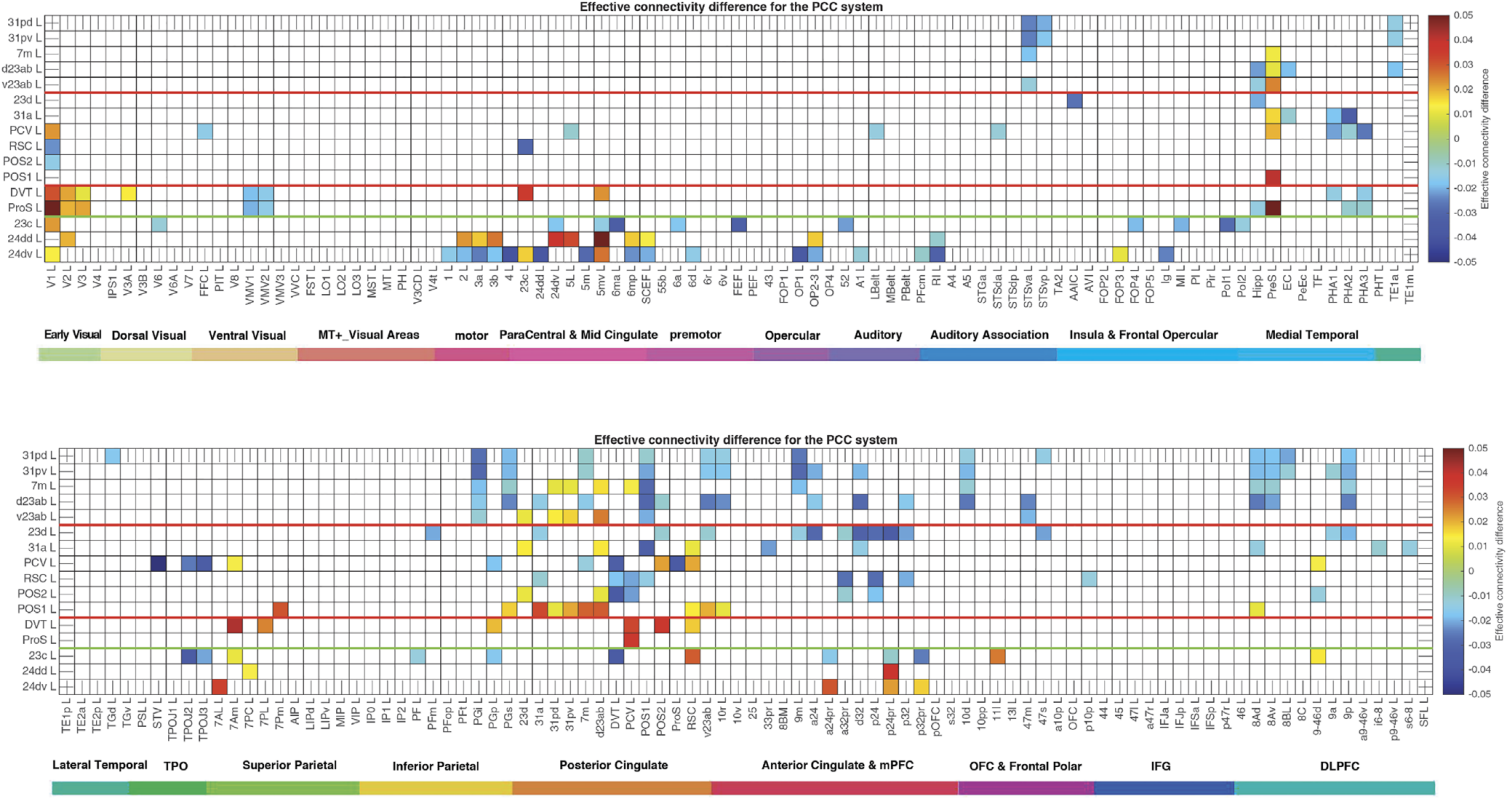
Difference of the effective connectivity in the two directions for each link for the posterior cingulate division regions with cortical regions. For a given link, if the effective connectivity difference is positive, the connectivity is stronger in the direction from column to row. For a given link, if the effective connectivity difference is negative, the connectivity is weaker in the direction from column to row. This is calculated from 171 participants in the HCP imaged at 7T. The threshold value for any effective connectivity difference to be shown is 0.01, to enable this figure to show only the larger differences in the effective connectivities in the two directions. The abbreviations for the brain regions are shown in Table S1, and the brain regions are shown in Figures 1 and S1. The effective connectivity difference for the first set of cortical regions is shown above; and for the second set of regions below.

**FIGURE 5:**
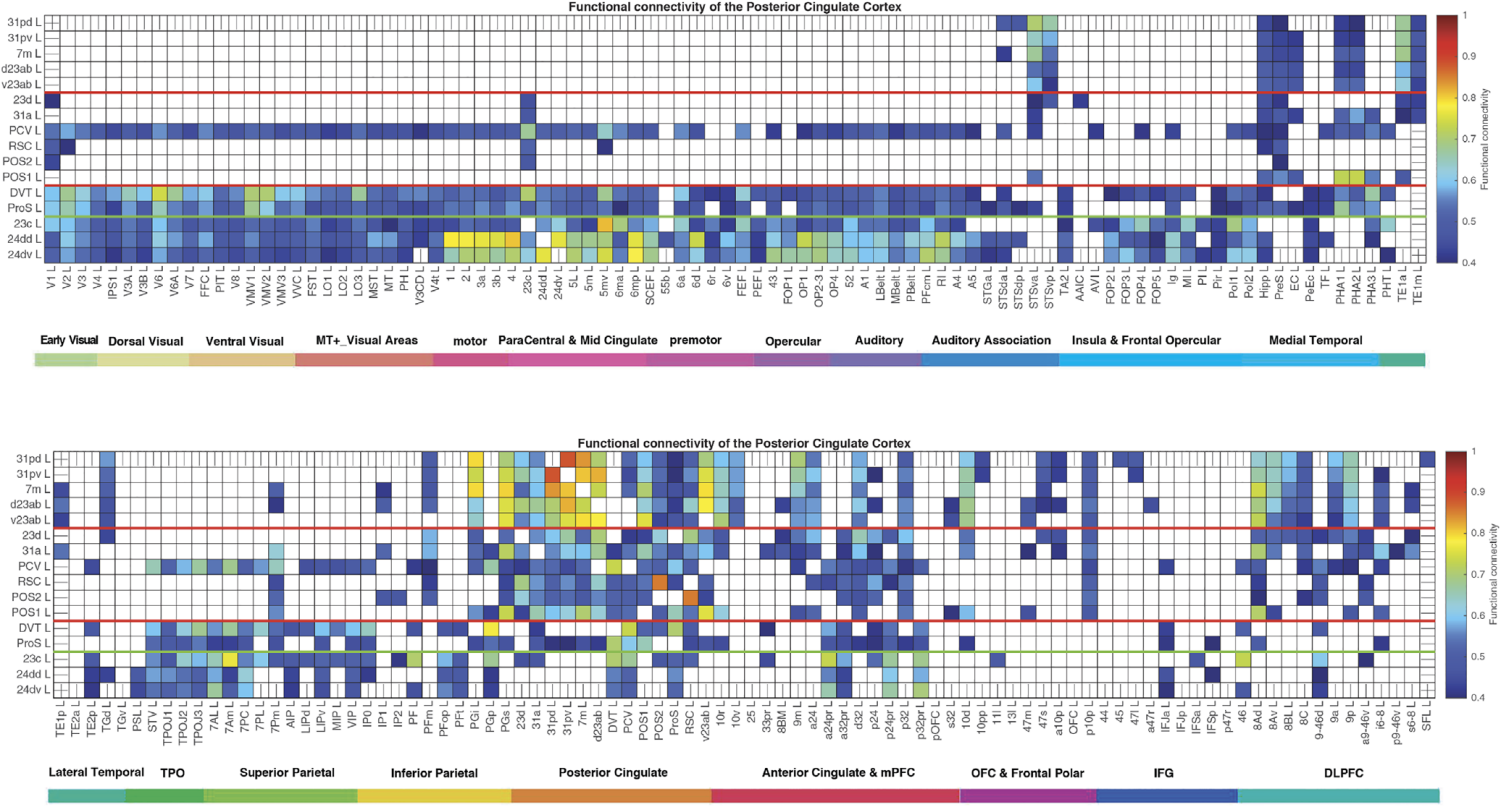
Functional connectivity between the posterior cingulate cortex division regions and 180 cortical areas in the left hemisphere. Functional connectivities <0.4 are shown as blank. The upper figure shows the functional connectivity of the 16 regions with the first half of the cortical areas (including the subiculum); the lower figure shows the functional connectivity with the second half of the cortical areas. Abbreviations: See Table S1. The three groups of posterior cingulate cortex division regions are separated by red lines; and the MCC functional connectivity is shown below the green line.

**FIGURE 6:**
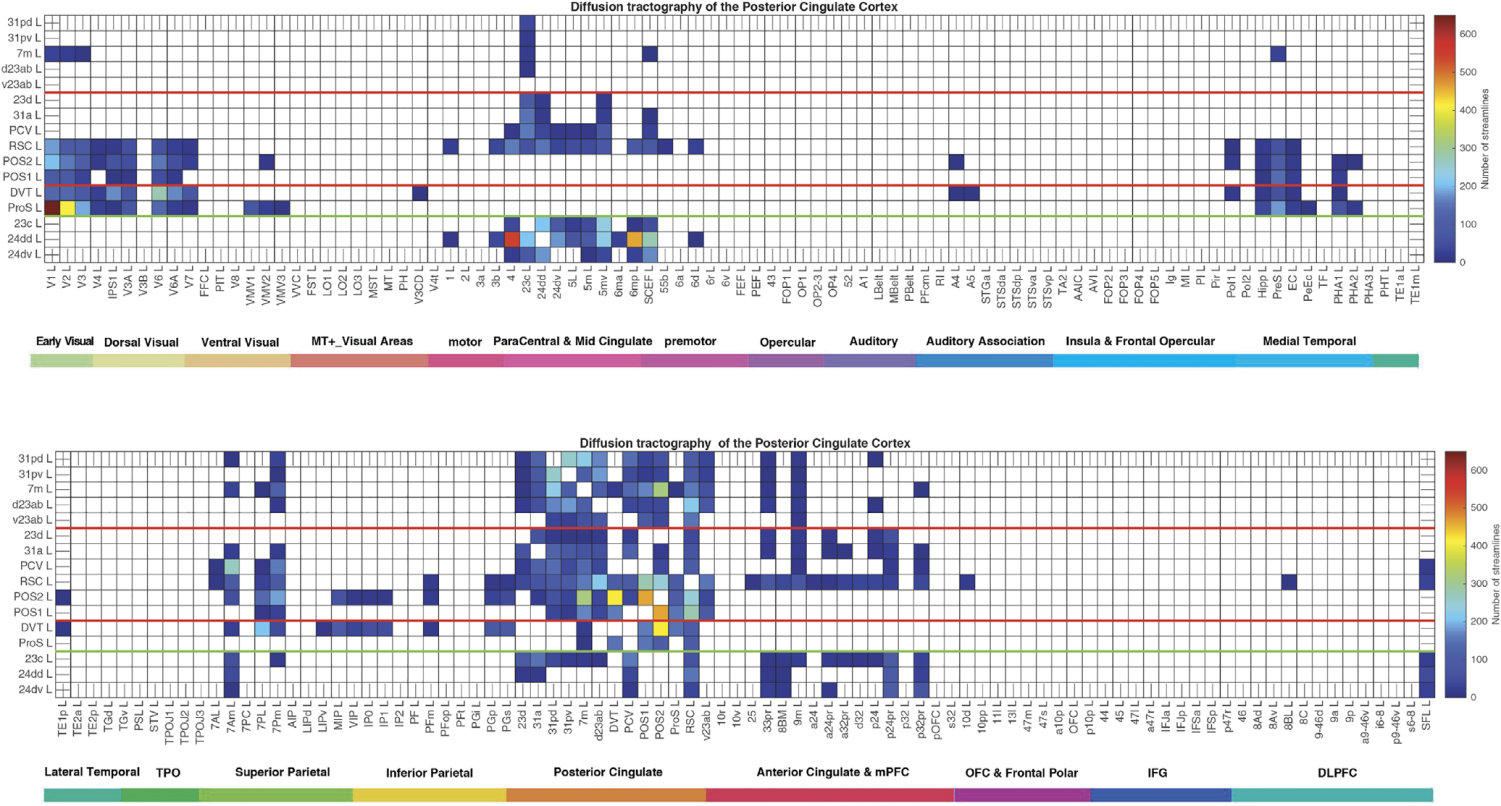
Connections between the posterior and MCC and 181 cortical areas in the left hemisphere as shown by diffusion tractography using the same layout as in Figures 2 and 4. The number of streamlines shown was thresholded at 10 and values less than this are shown as blank. Abbreviations: See Table S1. The three groups of posterior cingulate cortex areas are separated by red lines; and the MCC connections are shown below the green line.

The 13 HCP‐MMP cortical regions included in this HCP‐MMP1 atlas as part of the posterior cingulate cortex division considered here are grouped for ease of description into three groups (1–3) shown in Figure 1a–c as described above. To facilitate the description of the results, each of these groups is described in turn and separated by red lines in the figures, which include the effective connectivities shown in Figures 2 and 3, the difference of the effective connectivities in the two directions for every link (Figure 4), the functional connectivities (Figure 5), and the diffusion tractography (Figure 6). In addition, to enable an explicit comparison, the results for the MCC, which in the HCP‐MMP atlas are 23c, 24dd, and 24dv, are included beyond the green line in the figures. These groups are used to help present the findings, but different HCP‐MMP regions within a group do not have identical connectivity, and this shows part of the utility of the HCP‐MMP atlas (Glasser, Coalson, et al., 2016a; Huang et al., 2022) and the approach taken here. For example in Group 1, the connectivity of region 31pd is correlated with that of most but not all other members of the group, as shown below (Figure S4). The description starts with the left hemisphere, which is of especial interest as it is involved in language, but there is a comparison with connectivity in the right hemisphere later in the Section 3 and in Figures S8, S9, S2, and S3.

### 3.2 Group 1, postero‐ventral posterior cingulate division, regions 31pd, 31pv, 7m, d23ab, and v23ab

The topological terms such as “postero‐ventral” are based on the locations of the area 31 and 23 components in Group 1, which are the areas that define anatomically the posterior cingulate cortex, because these topological terms are sometimes used to describe the different parts of the posterior cingulate cortex, as is evident in Section 1.

This group is characterized (Figures 2 and 3) by effective connectivity with the temporal lobe visual association areas (especially TE1a), superior temporal sulcus (STS) auditory–visual association cortex (region STSva; Rolls et al., 2022b; Rolls et al., 2022d), the medial temporal lobe hippocampal system (presubiculum, entorhinal cortex, hippocampus, and parahippocampal PHA1–PHA2), superior parietal (7m with 7Pm), inferior parietal (PGi and PGs), reward‐related regions including the pregenual anterior cingulate cortex (9m, a24, d32, p32, and 10d and ventromedial prefrontal cortex 10r and 10v; Rolls et al., 2022e), subgenual anterior cingulate cortex (25), and dorsolateral prefrontal cortex regions (8Ad, 8Av, and 9p). Many of these connectivities are stronger from the PCC to these cortical regions, but the presubiculum has a stronger effective connectivity to the PCC (Figure 4).

The functional connectivity (FC) is generally consistent (Figure 5), with FC also or more evident with STS regions, with the hippocampus, parahippocampal TH regions PHA1–PHA2, with temporal pole TGd as well as inferior temporal visual TE areas, with parietal PFm which is a visual region as well as PGi and PGs (Rolls et al., 2022a), and dorsolateral prefrontal cortex (8Ad, 8Av, 8C, 9a, 9p, and i6‐8; Rolls et al., 2022c).

The diffusion tractography (Figure 6) provides evidence for direct connections with the presubiculum, superior parietal cortex (7Pm, 7Am), and anterior cingulate cortex.

Topologically, as shown in Figures 1a, 8, and S1 (e.g., at MNI coordinates Y = −46 and −54), regions 31pd, 31pv, d23ab, and v23ab tend to be posterior in the PCC, with 7m just posterior to these regions.

### 3.3 Group 2, antero‐dorsal posterior cingulate division regions 23d, 31a, PCV, and RSC, POS2, and POS1

POS1 and POS2 are visual areas in the parieto‐occipital sulcus close to the primary visual cortex V1 (Figures 1b and 8), have extensive connections (Figure 6) and some functional connectivity (Figure 5) with early visual cortical areas, and have effective connectivity with the other brain regions in Group 2, 23d, 31a, PCV, and RSC (Figure 2). The precuneus visual area PCV also has interactions with early visual cortical areas as shown by the functional connectivity (Figure 5).

The Group 2 regions between them have effective connectivity to the parahippocampal gyrus regions PHA1‐3 (which correspond to macaque TH) and to the hippocampal system (including the hippocampus, entorhinal cortex, and presubiculum) as shown in Figure 3, with supporting evidence from the functional connectivity (Figure 5) and tractography (Figure 6).

Group 2 includes the precuneus visual area (PCV, *Y* = −45 to −54) which is dorsal, with 31a anterior to PCV at *Y* = −30 and −38 (Figure 1b), and 23d anterior to 31a. In addition to the POS1 and POS2 inputs and connectivity to TH and the hippocampal system, this set of brain regions has effective connectivity with the temporo‐parietal‐occipital junction (*which is activated during theory of mind*, *is implicated in the self‐other distinction*, *etc*. [Buckner & DiNicola, 2019; DiNicola et al., 2020; Quesque & Brass, 2019]); the superior parietal cortex (especially 7Pm and 7Am which are medial); frontal pole regions p10p and a10p; the reward‐related pregenual anterior cingulate d32, p24, p32, and medial orbitofrontal cortex (regions 11l, 13l, and OFC); MCC 23c; and dorsolateral prefrontal cortex including 8Ad, 8Av, 9‐46d, 9a, and 9p. Many of these connectivities are stronger from the PCC to these cortical regions, but 7Pm and 7Am, PGs, and 10r have stronger effective connectivity to Group 2 of the PCD (Figures 3 and 4).

Region 23d just anterior to 31a has effective connectivity with inferior parietal PFm; with the other Group 2 regions 31a, RSC, and POS2; with reward‐related orbitofrontal cortex OFC, pOFC, 11l, and 13l, with pregenual anterior cingulate d32, p32, a24, and p24, and with ventromedial prefrontal cortex (vmPFC) 10d and 9m (see Rolls et al. 2022e); with supracallosal anterior cingulate a32pr *where mainly aversive stimuli and responses they may elicit are represented* (Bush et al., 2000; Grabenhorst & Rolls, 2011; Kringelbach & Rolls, 2003; O'Doherty et al., 2001; Rolls et al., 2003; Sturm et al., 2013); with parts of frontal pole area 10 (a10p and p10p); and with the dorsolateral prefrontal cortex (Figure 2). The region that 23d is most similar to in terms of its connectivities with other brain regions is RSC (Figure S4). The functional connectivity (FC) is generally consistent (Figure 5), with FC also or more evident with V1, inferior parietal PF, PGi, and PGs, and several dorsolateral prefrontal cortex regions.

The diffusion tractography (Figure 6) provides evidence for direct connections of RSC, POS2, and often POS1 with many visual cortical areas (including V1‐V3A, V6, V6A, and V7); with superior and inferior parietal regions (7Pm, 7Am, 7PL, PFm, PGi, and PGs); and of RSC with pregenual anterior cingulate regions and vmPFC region 10d. The diffusion tractography does not show a direct connection with frontal pole p10p and most dorsolateral prefrontal cortex regions, suggesting that these connectivities are indirect.

**FIGURE 7:**
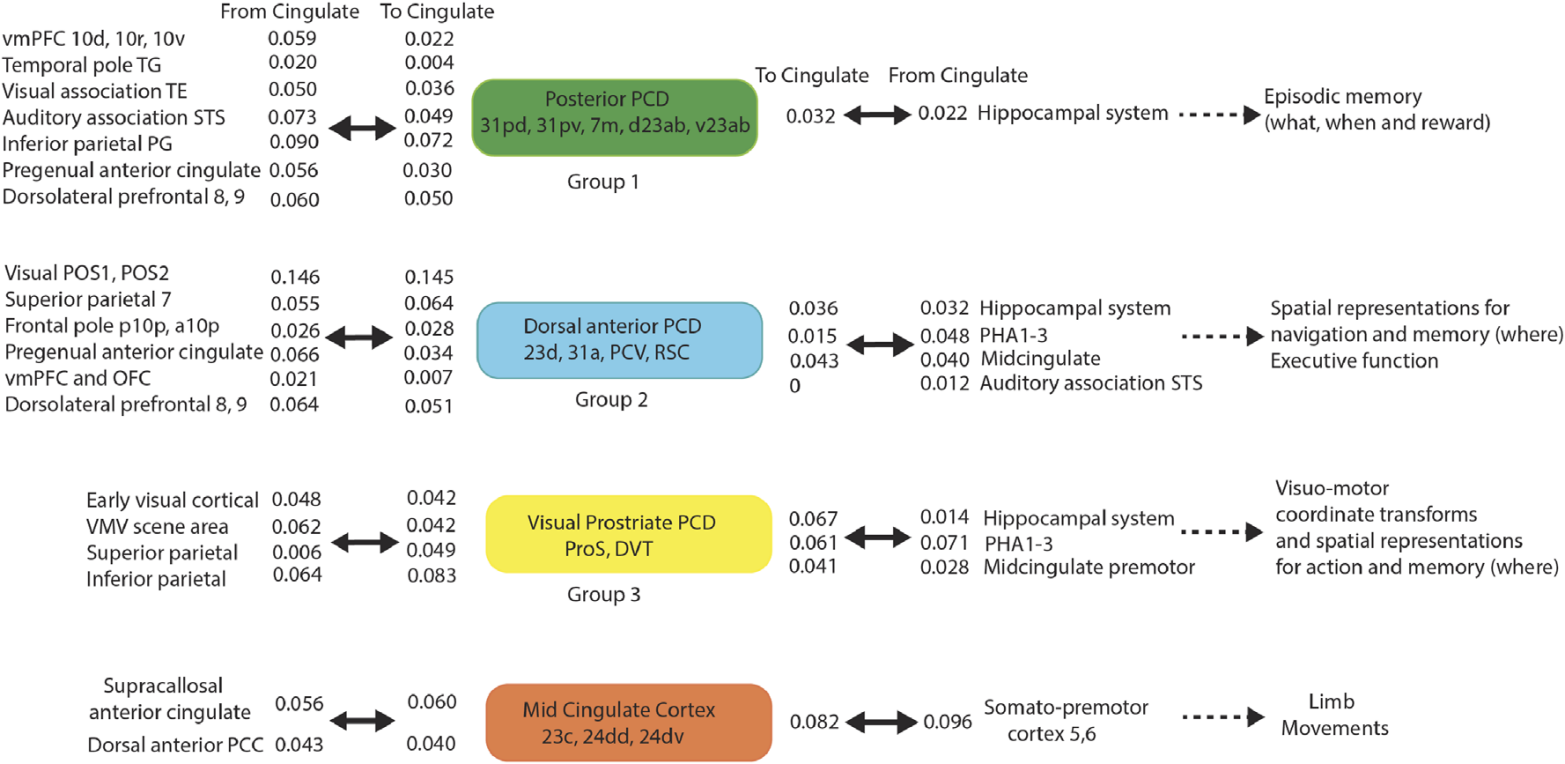
Synthesis of the connectivity of the posterior cingulate cortex division regions (see text). The effective connectivities indicated by numbers show the strength of the effective connectivity directed to or from cingulate regions for other cortical regions or groups of cortical regions. The effective connectivities shown are for the strongest link where more than one link between regions applies to a group of brain regions. The diagram shows how posterior cingulate division regions and the MCC provide routes for other cortical regions on the left to connect to hippocampal and premotor regions shown on the right. The Group 2 regions include also POS1 and POS2 which provide visual inputs into the other Group 2 named regions. DVT, dorsal visual transitional region; OFC, orbitofrontal cortex; PCC, posterior cingulate cortex; PCV, precuneus visual area; ProS, ProStriate visual region; RSC, retrosplenial cortex; STS, superior temporal sulcus auditory association cortex; TH, parahippocampal cortex represented in HCP‐MMP by PHA1‐3; vmPFC, ventromedial prefrontal cortex; VMV, ventromedial visual region with visual scene representations. Other abbreviations are in Table S1.

**FIGURE 8:**
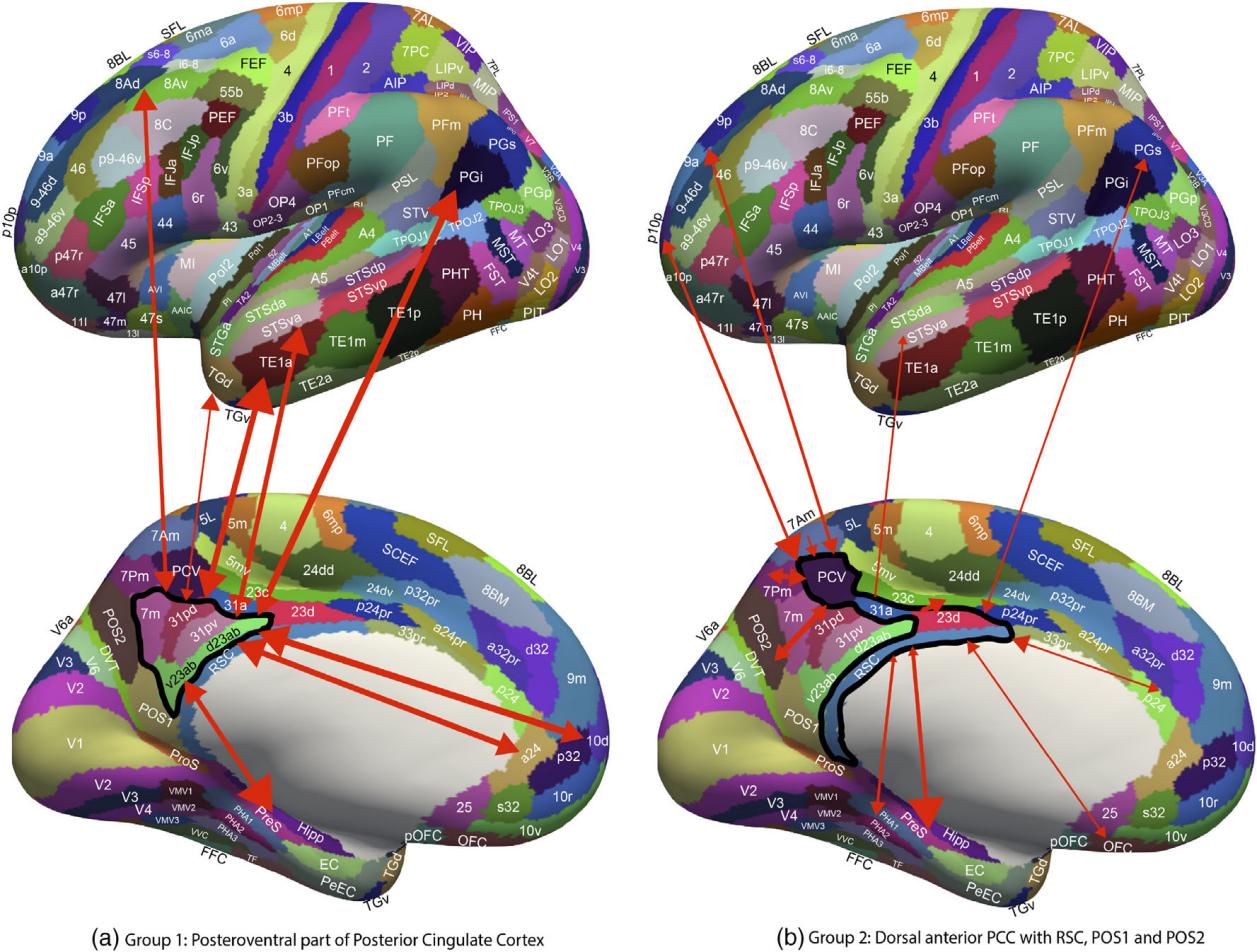
(a) Summary of connectivity of Group 1 regions, the posteroventral part of the posterior cingulate cortex division shown on medial (below) and lateral (above) views of the human brain, with the sulci expanded to show regions inside the sulci. The Group 1 regions (within the black boundary) are v23ab and d23ab, 31pd and 31pv, and 7m. The width of the lines reflects the effective connectivity in the strongest direction, and the size of the arrowheads reflects the effective connectivity in each direction. The labels are those for the cortical regions in the HCP‐MMP1/HCPex atlas shown in Table S1. The Group 1 regions have effective connectivity with the hippocampal system (presubiculum, subiculum, entorhinal cortex, and hippocampus); anterior cingulate cortex (a24); frontal pole 10d; inferior parietal cortex PGi and PGs; the auditory cortex in the superior temporal sulcus (STS); the anterior inferior temporal cortex (TE1a); the dorsolateral prefrontal cortex 8Ad; and the temporal pole TGi. (b) Summary of the effective connectivity of Group 2 regions, which include the more anterior and dorsal parts of the posterior cingulate cortex shown on medial (below) and lateral (above) views of the human brain, with the sulci expanded to show regions inside the sulci. The brain regions in Group 2 include POS1 and POS2 (early visual cortical areas), which provide inputs to the other members of the group (surrounded by a black boundary), PCV, 31a, 23d, and the retrosplenial cortex (RSC). The width of the lines reflects the effective connectivity in the strongest direction, and the size of the arrowheads reflects the effective connectivity in each direction. Visuo‐spatial inputs are also received from medial parietal cortex areas 7Pm and 7Am. Group 2 areas also have connectivity with the pregenual anterior cingulate cortex p24 and d32, with region OFC, with parietal PGs, with frontal pole p10p, and with dorsolateral prefrontal cortex 9p. Outputs of Group 2 regions are directed to the hippocampal system, including the hippocampus, presubiculum, and parahippocampal TH (PHA1‐3); and to the midcingulate motor cortex 23c.

Differences of the Group 2 regions from the Group 1 regions (summarized in Figures 7 and 8) are that parts of Group 2 have extensive diffusion tractography with early visual cortical areas which are reflected in the functional connectivity; more effective connectivity with the superior parietal cortex area 7; with the frontal pole areas p10p implicated in executive function and sequencing of behavior; and to the MCC 23c, and related areas 5 and 6. In contrast, the Group 1 regions have effective connectivity with visual temporal association cortex TE areas; and higher effective connectivity with temporal lobe auditory association cortical areas including STSva and STSda. Importantly, the Group 2 areas are not all located together, with some (PCV, 31a, and 23d) dorsal and somewhat anterior in PCC (see Figure 1b), but POS1 and POS2 are further posterior close to early cortical visual areas (see Figure 1b). It may be that POS1 and POS2 should be considered as related to early visual cortical areas, rather than what is usually subsumed within the PCC/RSC (Glasser, Coalson, et al., 2016a), but provide key inputs to the Group 2 regions.

### 3.4 Group 3, DVT and ProS

The ProS region is adjacent to V1, and the DVT area (DVT) is an area posterior to most of the other regions in the Posterior Cingulate Division, found just lateral to POS2 (Figures 1c, 8, and S1‐4). These areas have effective and functional connectivity and connections with visual cortical areas including V1–V3, V6, and V6a; with the ventromedial visual (VMV) areas and parahippocampal TH regions PHA1‐3 involved in scene representations (Sulpizio et al., 2020); with the presubiculum and hippocampus; with the midcingulate (premotor) cortex; with temporo‐parieto‐occipital junction (TPOJ) regions; and (especially for DVT) with parts of the superior (7Am, 7PL) and inferior parietal cortex (PGp). Many of these effective connectivities are stronger toward DVT and ProS, but these regions have connectivity directed more strongly toward the parahippocampal gyrus PHA1‐3 (Figures 2, 3, 4). The diffusion tractography provides evidence that many of these are direct connections (Figure 6). The functional connectivity shows interactions not only with visual cortical areas, but also with somatosensory and auditory regions (Figure 5). DVT and ProS thus have connections with early cortical visual regions, with a number of areas representing visual scenes (PHA1‐3 and VMV regions as shown by Sulpizio et al. [2020] whose study is especially relevant as it used the HCP‐MMP1 atlas), with the superior parietal cortex, with hippocampal system regions, and with the premotor MCC.

These Group 3 regions differ from Group 1 regions (see summary in Figure 7) in having effective connectivity with early visual cortical areas, with the midcingulate premotor cortex, with more parts of the superior parietal cortex, in having connectivity with inferior parietal PGp instead of PGi, in having little connectivity with Group 1 regions, and in having no effective connectivity with the inferior temporal visual cortex (TE), frontal pole area 10p or with the dorsolateral prefrontal cortex (Figure 2). The Group 3 regions differ from Group 2 regions in having more effective connectivity with early cortical visual areas; and in having no effective connectivity with the inferior temporal visual cortex (TE), frontal pole area 10p, or with dorsolateral prefrontal cortex regions (Figure 2).

### 3.5 Midcingulate cortex, 23c, 24dd, and 24dv

The MCC or cingulate motor area is included here so that it can be contrasted with the Posterior Cingulate Division, and so that its connections with the PCC can be shown in detail. The MCC has some effective connectivity from V1 and V2 (Figure 2), no direct connections shown by diffusion tractography (Figure 6), and some functional connectivity with a range of early visual cortical areas (Figure 5). It has strong effective connectivity with premotor and related cortical regions 6 (premotor) and 5 (somatosensory), with parts of area 5 connecting toward the MCC, Figure 4, and with parts of the insula that have somatosensory representations; with the supracallosal anterior cingulate cortex (a24pr, a32pr, p24pr, and p32pr) which projects (with 11l) toward the MCC (Figure 4); and with dorsolateral prefrontal cortex 9‐46d. The MCC thus has much connectivity to somatomotor cortical areas, and receives input from the supracallosal (supragenual) anterior cingulate cortex.

It is shown in Figure 6 that especially the supracallosal anterior cingulate cortex has connections with midcingulate motor areas 23c, 24dd, 24dd; and so does SCEF the Supplementary and Cingulate Eye Field. The MCC also has connections with PCV and DVT (Figure 6), with some evidence too in the effective and functional connectivity. The greater effective connectivity of the MCC with the supracallosal anterior cingulate cortex than with the pregenual anterior cingulate is emphasized by the effective connectivity (Figure 2), showing how effective connectivity may be able to go beyond connections to functional strength.

### 3.6 Effective connectivities of the posterior cingulate and medial parietal cortex with contralateral cortical regions

The effective connectivities of the posterior cingulate division and MCC from contralateral cortical areas are shown in Figure S2, and in contralateral cortical areas in Figure S3. The contralateral effective connectivities are in general weaker than those ipsilaterally. (The ratio across the matrices shown in Figures 2 and S2 was that the contralateral effective connectivities were 60% of the ipsilateral effective connectivities.) The overall similarity of the effective connectivity links contralaterally compared with ipsilaterally, and the fact that each posterior cingulate division region had high connectivity with the corresponding region contralaterally, attest to the power of the effective connectivity algorithm in detecting corresponding particular brain regions contralaterally as well as ipsilaterally.

### 3.7 Effective connectivities of the posterior cingulate and medial parietal cortex with subcortical regions

The effective connectivities of subcortical regions in the HCPex atlas (Huang et al., 2022) to the Posterior Cingulate Division cortical regions are shown in Figure S6. Thalamic inputs are received from the antero‐ventral nucleus (AV) which is as expected for cingulate cortex areas (Bubb et al., 2017). In addition, for the Group 2 regions which have connectivity with early visual cortical areas, there is strong effective connectivity from (and to, see Figure S7) the medial pulvinar nucleus (PuM) which has visual and other functions (Froesel et al., 2021), and this has been overlooked (Bubb et al., 2017). Very interestingly, the Group 1 regions associated with hippocampal function receive effective connectivity from the mammillary bodies (MB), and the septum and basal nucleus of Meynert which contain cholinergic neurons. There is evidence that most of this connectivity is bilateral.

The MCC in contrast receives effective connectivity from thalamic nuclei ventral postero‐lateral (VPL) and central‐median (CM), with also some effective connectivity from the pulvinar. Interestingly, there is no effective connectivity detected from the septum and nucleus basalis, suggesting that cholinergic inputs are directed especially to the part of the posterior cingulate cortex, the Group 1 regions, that are associated with hippocampal memory functions as shown in the Discussion (Section 4).

These patterns of effective connectivities with subcortical areas shown in Figures S6 and S7 again provide evidence for the robustness and utility of the Hopf effective connectivity algorithm (Rolls et al., 2022f) used here, even when used to measure effective connectivities between every pair of 426 brain regions.

### 3.8 Differences in effective connectivities of the right versus left hemisphere for the posterior cingulate cortex

Most of the analysis presented so far has been for the left hemisphere (Figures 2, 3, 4, 5, 6), or for the left with the right hemisphere (Figures S2 and S3). For completeness, comparisons with the connectivities of the right hemisphere are shown in Figures S8 and S9. The comparison shows the differences in effective connectivity for the Right *minus* the left hemisphere for the posterior cingulate cortex/RSC regions. The differences between the hemispheres were overall small, but some differences are interesting to note. For example, some of the effective connectivities involving the parietal cortex with the posterior cingulate cortex are stronger in the right hemisphere, consistent with the probability that there is more spatial processing in the right hemisphere. Conversely, connectivity of parts of the vmPFC (10r, 10v, 10d) involved in reward with the posterior cingulate cortex was stronger in the left hemisphere (Figure S9) with the background considered elsewhere (Rolls et al., 2022e). For completeness, the effective connectivities for the right hemisphere are shown in Figures S10 and S11 for direct comparison with the effective connectivities for the left hemisphere shown in Figures 2 and 3.

## 4 DISCUSSION

Some of the key findings are summarized next, with details for each group considered later, with summaries provided in Figures 7 and 8.

First, a postero‐ventral part of the Posterior Cingulate Division (31pd, 31pv, 7m, d23ab, and v23ab, grouped because of similar connectivity) has effective connectivity with the temporal pole, inferior temporal visual, and superior temporal auditory association cortex, with the reward‐related vmPFC and pregenual anterior cingulate cortex, with the inferior parietal cortex, and with the hippocampal system. We propose below that this connectivity implicates it in hippocampal episodic memory, providing routes for “what,” “when” (including temporal order), reward, and semantic schema‐related information to access the hippocampus.

Second, the antero‐dorsal parts of the posterior cingulate and medial parietal cortex (especially 31a and 23d, and more posteriorly PCV and RSC) have connectivity with early visual cortical areas including those that represent spatial scenes, with the superior parietal cortex, with the pregenual anterior cingulate cortex, and with the hippocampal system. We propose below that this connectivity implicates it in the “where” component for hippocampal episodic memory and for spatial navigation, for which the orbitofrontal/pregenual anterior cingulate system may provide the goals.

Third, the DVT region and ProS regions receive from early visual cortical areas, the superior parietal cortex (7Am, 7PI), and connect to the midcingulate premotor cortex. These regions are where the retrosplenial scene region is located in the HCP‐MMP atlas (Sulpizio et al., 2020). This connectivity implicates these two regions in scene processing for memory and navigation, and in visuo‐motor control of actions in visual space and the spatial coordinate transforms necessary for this and navigation.

In contrast, the midcingulate premotor cortex has connectivity with somatomotor cortical areas, receives input from the supracallosal anterior cingulate cortex which is implicated in action‐outcome learning, and may implement goal‐directed limb actions.

### 4.1 Group 1, postero‐ventral posterior cingulate and medial parietal regions 31pd, 31pv, d23ab, v23ab, and 7m, and their relation to episodic memory

This group has connectivity with ventral stream high‐order visual (TE) and auditory (STS) association cortical areas that represent what object or person is present as shown by discoveries in macaques (Arcaro & Livingstone, 2021; Booth & Rolls, 1998; Freiwald, 2020; Lehky & Tanaka, 2016; Perrett et al., 1982; Rolls, 2021a, 2021c; Rolls et al., 2022d) with complementary evidence in humans (Collins & Olson, 2014; Finzi et al., 2021; Kanwisher et al., 1997); with the pregenual anterior cingulate cortex (9m, a24, d32, p32, and 10d) and vmPFC (10r and 10v) where reward value and emotion are represented (Grabenhorst & Rolls, 2011; Rolls, 2019a, 2022a; Rolls et al., 2022e); with some inferior parietal cortex regions (PGi and PGs, which are in the angular gyrus BA 39 and are implicated in memory and semantic processing [Davis et al., 2018; Papagno, 2018; Rolls et al., 2022a]); with temporal pole (TG) regions implicated in semantic memory (Bonner & Price, 2013; Rolls et al., 2022b); with frontal pole regions (10pp, p10p); and with the hippocampal system which is involved in episodic memory (Dere et al., 2008; Ekstrom & Ranganath, 2018; Moscovitch et al., 2016; Rolls, 2018, 2021a). The connections to this set of brain regions suggest that this ventral and posterior part of the PCC is involved in hippocampal episodic memory, providing routes for the relevant “what,” “where,” “when” (including temporal order), reward, and semantic schema‐related information to gain access to the hippocampus during storage; and to be on the route back to these cortical areas during hippocampal episodic memory retrieval (Rolls, 2021a, 2022a). The Group 1 regions are thus especially related to ventral stream “what” processing (Ungerleider & Haxby, 1994), and allow many widely separated brain regions at the top of processing streams to provide “what,” “when,” and reward inputs to the hippocampal system. Consistent with the involvement of Group 1 regions in linking reward and emotion systems to memory, the precuneus has increased functional connectivity in depression with the lateral orbitofrontal cortex non‐reward/punishment system (Cheng et al., 2018).

The relation of Group 1 PCC regions to memory is strengthened by the evidence in Figure S6 that these Group 1 region associated with hippocampal function receive effective connectivity from the mammillary bodies (MB) which are part of a hippocampal circuit involved in episodic memory (Bubb et al., 2017), and the septum (which projects to the hippocampal system) and adjoining basal nucleus of Meynert both of which contain cholinergic neurons which are implicated in memory (Hasselmo & Giocomo, 2006; Rolls, 2022a; Rolls & Deco, 2015).

### 4.2 Group 2, antero‐dorsal posterior cingulate division regions 23d, 31a, PCV; and RSC, POS2, and POS1; and their relation to navigation and executive function

POS1 and POS2 are visual areas in the parieto‐occipital sulcus close to the primary visual cortex V1 (Figure 8), have extensive connections (Figure 6) and functional connectivity (Figure 5) with early visual cortical areas, and provide inputs to the other brain regions in this group, 23d, 31a, PCV, and RSC (Figure 2). Further, POS1 and POS2 also have effective connectivity to the medial parahippocampal gyrus PHA1‐3 regions (corresponding to TH) where scenes are represented (Epstein, 2005, 2008; Epstein & Baker, 2019; Epstein & Julian, 2013; Epstein & Kanwisher, 1998; Kamps et al., 2016; Natu et al., 2021; Rolls, 2022b; Sulpizio et al., 2020), and with the hippocampal system (Figure 2). POS1 and POS2 may thus be involved in scene representations reaching the hippocampal system, and are likely to contribute to the activation of hippocampal spatial view cells, and thereby be involved in providing information important in episodic memory and navigation (Rolls, 2022b; Rolls & Wirth, 2018). POS1 and POS2 are also likely to be involved in memory and navigation by providing inputs to other members of the Group 2 regions as follows.

Anterior to POS1 and POS2 and receiving visual inputs from these regions are the precuneus visual area (PCV) and 31a and 23d which are dorsal and extend anteriorly in the Posterior Cingulate Division (Figures 1b and 8) and have connectivity directed to the hippocampal system (hippocampus, entorhinal cortex, and presubiculum), to the parahippocampal PHA1‐3 regions (Figure 3) with visual scene representations (Epstein & Kanwisher, 1998; Kamps et al., 2016; Natu et al., 2021; Rolls et al., 2022d; Sulpizio et al., 2020), and the MCC. They receive effective connectivity not only from POS1 and POS2, but also from the temporo‐parietal junction (a multimodal region implicated in social behavior and language [Buckner & DiNicola, 2019; Coslett & Schwartz, 2018; DiNicola et al., 2020; Patel et al., 2019; Quesque & Brass, 2019; Rolls et al., 2022b]); the superior parietal cortex (7Pm and 7Am; Rolls et al., 2022a); frontal pole regions p10p and a10p implicated in planning and sequencing (Gilbert & Burgess, 2008; Shallice & Burgess, 1996; Shallice & Cipolotti, 2018) and prospective as well as retrospective memory (Underwood et al., 2015); the pregenual anterior cingulate including d32, p24, and p32 implicated in reward (Grabenhorst & Rolls, 2011; Rolls, 2022a; Rolls et al., 2022e); and dorsolateral prefrontal cortex 8Ad, 9a, 9p involved in short‐term memory and thereby top‐down attention (Deco & Rolls, 2005; Germann & Petrides, 2020; Rolls et al., 2022c). This part of the Posterior Cingulate Division is special in its strong connectivity with p10p which is part of the frontal pole cortex (Figure S1‐1, *y* = 66); the reward‐related pregenual anterior cingulate d32, p24, p32, and medial orbitofrontal cortex (regions 11l, 13l, and OFC; Rolls et al., 2022e); and the midcingulate premotor area (see Figure 2). This connectivity including the connectivity to the parahippocampal scene or place area in PHA1‐3 implicates Group 2 regions in spatial and executive function (cf. Buckner & DiNicola, 2019; Dastjerdi et al., 2011; Foster et al., 2012, 2013; Fox et al., 2018). Spatial roles for this part of the PCC are emphasized too by the connectivity with superior parietal areas 7Am and 7Pm, parts of dorsal stream processing which are implicated in visuo‐motor spatial functions (Orban et al., 2021; Rolls et al., 2022a, 2022d; Snyder et al., 1998) and coordinate transforms from egocentric eye‐based frames to allocentric world‐based frames suitable for idiothetic update of hippocampal spatial representations (Rolls, 2020, 2021b, 2022b; Snyder et al., 1998). Consistent with this, a cingulate sulcus visual area has been described that responds selectively to visual and vestibular cues to self‐motion (Smith et al., 2018), and this may be according to Figure S1 in or near 23d. Consistent with the above evidence that Group 2 is involved in navigation, area 31 which receives from POS1 and POS2 is implicated by neuroimaging in representing heading direction (Baumann & Mattingley, 2021). Consistent with the concept that navigation and executive function are performed to achieve goals, this part of the posterior cingulate and medial parietal cortex (31a, 23d, and PCV) receives from the reward‐related medial orbitofrontal cortex and pregenual anterior cingulate cortex (Figure 2; Grabenhorst & Rolls, 2011; Rolls, 2022a, 2022b; Rolls et al., 2020, 2022e).

The retrosplenial cortex RSC has similar connectivity to the PCV, 31a, and 23d, but also has connections with early visual cortical areas as shown in Figure 6. Consistent with visuo‐spatial roles for Group 2 regions, the RSC, which also receives from POS2 (Figure 2), is implicated by neuroimaging studies in representing permanent features of an environment such as landmark identity and location (Auger et al., 2012; Baumann & Mattingley, 2021; Persichetti & Dilks, 2019). The input from the reward‐related pregenual anterior cingulate cortex and medial orbitofrontal cortex to the RSC (p24, p32, 11l, Figure 2) is of interest, for it provides a pathway for navigation to be performed to reach rewards or goals (Rolls, 2022a; Rolls et al., 2022e).

An important type of navigation involves an update of location based on self‐motion, and one area in which vestibular and optic flow information is represented is macaque area 7a (Avila et al., 2019; Bremmer et al., 2000; Cullen, 2019; Wurtz & Duffy, 1992). As shown here, there is connectivity of area 7 regions with the Group 2 regions (Figure 2), which in turn connect to the hippocampal system and parahippocampal gyrus TH (PHA1‐3; Figures 3 and 8). It is proposed that these Group 2 PCC regions provide one route for optic flow and vestibular signals to reach the hippocampus in which whole‐body motion neurons are found (O'Mara et al., 1994). Some of these whole‐body motion neurons respond to linear and others to rotational motion, and some of them respond to vestibular inputs, others to visual inputs for motion, and some to both (O'Mara et al., 1994). These neurons are probably involved in navigation especially when the view details are obscured, that is, idiothetic (self‐motion updated) navigation (O'Mara et al., 1994; Rolls, 2020; Rolls, 2021b). (The rodent equivalent is probably “speed cells”; Kropff et al., 2015.) Consistent with this proposal, in macaques there are neurons in a dorsal posterior cingulate region (and to a smaller extent in the RSC) that respond to vestibular inputs (Liu et al., 2021). Additional egomotion areas in the human brain as shown by neuroimaging include intraparietal sulcus area 1 (IPS1) and V3A in the occipital region; and V6 (Sulpizio et al., 2020); and all of these regions connect to Group 3 regions DVT and ProS, which in turn connect to Group 2 PCD regions (Figure 2), which in turn connect to parietal 7 (7Pm and 7Am; Figure 3). These connectivities help to show how self‐motion information can reach hippocampal whole body motion cells (O'Mara et al., 1994) for use in idiothetic navigational updating (Rolls, 2020, 2021b; Rolls et al., 2022d).

It is therefore proposed that the group 2 PCC regions by virtue of the connectivity described here support spatial functions including navigation, and executive function, consistent with human neuroimaging and brain lesion evidence (Buckner & DiNicola, 2019; Dastjerdi et al., 2011; Ekstrom et al., 2017; Foster et al., 2012, 2013; Fox et al., 2018; Teghil et al., 2021), as considered further below.

### 4.3 Group 3, dorsal visual transitional area and ProS region

The ProS region is adjacent to V1, and the DVT area is an area posterior to most of the Posterior Cingulate Division, found just lateral to POS2 (Figures 1c, 8, and S1‐4). Importantly, the retrosplenial cortex scene area is located in the DVT region and ProS region with some extension into parieto‐occipital sulcus region 1 (POS1; Sulpizio et al., 2020). The ProS and DVT regions have connectivity with early cortical visual regions, with areas representing visual scenes (PHA1‐3 and VMV regions (Sulpizio et al., 2020)), with the parietal cortex, with hippocampal system regions (hippocampus and presubiculum), and with the premotor MCC and associated somatosensory area 5. Unlike the Group 1 regions, ProS and DVT do not have connectivity as a hub‐like region useful for episodic memory, in that they have no connectivity with the inferior temporal visual cortex (TE) or superior temporal auditory association cortex, with the dorsolateral prefrontal cortex, or with the Group 1 regions (Figure 2). Unlike Group 2 regions, the Group 3 regions have connectivity with somatosensory cortex (5), and have no connectivity with the frontal cortex. The Group 3 regions thus appear to be involved in relatively low‐level visual processing, with connectivity with the superior parietal cortex area 7 which suggests that the Group 3 regions are involved in visuo‐motor control appropriate for motor actions in visual space (Andersen, 1995; Huang & Sereno, 2018; Orban et al., 2021; Rolls et al., 2022a, 2022d; Urgen & Orban, 2021), and perhaps in the necessary spatial coordinate transforms (Rolls, 2020; Salinas & Sejnowski, 2001). Indeed, spatial coordinate transforms are also necessary for idiothetic update of spatial representations useful for hippocampal function including navigation when the spatial view is obscured (Dean & Platt, 2006; Rolls, 2020, 2021b, 2022b; Snyder et al., 1998; Vedder et al., 2017).

The DVT receives input from V6 and V6A (Figure 2). Area V6A in the macaque is a visual‐somatosensory area that occupies the posterior part of the dorsal precuneate cortex (Gamberini et al., 2020, 2021; Rolls et al., 2022d). It represents the upper limbs and is involved in the control of goal‐directed arm movements (Fattori et al., 2017). Macaque V6A hosts the so called “real‐position cells,” that is visual cells that encode spatial position in head‐based (craniotopic) coordinates not in retinotopic coordinates (Galletti et al., 1993). Area V6A is strongly connected with prestriate visual areas, with superior parietal areas, and with the premotor frontal cortex representing arm movement (Gamberini et al., 2021; Rolls et al., 2022d). Macaque V6A is divided into two subareas that together are involved in the visual and somatosensory aspects of “reach‐to‐grasp”: V6Av which is more visual and V6Ad which is more somatosensory (Gamberini et al., 2018). The human homolog of V6Av has been identified in the posterior, dorsal‐most part of precuneate cortex (Pitzalis et al., 2013), in a territory probably included in the DVT region of the HCP‐MMP1 atlas (Glasser, Coalson, et al., 2016a) and which (like macaque V6Av) is activated by optic flow (Pitzalis et al., 2013). Another part of the macaque dorsal precuneate region includes the medial portion of area PEc (Gamberini et al., 2020). Area PEc is a visual‐somatosensory area which represents both upper and lower limbs and is probably involved in locomotion and in the analysis of related optic flow (Gamberini et al., 2020; Raffi et al., 2011). Area PEc is strongly connected with part of posterior cingulate cortex 31, area 7m and retrosplenial cortex (Bakola et al., 2010). Recently, the human homolog of PEc has been identified in the dorsal‐most part of the precuneate cortex (Pitzalis et al., 2019).

DVT and ProS in Group 3 (in addition to POS1, POS2, and the RSC in Group 2) may provide a route for scene information to be represented in the VMV regions (Sulpizio et al., 2020) and thus to reach the hippocampal system and parahippocampal TH cortex PHA1‐3 (see Figure 3), and thereby to provide a key input to drive spatial view cells (Georges‐François et al., 1999; Robertson et al., 1998; Rolls, 2022b; Rolls et al., 1997, 1998; Rolls & Wirth, 2018; Tsitsiklis et al., 2020; Wirth et al., 2017) which provide a component of primate (including human) episodic memory by enabling objects, people, or rewards to be associated with their location in visual scenes (Rolls et al., 2005; Rolls & Xiang, 2005, 2006), and are likely to be useful in navigation from viewed landmark to viewed landmark too (Rolls, 2021b). This is part of a ventromedial visual cortical stream that is proposed to encode scene information to provide “where” inputs to the hippocampal spatial and memory system (Rolls et al., 2022d). In this context, the dorsal visual transitional region (DVT) and ProS region are key parts of the retrosplenial scene area (Sulpizio et al., 2020), which connect earlier visual cortex ventral stream regions to the parahippocampal scene area (or PPA) in the VMV and PHA1‐3 regions (Rolls, 2022b; Rolls et al., 2022d).

This Group 3 part of the PCC may also provide a route for vestibular and optic flow information useful in navigation to reach hippocampal whole body motion neurons, for it receives inputs from VIP and V6A in which optic flow is represented (Delle Monache et al., 2021; Duhamel et al., 1997; Sherrill et al., 2015).

### 4.4 Midcingulate cortex: 23c, 24dd, and 24dv

Because the MCC is a premotor cortical region (Vogt, 2016) that is immediately anterior to the posterior cingulate cortex, and might receive inputs from perhaps adjacent parts of the posterior cingulate cortex, the connectivity of the MCC was included in the analysis. The MCC or cingulate motor area has much connectivity to somatomotor cortical areas, and receives input from the supracallosal anterior cingulate cortex (Figures 2 and 3) which has connectivity with premotor/somatosensory regions (Rolls et al., 2022e) and is activated by aversive and non‐reward stimuli (Grabenhorst & Rolls, 2011; Rolls et al., 2020). The MCC may thus provide for aversive/non‐reward stimuli represented in the lateral orbitofrontal cortex to produce appropriate limb responses to such stimuli, including limb withdrawal, flight, and fight and more generally for action‐outcome learning (Rolls et al., 2022e). The MCC also receives from the Group 2 PCC regions implicated in executive function and navigation (Figure 7). In humans, a part of 23c termed the cingulate sulcus visual area is activated by visual self‐motion and particularly by changing heading (Smith, 2021), and it is suggested that this reflects its inputs from the Group 2 regions just posterior to it such as 23d.

### 4.5 Comparison with connections in non‐human primates

Tract‐tracing anatomical investigations of the connections of the PCC areas 23 and 31 in non‐human primates (Vogt & Laureys, 2009) generally support what is described here for humans as described next. (Evidence from rodents is not relevant, in that rodents are believed not to have PCC areas 23 and 31 [Vogt & Laureys, 2009]. Rodents have for posterior cingulate cortex only what is termed an RSC region with cytoarchitecturally defined areas 30 and 29c [Vogt & Paxinos, 2014].) The connections of the RSC in macaques have also been described (Kobayashi & Amaral, 2003, 2007; Vann et al., 2009), as follows. In macaques, posterior parietal area 7a projects to d23a, d23b, and d23c (and provides spatial information including head position and eye position which can be referenced to the world (Rolls, 2020; Snyder et al., 1998)). Medial parietal 7m projects to 31 and 23d (and to MCC 23c), and in humans 7Pm projects strongly to Group 2 spatial regions (Figure 2). Auditory areas in the superior temporal gyrus in macaques project to d23 and v23, providing auditory “what” (ventral stream) information into these Group 1 areas, somewhat similarly to the situation in humans though in humans visual “what” information from TE1 also reaches the Group 1 areas (Figure 2). The frontal pole cortex area 10 and the orbitofrontal cortex area 11 in macaques have projections to the RSC (Kobayashi & Amaral, 2003), supporting and validating what is described here in humans for connectivity from regions p10p and OFC to Group 2 PCD regions (Figure 2). The macaque connectivity of the PCC with the presubiculum, subiculum, and TH (Kobayashi & Amaral, 2003, 2007), also supports what is described here for humans. The monkey does not have inferior parietal areas 39 (angular gyrus, anterior PG regions, relating on the left to dyslexia and agraphia, and on the right to body image) and 40 (marginal gyrus, PF regions, relating on the left to phonology, and also part of the mirror neuron system) and also involved in memory (Caspers et al., 2008; Coslett & Schwartz, 2018; Davis et al., 2018; Glasser, Coalson, et al., 2016a; Papagno, 2018; Rizzolatti & Rozzi, 2018; Ronchi et al., 2018; Vogt & Laureys, 2009), so there is no equivalent in macaques of the strong connectivity in humans from the memory‐related (Davis et al., 2018; Papagno, 2018) inferior parietal areas PGi and PGs (Rolls et al., 2022a) to the Group 1 PCC regions. The tract‐tracing studies in non‐human primates thus provide support and validation for many of the findings described here, though the present investigation goes beyond these by providing evidence on humans, in whom many of the brain regions involved have developed greatly, and by providing evidence of the physiological strength of the connectivities.

Although the new findings on the human PCC connectivity described here are generally supported by macaque neuroanatomy (Kobayashi & Amaral, 2003, 2007; Vogt & Laureys, 2009), the present findings in humans go beyond that because there is considerable development of the human compared with the macaque brain, including of areas such as the inferior parietal cortex, orbitofrontal cortex, dorsolateral prefrontal cortex, and language‐related areas such as the cortex in the STS and the inferior frontal gyrus (Pandya et al., 2015; Passingham, 2021; Rolls, 2021a; Rolls et al., 2022a, 2022b, 2022e). The macaque anatomy does provide important evidence on the cortical layers from which projections originate and where they terminate (Kobayashi & Amaral, 2003, 2007; Markov et al., 2013, 2014) which is important in understanding cortical function as it provides insight into the operation of bottom‐up versus top‐down cortical processing and how cortical systems operate in memory (Rolls, 2016). On the other hand that is complemented by the effective connectivity described here which provides evidence on the physiological strengths and their directions between cortical regions in humans, which for similar reasons is important in understanding brain computations (Rolls, 2016, 2021a).

### 4.6 Anatomical–functional synthesis

Here the aim is to synthesize the connectivity information and combine it with evidence on the functions of some of the brain regions to produce working hypotheses on the anatomico‐functional organization of the posterior cingulate cortex / RSC and MCC. This is done with reference to the summaries in Figures 7 and 8, and takes into account previous analyses of the connectivity of the posterior cingulate cortex (Baker et al., 2018; Glasser, Coalson, et al., 2016a; Khalsa et al., 2014; Vogt & Laureys, 2009). The article by Baker et al. (2018) is especially helpful in summarizing analyses of functional connectivity and diffusion tractography using the HCP‐MMP atlas and summarizing some of the task‐related fMRI results for each brain region (Glasser, Coalson, et al., 2016a), though the present analyses extend this by providing effective connectivity analyses, new tractography analyses, and presenting the results in quantitative matrix format so that all the connectivity can be evaluated.

#### 4.6.1 Group 1 (31pd, 31pv, d23ab, v23ab, and 7m)

We start with the postero‐ventral Posterior Cingulate Division regions, Group 1 (31pd, 31pv, 7m, d23ab, and v23ab), which have quite similar effective connectivity with each other (Figure S4) and which form a distinct connectivity‐based community. We propose that Group 1 provides links from many high‐order cortical areas into the hippocampal memory system, where all of these types of input can be associated together in the hippocampal CA3 network to implement episodic memory storage (Figures 7 and 8; Rolls, 2018, 2021a; Rolls & Treves, 1994; Treves & Rolls, 1994). The weaker return pathways from the hippocampus to the parahippocampal cortex and thereby to neocortical regions provide for memory retrieval (Figures 7 and 8; Rolls, 2021a, 2022a; Treves & Rolls, 1994). These Group 1 connectivities provide a route to the hippocampus for key elements for the hippocampal memory system for inputs about “what” (provided by the visual ventral stream TE and auditory STS regions); “when” (provided by sequence and planning inputs from area 10p, and also by time cells that may be computed in the lateral entorhinal to hippocampus system [Eichenbaum, 2017; Rolls & Mills, 2019; Tsao et al., 2018]); and reward/emotion value provided by the pregenual anterior cingulate cortex (Grabenhorst & Rolls, 2011) to the Group 1 regions (Figure 2; Rolls, 2022a; Rolls et al., 2022e). Further, the posterior cingulate cortex Group 1 regions provide a key route from multimodal “what” parietal cortex region PGi to the hippocampal system (Rolls et al., 2022a). As shown in Figures 7 and 8, the Group 1 part of the posterior cingulate and medial parietal cortex has these inputs, and then has effective connectivity directed to the hippocampal system (Rolls et al., 2022f; complemented by anatomical connections [Huang et al., 2021] and functional connectivity [Ma et al., 2022]), with weaker return backprojection pathways as required for recall that does not dominate bottom‐up inputs (Rolls, 2016; Rolls, 2021a; Treves & Rolls, 1994).

The evidence presented here thus is that the posterior, Group 1, parts of the posterior cingulate and medial parietal cortex provide important links from many high‐order cortical areas into the hippocampal memory system. Consistent with this, a ventral (/posterior) subpart of the PCC is preferentially recruited during episodic remembering and imagining the future (DiNicola et al., 2020) including autobiographical memory (Davis et al., 2018; Papagno, 2018; Summerfield et al., 2009). It is further proposed that the posterior cingulate and medial parietal cortex provide a route for inferior parietal cortex area PG regions (Rolls et al., 2022a; angular gyrus, BA39), as well as temporal pole and STS regions (Rolls et al., 2022b, 2022d; see Figure 2) to gain access to the hippocampal memory system. These areas are implicated in semantic representations (Bonner & Price, 2013; Price et al., 2015; Rolls et al., 2022b), allowing them access to the hippocampal memory system. Consistent with the proposed posterior cingulate and medial parietal cortex reward pathway to the hippocampus from the pregenual anterior cingulate cortex (Rolls et al., 2022e), part of the posterior cingulate cortex has activations related to value, with responses in macaques related for example to risky decisions (McCoy & Platt, 2005; Pearson et al., 2011). Baker et al. (2018) agree that these Group 1 regions have functions related to memory, including episodic memory, semantic memory, and working memory.

Thus “what,” “when,” reward, and semantic (including schema) information have routes to the hippocampus via the Group 1 PCC regions. Further, given that the Group 1 regions receive from the ends of each of many different processing streams (e.g., for vision from TE, for hearing, etc., from the STS, somatosensory/visual from parietal, and reward/emotional value from the anterior cingulate cortex; Rolls et al., 2022e), it is suggested that the Group 1 regions contribute to computing multimodal semantic representations of objects consistent with their connections to a language network that includes the ventral parts of the cortex in the superior temporal sulcus shown in Figures 2 and 3 (Rolls et al., 2022b), which are then provided as the 'what' input to the hippocampal memory system.

#### 4.6.2 Group 2 (23d, 31a, PCV, and RSC, POS2, and POS1)

We propose that the Group 2 and 3 regions contribute to providing “where” information for the hippocampal episodic memory, as described next.

Taking the Group 2 areas POS1, POS2, and RSC, these may be involved in introducing spatial scene/spatial view information into the hippocampal system as a “where” component of episodic memory (Figures 7 and 8b) with connectivity with visual cortical areas (Figure 2). This part of the PCD, especially the RSC which receives from POS2, thus may provide a route for scene information to reach the hippocampus to provide a key input to drive spatial view cells (Georges‐François et al., 1999; Robertson et al., 1998; Rolls, 2022b; Rolls et al., 1997, 1998, 2022d; Rolls & Wirth, 2018; Tsitsiklis et al., 2020; Wirth et al., 2017) which provide a component of primate (including human) episodic memory by enabling objects, people, or rewards to be associated with their location in visual scenes (Rolls, 2022a; Rolls et al., 2005; Rolls & Xiang, 2005, 2006), and are likely to be useful in navigation from viewed landmark to viewed landmark too (Rolls, 2021b).

The connectivity analyses described here help to advance understanding of the connectional pathways providing for scene‐related information to reach hippocampal spatial view cells, as considered next. There are several scene areas in the human brain, including the occipital place (or scene) area (OPA), the retrosplenial cortex scene area, and the parahippocampal scene area (PPA; Epstein, 2005, 2008; Epstein & Baker, 2019; Epstein & Julian, 2013; Epstein & Kanwisher, 1998; Kamps et al., 2016; Natu et al., 2021; Sulpizio et al., 2020). (Use of the abbreviation PSA instead of PPA is preferred for these regions, because the neuroimaging evidence is consistent with scene responsiveness, not the place where the participant being imaged is located; Rolls, 2022b.) With respect to the HCP‐MMP atlas (Figures 8 and S1; Glasser, Coalson, et al., 2016a; Huang et al., 2022), an investigation using this atlas has provided evidence that the occipital place area OPA is located in V3CD and nearby regions including LO1; that the retrosplenial cortex scene area is located in the dorsal visual transitional region (DVT) and ProS region with some extension into parieto‐occipital sulcus region 1 (POS1); and that the PPA (i.e., PSA) is located in the ventromedial visual areas (VMV1‐3) extending anteriorly into the parahippocampal gyrus PHA1‐3 (which correspond to parahippocampal TH; Rolls, 2022b; Sulpizio et al., 2020). Figures 2 and 3 show for example that DVT, ProS, and POS1 (the retrosplenial cortex scene area) have effective connectivity to the Group 2 regions, and to the VMV regions that are part of the PSA (Rolls et al., 2022d). The VMV areas then have strong effective connectivity to parahippocampal TH (PHA1‐3; Figure 2), which in turn has strong effective connectivity to the hippocampus (Rolls et al., 2022f; supported by functional connectivity [Ma et al., 2022] and diffusion tractography [Huang et al., 2021]), showing how scene information can reach hippocampal cells that respond to scenes (Georges‐François et al., 1999; Mao et al., 2021; Robertson et al., 1998; Rolls, 2021c, 2022b; Rolls et al., 1997, 1998, 2022d; Rolls & Wirth, 2018; Tsitsiklis et al., 2020; Wirth et al., 2017).

The dorsal/anterior PCC areas in Group 2 (23d, 31a, PCV, and RSC, Figure 1b), it is suggested based on the literature summarized above, also provide access to the hippocampal system for spatial inputs from superior parietal areas 7Am and 7Pm, which are implicated in visuo‐motor spatial functions and tool use (Battaglia‐Mayer & Caminiti, 2018; Orban et al., 2021; Snyder et al., 1998) and coordinate transforms from egocentric eye‐based frames to allocentric world‐based frames suitable for idiothetic update of hippocampal spatial representations (Rolls, 2020, 2021b; Snyder et al., 1998). The precuneus visual region PCV in the medial parietal cortex is especially related to regions involved in these coordinate transform processes by its extensive visual cortex connectivity including with parietal area 7 regions and PGp, and it has connectivity to the hippocampus and presubiculum (Figures 2, 3, 4, 5, 6).

Baker et al. (2018) agree that these Group 2 regions have functions related to spatial functions including those involved in navigation and episodic memory.

The Group 2 regions are also implicated in executive function by their strong inputs from frontal pole region p10p implicated in planning and sequencing (Gilbert & Burgess, 2008; Shallice & Burgess, 1996; Shallice & Cipolotti, 2018), by their inputs from the reward‐related medial orbitofrontal cortex 11l and pregenual anterior cingulate cortex (Rolls et al., 2022e) and punishment and non‐reward related supracallosal anterior cingulate cortex (signified with a pr standing for prime or ′), and by the outputs to the midcingulate premotor cortex (Figures 2, 3, 4, 5, 6, 7 and 8b). Consistent with this, the dorsal (/anterior) part of the posterior cingulate cortex is activated during some executive function tasks such as visual search and mental arithmetic (Buckner & DiNicola, 2019; Dastjerdi et al., 2011; Foster et al., 2012; Foster et al., 2013; Fox et al., 2018). The Group 2 regions are strategically located just posterior to the MCC (Figure 8b), thereby minimizing wiring length in providing a route for Group 2 regions to influence behavior by the MCC and thereby other premotor cortical areas. The Group 2 regions are also strategically located in that they receive from early visual cortical areas and visual medial parietal area 7, as shown in Figure 8b. These points provide a foundation for understanding the topology of the posterior cingulate cortex.

Baker et al. (2018) agree that these Group 2 regions have functions related to memory, including episodic memory, semantic memory, and working memory.

#### 4.6.3 Group 3 (ProS and DVT)

The regions in Group 3, the ProS region and the dorsal visual transitional region DVT (Figure 1c), are the main part of the retrosplenial scene area (Sulpizio et al., 2020), receive from early visual cortical areas, connect to the ventromedial visual area (VMV) which connect to medial parahippocampal PHA1‐3 in which visual scenes are represented (Sulpizio et al., 2020), which in turn have outputs to the hippocampal system and midcingulate motor cortex (Figures 2, 3, 4, 5, 6; Rolls et al., 2022d). The Group 3 regions thus are part of a ventromedial visual cortical stream that provides a “where” component of episodic memory (Figure 7; Rolls, 2022b; Rolls et al., 2022d). PRoS and DVT, where the retrosplenial scene area is located (Sulpizio et al., 2020), are posterior to region RSC. The parahippocampal scene area is located in medial parahippocampal gyrus regions PHA1‐3, VMV1‐3, and VVC (Sulpizio et al., 2020). The occipital scene area is located in V3CD adjoining V4 (Sulpizio et al., 2020; see for diagram Rolls, 2022b). The Group 3 regions also receive from parietal cortex including the superior parietal cortex involved in visuo‐spatial responses (Figures 2, 3, 4, 5, 6). It is proposed that these parts of the PCC division are also involved in visuo‐motor functions including the coordinate transforms necessary for actions to visual stimuli (Salinas & Sejnowski, 2001), and for providing allocentric coordinates for idiothetic signals to the hippocampal system useful for navigation in the dark or when the view is obscured (Rolls, 2020, 2021b; Figure 7). The Group 3 regions are in part earlier in visual processing than Group 2 regions, and indeed provide Group 2 regions with some of their inputs as shown in Figures 2 and 3. Baker et al. (2018) comment that the ProS area is thought “to have a transitional function between the early visual cortex and posterior cingulate association cortex like DVT.” Consistent with the above, Baker et al. (2018) also comment that the dorsal visual transitional area is a newly defined region in the HCP‐MMP atlas and is “functionally connected to the dorsal stream visual cortex, which perceives where stimuli are located, as well as the superior posterior parietal cortex, which plays an important role in planned movements.”

In summary, DVT and ProS are implicated as part of the retrosplenial scene area in linking earlier visual cortical regions with ventromedial visual VMV regions and the PHA1‐3 which are the parahippocampal scene area with the hippocampus to provide a ventromedial visual “where” stream to the hippocampus for building feature‐based scene representations (Rolls, 2022b; Rolls et al., 2022d). In addition, DVT with its connectivity with parietal area 7 regions (7Am, 7Pm, and 7PL; Figure 2) and PGp is implicated in the coordinate transforms that enable spatial view cells in cortical scene areas such as the parahippocampal gyrus to be updated by self‐motion inputs (Robertson et al., 1998; Rolls et al., 2022d).

#### 4.6.4 Midcingulate cortex (23c, 24dd, and 24dv)

With respect to the MCC, sometimes termed the cingulate motor area (Vogt, 2016), it is of interest that this provides an output for Group 2 posterior cingulate and medial parietal region 7m, and has in addition very different effective connectivity to the adjoining posterior cingulate cortex (Figures 2, 3, and 7). The hypothesis is that the midcingulate region provides a route for the reward‐related representations in the medial orbitofrontal cortex and aversive and non‐reward representations in the lateral orbitofrontal cortex (Grabenhorst & Rolls, 2011), connecting via the pregenual and then supracallosal anterior cingulate cortex to the MCC, to produce limb responses via somato‐premotor cortical areas such as areas 6 and 5 and more generally to provide an output route for action‐outcome learning (Figure 7; Rolls, 2022a; Rolls et al., 2022e). Limb responses of limb withdrawal, or flight, or fight, are appropriate behaviors to especially aversive/unpleasant stimuli (Rolls et al., 2022e) which are represented in the lateral orbitofrontal cortex and supracallosal (supragenual) anterior cingulate cortex (Grabenhorst & Rolls, 2011; Rolls, 2019a; Rolls, 2019c). The effective connectivities shown in Figure 7 for the MCC support this hypothesis, which is further based on the effective connectivities shown in Figures 2 and 3. More generally, the MCC can be seen with its connectivity described here as connecting anterior cingulate cortex regions involved in action‐outcome learning to action output premotor cortical regions (Noonan et al., 2011; Rolls, 2022a; Rolls et al., 2022e; Rushworth et al., 2012).

### 4.7 Effective connectivity, functional connectivity, and diffusion tractography

These different measures complement each other in the research described here.

The effective connectivity algorithm used here is a non‐linear algorithm, and retains only effective connectivities that enable the effective connectivity matrix to maximize the correlation of the functional connectivity at time *t* and *t* + *tau* that it generates by simulation with the empirical functional connectivity measured at time *t* and *t* + *tau*. The algorithm, therefore, leaves many links in the effective connectivity matrix at zero. Because of the measurements at time *t* and the delayed *t* + *tau*, the strength of the connectivity can be measured in each direction. For some links, the effective connectivity can be zero in one direction and strong in the reverse direction, though in many cases, given the computational design of the neocortex (Rolls, 2016; Rolls, 2021a), there is often some connectivity in both directions. Effective connectivity can be described as measuring causal effects, as it utilizes time delays (Rolls, 2021d). One point to consider is the extent to which the Hopf effective connectivity algorithm when applied to the brain provides evidence that is selective for one link between two brain regions. If the system was linear and consisted of a simple series of connected stages, then the effective connectivity would be the same for all stages. But in practice, the brain is a non‐linear system, and each stage has many inputs from different brain regions and many outputs to different brain regions, so the effective connectivity measured between any pair of brain regions may reflect mainly the effective connectivity between that pair of brain regions. In practice, the effective connectivity measured between one pair of brain regions is relatively selective for that stage, with evidence for this provided elsewhere (Rolls et al., 2022d).

The functional connectivity (Figure 5) is in contrast a linear measure (the Pearson correlation across time between the BOLD signals in two brain regions) and can provide evidence that may relate to interactions between brain regions, and indirect effects relating to common input, while providing no evidence about causal direction‐specific effects. A high‐functional connectivity may in this scenario thus reflect strong physiological interactions between areas, and provides a different type of evidence for effective connectivity. The effective connectivity is non‐linearly related to the functional connectivity, with effective connectivities being identified (i.e., >0) only for the links with relatively high functional connectivity. The functional connectivities range from close to 1.0 to −0.33 and with a threshold of 0.4 reveal somewhat more links than the effective connectivity, partly perhaps because they can reflect common input to two regions rather than causal connectivity between regions, and partly because the threshold has been set to reveal effects known in the literature but not reflected in the effective connectivity. The functional connectivities are useful as a check on the effective connectivities, but of course do not measure causal effects.

The diffusion tractography (Figure 6) provides no evidence on the direction or causality of connections, and is useful as it can provide some evidence on what in the effective connectivity may reflect a direct connection, and what does not. However, limitations of the diffusion tractography are that it cannot follow streamlines within the gray matter so the exact site of termination is not perfectly provided; and the tractography does not follow long connections well, with for example almost none of the contralateral connectivity shown with tractography that is revealed by the effective connectivity in Figures S2 and S3; and may thus overemphasize connections between close cortical regions. Nevertheless, the diffusion tractography is a useful complement to the effective connectivity, especially where it provides evidence where an effective connectivity link may be mediated by a direct connection. On the other hand, the effective connectivity and functional connectivity are useful complements to the tractography by helping to exclude false positives in the tract‐following in the tractography, as has been examined for the human hippocampal connectome (Huang et al., 2021; Ma et al., 2022; Rolls et al., 2022f).

## 5 CONCLUSIONS

The effective connectivity analyses described here, complemented by functional connectivity and diffusion tractography in the same HCP participants (Glasser, Smith, et al., 2016b), and the use of the HCP‐MMP1 (Glasser, Coalson, et al., 2016a)/HCPex (Huang et al., 2022) atlases, provide unprecedented insight into the connectivity of the human posterior cingulate cortex and related RSC and medial parietal regions in the Posterior Cingulate Division of the HCP‐MMP1 atlas, and provide a framework that should be very useful when it is combined in future with neuroimaging activation studies using the same atlases. Already the main groupings evident in the connectivity of the posterior cingulate cortex, RSC and medial parietal 7m have provided new insights into the functions of the different groups in memory, and in spatial functions relevant to memory and navigation. A feature of the connectivity of the Posterior Cingulate Division is its close relation to inferior temporal lobe visual cortical processing areas, at the top of the visual stream hierarchy for the Group 1 regions (likely to provide invariant representations of objects and people; Rolls, 2021c), and to spatially relevant visual information for the Group 2 and Group 3 regions from lower in visual sensory processing from areas relating to the parahippocampal TH region in which information about spatial scenes is represented (Epstein & Baker, 2019; Epstein & Julian, 2013; Natu et al., 2021; Sulpizio et al., 2020). The human Posterior Cingulate Division can thus be seen to provide important components of inputs to the hippocampus for episodic memory, namely visual “what” representations of objects and people (Rolls et al., 2005; Sliwa et al., 2016) and visual “where” representations by spatial view neurons (Georges‐François et al., 1999; Robertson et al., 1998; Rolls, 2022b; Rolls et al., 1997, 1998; Rolls & Wirth, 2018; Rolls & Xiang, 2006; Wirth et al., 2017) that can be combined by single neurons during rapid episodic learning of “what”–“where” representations (Rolls et al., 2005; Rolls & Xiang, 2006). The Posterior Cingulate Division goes beyond that, by also receiving from reward‐related (hence emotion‐related) brain regions such as the pregenual anterior cingulate cortex and vmPFC (Figure 8), which provide important parts of episodic memories (Rolls, 2022a) and which can be associated rapidly with objects by hippocampal neurons (Rolls & Xiang, 2005, 2006). The emphasis on visual inputs about locations in viewed space by the primate (including human; Tsitsiklis et al., 2020) hippocampus (Rolls, 2022b) goes beyond what is mainly emphasized in the rodent hippocampus which is about the place where the rodent is located (Hartley et al., 2014; O'Keefe, 1979; O'Keefe & Dostrovsky, 1971), and indeed the rodent is thought not to have the posterior cingulate cortex that consists of Brodmann areas 23 and 31 (Vogt, 2009; Vogt & Laureys, 2009).

## FUNDING INFORMATION

The research was supported by the following grants to Professor J. Feng: National Key R&D Program of China (No. 2019YFA0709502); 111 Project (No. B18015); Shanghai Municipal Science and Technology Major Project, ZJLab, and Shanghai Center for Brain Science and Brain‐Inspired Technology (No. 2018SHZDZX01); and National Key R&D Program of China (No. 2018YFC1312904). G.D. is supported by a Spanish National Research Project funded by the Spanish Ministry of Science, Innovation and Universities (MCIU), State Research Agency (AEI) (ref. PID2019‐105772GB‐I00 MCIU AEI); HBP SGA3 Human Brain Project Specific Grant Agreement 3 (grant agreement no. 945539), funded by the EU H2020 FET Flagship programme; SGR Research Support Group Support (ref. 2017 SGR 1545), funded by the Catalan Agency for Management of University and Research Grants (AGAUR); Neurotwin Digital twins for model‐driven non‐invasive electrical brain stimulation (grant agreement ID: 101017716) funded by the EU H2020 FET Proactive Programme; euSNN European School of Network Neuroscience (grant agreement ID: 860563) funded by the EU H2020 MSCA‐ITN Innovative Training Networks; CECH The Emerging Human Brain Cluster (Id. 001‐P‐001682) within the framework of the European Research Development Fund Operational Program of Catalonia 2014–2020; Brain‐Connects: Brain Connectivity during Stroke Recovery and Rehabilitation (id. 201725.33) funded by the Fundacio La Marato TV3; Corticity, FLAG ERA JTC 2017, (ref. PCI2018‐092891) funded by the Spanish Ministry of Science, Innovation and Universities (MCIU), State Research Agency (AEI). The funding agencies played no role in the study design; in the collection, analysis, and interpretation of data; in the writing of the report; or in the decision to submit the article for publication.

## CONFLICT OF INTEREST

The authors declare no conflicts of interest.

## Supporting information


**Appendix S1** Supporting Information

## Data Availability

The data are available at the HCP website http://www.humanconnectome.org/. Code for the Hopf effective connectivity algorithm is available at https://github.com/decolab/Effective-Connectivity--Hopf.
