# Supplementary material for: The human posterior cingulate, retrosplenial, and medial parietal cortex effective connectome, and implications for memory and navigation
Source: Hum Brain Mapp. 2022 Sep 30;44(2):629–55. doi: 10.1002/hbm.26089 (PMC9842927; doi:10.1002/hbm.26089)
Supplement: Supplementary file 1 — Appendix S1 Supporting Information [file HBM-44-629-s001.docx]

**The human posterior cingulate, retrosplenial and medial parietal cortex effective connectome, and implications for memory and navigation**

**Supplementary Material**

Edmund T. Rolls^1, 2, 7*^, Sylvia Wirth^3^, Gustavo Deco^4,5^, Chu-Chung Huang^6^, Jianfeng Feng^2,7^

1. Oxford Centre for Computational Neuroscience, Oxford, UK
2. Department of Computer Science, University of Warwick, Coventry, CV4 7AL, UK
3. Institut des Sciences Cognitives Marc Jeannerod, UMR 5229, CNRS and University of Lyon, Bron, France
4. Center for Brain and Cognition, Computational Neuroscience Group, Department of Information and Communication Technologies, Universitat Pompeu Fabra, Roc Boronat 138, Barcelona, 08018, Spain; Brain and Cognition, Pompeu Fabra University, Barcelona, 08018, Spain.
5. Institució Catalana de la Recerca i Estudis Avançats (ICREA), Universitat Pompeu Fabra, Passeig Lluís Companys 23, Barcelona, 08010, Spain.
6. Shanghai Key Laboratory of Brain Functional Genomics (Ministry of Education), School of Psychology and Cognitive Science, East China Normal University, Shanghai 200602, China
7. Institute of Science and Technology for Brain Inspired Intelligence, Fudan University, Shanghai 200403, China; Key Laboratory of Computational Neuroscience and Brain Inspired Intelligence, Fudan University, Ministry of Education, Shanghai 200433, China; Fudan ISTBI—ZJNU Algorithm Centre for Brain-inspired Intelligence, Zhejiang Normal University, Jinhua, China.

*Corresponding author information:

Professor Edmund T. Rolls,

Department of Computer Science, University of Warwick, Coventry CV4 7AL, UK. Email: [Edmund.Rolls@oxcns.org](mailto:Edmund.Rolls@oxcns.org)

URL: [https://www.oxcns.org](https://www.oxcns.org/)

https://orcid.org/0000-0003-3025-1292

**Modified version of the HCP-MMP1 atlas**

The atlas used to define brain regions was the HCP-MMP1 multimodal (based on cortical myelin, thickness, functional connectivity and task-related activations) atlas with 360 cortical regions (Glasser, et al., 2016), converted into volumetric space and modified as follows as described in detail elsewhere to produce the extended HCP atlas, HCPex (Huang, et al., 2022). First, the hippocampus and subiculum were defined as separate regions, using the template provided by Winterburn et al (2013). In our list of areas, shown in Table S1, the new hippocampal region was allocated into the hippocampal slot in the HCP list. The subiculum appears later in the list as a new area. Other new areas in the HCPex atlas (Huang, et al., 2022) include the thalamus, caudate, putamen, globus pallidus, amygdala, nucleus accumbens, substantia nigra, septum and basal forebrain nuclei of Meynert. The subcortical regions are shown in Table 2.

In the HCP-MMP1 atlas, each region has its RegionID, which we show in Table S1. Detailed information about the regions is available in the Supplementary Material File NIHMS68870-supplement-Neuroanatomical_Supplementary_Results.pdf provided by Glasser et al (2016). In that Supplementary Material file, a grouping of the regions is suggested based on geographic proximity and functional similarities, and this grouping is shown in the column labelled CortexID in Table S1. That has led to a different ordering of the regions, which we show in Table S1, with the original regionIDs from the HCP atlas shown in the column headed ‘regionID’. This reordered version of the HCP-MMP1 atlas is described by Dr Dianne Patterson of the University of Arizona at <https://neuroimaging-core-docs.readthedocs.io/en/latest/pages/atlases.html>, where the following supporting files used to help generate Table S1 are available: HCP-MMP1_UniqueRegionList.csv and Glasser_2016_Table.xlsx. We made file HCPMMP_CortexID_Ordering.xlsx from this, and this is available from the present authors. The connectivity matrices shown in the present paper used the ordering shown in Table S1.

Table S1. Regions defined in the modified Human Connectome Project atlas (Glasser, et al., 2016). L=left hemisphere, R=right. The column ‘Reordered region ID’ is that used in Figs. 2-6, and is a reordering of that based on suggestions in the Supplementary Information of Glasser et al (2016). In that Supplementary Information, the 360 regions are grouped based on geographic proximity and functional similarities, which was reorganized and provided by Dr Dianne Patterson of the University of Arizona at [https://neuroimaging-core-docs.readthedocs.io/en/latest/pages/atlases.html with the HCP-MMP1_UniqueRegionList.csv](https://neuroimaging-core-docs.readthedocs.io/en/latest/pages/atlases.html%20with%20the%20HCP-MMP1_UniqueRegionList.csv) and is shown in the column labelled CortexID in Table S1. The volumes are in mm^3^. This modified atlas with the reordering is described elsewhere (Huang, et al., 2022). The subiculum was an additional area to the 180 shown here.

| **Reordered**  **ID (L, R)** | **Region** | **RegionLongName** | **Cortical Division** | **Cortex**  **ID** | **Original**  **ID** | **Voxel numbers (1mm^3^) (L,R)** |
| --- | --- | --- | --- | --- | --- | --- |
| 1, 181 | V1 | Primary_Visual_Cortex | Primary_Visual | 1 | 1 | 13812, 13406 |
| 2, 182 | V2 | Second_Visual_Area | Early_Visual | 2 | 4 | 9515, 9420 |
| 3, 183 | V3 | Third_Visual_Area | Early_Visual | 2 | 5 | 7106, 7481 |
| 4, 184 | V4 | Fourth_Visual_Area | Early_Visual | 2 | 6 | 4782, 4537 |
| 5, 185 | IPS1 | IntraParietal_Sulcus_Area_1 | Dorsal_Stream_Visual | 3 | 17 | 1751, 1750 |
| 6, 186 | V3A | Area_V3A | Dorsal_Stream_Visual | 3 | 13 | 2191, 2212 |
| 7, 187 | V3B | Area_V3B | Dorsal_Stream_Visual | 3 | 19 | 639, 731 |
| 8, 188 | V6 | Sixth_Visual_Area | Dorsal_Stream_Visual | 3 | 3 | 1402, 1559 |
| 9, 189 | V6A | Area_V6A | Dorsal_Stream_Visual | 3 | 152 | 904, 734 |
| 10, 190 | V7 | Seventh_Visual_Area | Dorsal_Stream_Visual | 3 | 16 | 1005, 1041 |
| 11, 191 | FFC | Fusiform_Face_Complex | Ventral_Stream_Visual | 4 | 18 | 3848, 4402 |
| 12, 192 | PIT | Posterior_InferoTemporal_complex | Ventral_Stream_Visual | 4 | 22 | 1392, 1386 |
| 13, 193 | V8 | Eighth_Visual_Area | Ventral_Stream_Visual | 4 | 7 | 1361, 1175 |
| 14, 194 | VMV1 | VentroMedial_Visual_Area_1 | Ventral_Stream_Visual | 4 | 153 | 939, 1219 |
| 15, 195 | VMV2 | VentroMedial_Visual_Area_2 | Ventral_Stream_Visual | 4 | 160 | 639, 923 |
| 16, 196 | VMV3 | VentroMedial_Visual_Area_3 | Ventral_Stream_Visual | 4 | 154 | 941, 1242 |
| 17, 197 | VVC | Ventral_Visual_Complex | Ventral_Stream_Visual | 4 | 163 | 2487, 2753 |
| 18, 198 | FST | Area_FST | MT+_Complex | 5 | 157 | 1324, 1683 |
| 19, 199 | LO1 | Area_Lateral_Occipital_1 | MT+_Complex | 5 | 20 | 619, 909 |
| 20, 200 | LO2 | Area_Lateral_Occipital_2 | MT+_Complex | 5 | 21 | 1179, 1062 |
| 21, 201 | LO3 | Area_Lateral_Occipital_3 | MT+_Complex | 5 | 159 | 438, 915 |
| 22, 202 | MST | Medial_Superior_Temporal_Area | MT+_Complex | 5 | 2 | 794, 1036 |
| 23, 203 | MT | Middle_Temporal_Area | MT+_Complex | 5 | 23 | 620, 1005 |
| 24, 204 | PH | Area_PH | MT+_Complex | 5 | 138 | 3453, 3205 |
| 25, 205 | V3CD | Area_V3CD | MT+_Complex | 5 | 158 | 876, 1222 |
| 26, 206 | V4t | Area_V4t | MT+_Complex | 5 | 156 | 1037, 1249 |
| 27, 207 | 1 | Area_1 | SomaSens_Motor | 6 | 51 | 6590, 5925 |
| 28, 208 | 2 | Area_2 | SomaSens_Motor | 6 | 52 | 4278, 4727 |
| 29, 209 | 3a | Area_3a | SomaSens_Motor | 6 | 53 | 2247, 2286 |
| 30, 210 | 3b | Primary_Sensory_Cortex | SomaSens_Motor | 6 | 9 | 5451, 4350 |
| 31, 211 | 4 | Primary_Motor_Cortex | SomaSens_Motor | 6 | 8 | 10776, 10254 |
| 32, 212 | 23c | Area_23c | ParaCentral_MidCing | 7 | 38 | 2259, 2498 |
| 33, 213 | 24dd | Dorsal_Area_24d | ParaCentral_MidCing | 7 | 40 | 2665, 2820 |
| 34, 214 | 24dv | Ventral_Area_24d | ParaCentral_MidCing | 7 | 41 | 1076, 1349 |
| 35, 215 | 5L | Area_5L | ParaCentral_MidCing | 7 | 39 | 2249, 2327 |
| 36, 216 | 5m | Area_5m | ParaCentral_MidCing | 7 | 36 | 1483, 2079 |
| 37, 217 | 5mv | Area_5m_ventral | ParaCentral_MidCing | 7 | 37 | 1651, 1996 |
| 38, 218 | 6ma | Area_6m_anterior | ParaCentral_MidCing | 7 | 44 | 3941, 4251 |
| 39, 219 | 6mp | Area_6mp | ParaCentral_MidCing | 7 | 55 | 3701, 3105 |
| 40, 220 | SCEF | Supplementary_and_Cingulate_Eye_Field | ParaCentral_MidCing | 7 | 43 | 3500, 3371 |
| 41, 221 | 55b | Area_55b | Premotor | 8 | 12 | 2422, 1537 |
| 42, 222 | 6a | Area_6_anterior | Premotor | 8 | 96 | 4233, 3752 |
| 43, 223 | 6d | Dorsal_area_6 | Premotor | 8 | 54 | 2916, 2909 |
| 44, 224 | 6r | Rostral_Area_6 | Premotor | 8 | 78 | 3029, 3981 |
| 45, 225 | 6v | Ventral_Area_6 | Premotor | 8 | 56 | 2075, 2516 |
| 46, 226 | FEF | Frontal_Eye_Fields | Premotor | 8 | 10 | 1787, 1889 |
| 47, 227 | PEF | Premotor_Eye_Field | Premotor | 8 | 11 | 1006, 1258 |
| 48, 228 | 43 | Area_43 | Posterior_Opercular | 9 | 99 | 1889, 1678 |
| 49, 229 | FOP1 | Frontal_Opercular_Area_1 | Posterior_Opercular | 9 | 113 | 879, 932 |
| 50, 230 | OP1 | Area_OP1-SII | Posterior_Opercular | 9 | 101 | 1275, 1072 |
| 51, 231 | OP2-3 | Area_OP2-3-VS | Posterior_Opercular | 9 | 102 | 943, 792 |
| 52, 232 | OP4 | Area_OP4-PV | Posterior_Opercular | 9 | 100 | 2332, 2409 |
| 53, 233 | 52 | Area_52 | Early_Auditory | 10 | 103 | 725, 580 |
| 54, 234 | A1 | Primary_Auditory_Cortex | Early_Auditory | 10 | 24 | 1023, 796 |
| 55, 235 | LBelt | Lateral_Belt_Complex | Early_Auditory | 10 | 174 | 820, 901 |
| 56, 236 | MBelt | Medial_Belt_Complex | Early_Auditory | 10 | 173 | 1242, 1236 |
| 57, 237 | PBelt | ParaBelt_Complex | Early_Auditory | 10 | 124 | 1719, 1439 |
| 58, 238 | PFcm | Area_PFcm | Early_Auditory | 10 | 105 | 1486, 1485 |
| 59, 239 | RI | RetroInsular_Cortex | Early_Auditory | 10 | 104 | 1149, 1334 |
| 60, 240 | A4 | Auditory_4_Complex | Auditory_Association | 11 | 175 | 3514, 3610 |
| 61, 241 | A5 | Auditory_5_Complex | Auditory_Association | 11 | 125 | 3346, 3881 |
| 62, 242 | STGa | Area_STGa | Auditory_Association | 11 | 123 | 2509, 2187 |
| 63, 243 | STSda | Area_STSd_anterior | Auditory_Association | 11 | 128 | 1944, 2389 |
| 64, 244 | STSdp | Area_STSd_posterior | Auditory_Association | 11 | 129 | 1994, 2605 |
| 65, 245 | STSva | Area_STSv_anterior | Auditory_Association | 11 | 176 | 1694, 1900 |
| 66, 246 | STSvp | Area_STSv_posterior | Auditory_Association | 11 | 130 | 2898, 2515 |
| 67, 247 | TA2 | Area_TA2 | Auditory_Association | 11 | 107 | 1518, 1726 |
| 68, 248 | AAIC | Anterior_Agranular_Insula_Complex | Insula_FrontalOperc | 12 | 112 | 1859, 1691 |
| 69, 249 | AVI | Anterior_Ventral_Insular_Area | Insula_FrontalOperc | 12 | 111 | 1446, 1792 |
| 70, 250 | FOP2 | Frontal_Opercular_Area_2 | Insula_FrontalOperc | 12 | 115 | 750, 720 |
| 71, 251 | FOP3 | Frontal_Opercular_Area_3 | Insula_FrontalOperc | 12 | 114 | 754, 614 |
| 72, 252 | FOP4 | Frontal_Opercular_Area_4 | Insula_FrontalOperc | 12 | 108 | 2522, 1678 |
| 73, 253 | FOP5 | Area_Frontal_Opercular_5 | Insula_FrontalOperc | 12 | 169 | 1297, 1365 |
| 74, 254 | Ig | Insular_Granular_Complex | Insula_FrontalOperc | 12 | 168 | 841, 1077 |
| 75, 255 | MI | Middle_Insular_Area | Insula_FrontalOperc | 12 | 109 | 2102, 1960 |
| 76, 256 | PI | Para-Insular_Area | Insula_FrontalOperc | 12 | 178 | 1033, 1058 |
| 77, 257 | Pir | Piriform_Cortex | Insula_FrontalOperc | 12 | 110 | 2287, 1856 |
| 78, 258 | PoI1 | Area_Posterior_Insular_1 | Insula_FrontalOperc | 12 | 167 | 1811, 1835 |
| 79, 259 | PoI2 | Posterior_Insular_Area_2 | Insula_FrontalOperc | 12 | 106 | 2747, 2675 |
| 80, 260 | H | Hippocampus | Medial_Temporal | 13 | 120 | 4283, 3626 |
| 81, 261 | PreS | PreSubiculum | Medial_Temporal | 13 | 119 | 1817, 1558 |
| 82, 262 | EC | Entorhinal_Cortex | Medial_Temporal | 13 | 118 | 2127, 2110 |
| 83, 263 | PeEc | Perirhinal_Ectorhinal_Cortex | Medial_Temporal | 13 | 122 | 4826, 4755 |
| 84, 264 | TF | Area_TF | Medial_Temporal | 13 | 135 | 3986, 4752 |
| 85, 265 | PHA1 | ParaHippocampal_Area_1 | Medial_Temporal | 13 | 126 | 1281, 1168 |
| 86, 266 | PHA2 | ParaHippocampal_Area_2 | Medial_Temporal | 13 | 155 | 783, 771 |
| 87, 267 | PHA3 | ParaHippocampal_Area_3 | Medial_Temporal | 13 | 127 | 2023, 1122 |
| 88, 268 | PHT | Area_PHT | Lateral_Temporal | 14 | 137 | 4182, 3410 |
| 89, 269 | TE1a | Area_TE1_anterior | Lateral_Temporal | 14 | 132 | 5227, 4180 |
| 90, 270 | TE1m | Area_TE1_Middle | Lateral_Temporal | 14 | 177 | 3339, 3429 |
| 91, 271 | TE1p | Area_TE1_posterior | Lateral_Temporal | 14 | 133 | 7116, 6010 |
| 92, 272 | TE2a | Area_TE2_anterior | Lateral_Temporal | 14 | 134 | 5691, 5753 |
| 93, 273 | TE2p | Area_TE2_posterior | Lateral_Temporal | 14 | 136 | 4115, 3040 |
| 94, 274 | TGd | Area_TG_dorsal | Lateral_Temporal | 14 | 131 | 10192, 10269 |
| 95, 275 | TGv | Area_TG_Ventral | Lateral_Temporal | 14 | 172 | 3694, 4515 |
| 96, 276 | PSL | PeriSylvian_Language_Area | TPO | 15 | 25 | 2154, 2759 |
| 97, 277 | STV | Superior_Temporal_Visual_Area | TPO | 15 | 28 | 2322, 2294 |
| 98, 278 | TPOJ1 | Area_TemporoParietoOccipital_Junction_1 | TPO | 15 | 139 | 2102, 3938 |
| 99, 279 | TPOJ2 | Area_TemporoParietoOccipital_Junction_2 | TPO | 15 | 140 | 1930, 2068 |
| 100, 280 | TPOJ3 | Area_TemporoParietoOccipital_Junction_3 | TPO | 15 | 141 | 1290, 1277 |
| 101, 281 | 7AL | Lateral_Area_7A | Superior_Parietal | 16 | 42 | 2134, 2030 |
| 102, 282 | 7Am | Medial_Area_7A | Superior_Parietal | 16 | 45 | 2995, 2379 |
| 103, 283 | 7PC | Area_7PC | Superior_Parietal | 16 | 47 | 3151, 3415 |
| 104, 284 | 7PL | Lateral_Area_7P | Superior_Parietal | 16 | 46 | 1695, 1363 |
| 105, 285 | 7Pm | Medial_Area_7P | Superior_Parietal | 16 | 29 | 1601, 1308 |
| 106, 286 | AIP | Anterior_IntraParietal_Area | Superior_Parietal | 16 | 117 | 1999, 2542 |
| 107, 287 | LIPd | Area_Lateral_IntraParietal_dorsal | Superior_Parietal | 16 | 95 | 1008, 869 |
| 108, 288 | LIPv | Area_Lateral_IntraParietal_ventral | Superior_Parietal | 16 | 48 | 1681, 1783 |
| 109, 289 | MIP | Medial_IntraParietal_Area | Superior_Parietal | 16 | 50 | 1872, 2403 |
| 110, 290 | VIP | Ventral_IntraParietal_Complex | Superior_Parietal | 16 | 49 | 1890, 1577 |
| 111, 291 | IP0 | Area_IntraParietal_0 | Inferior_Parietal | 17 | 146 | 1203, 1239 |
| 112, 292 | IP1 | Area_IntraParietal_1 | Inferior_Parietal | 17 | 145 | 1692, 1632 |
| 113, 293 | IP2 | Area_IntraParietal_2 | Inferior_Parietal | 17 | 144 | 2102, 1861 |
| 114, 294 | PF | Area_PF_Complex | Inferior_Parietal | 17 | 148 | 5457, 5251 |
| 115, 295 | PFm | Area_PFm_Complex | Inferior_Parietal | 17 | 149 | 8220, 8141 |
| 116, 296 | PFop | Area_PF_Opercular | Inferior_Parietal | 17 | 147 | 1797, 1783 |
| 117, 297 | PFt | Area_PFt | Inferior_Parietal | 17 | 116 | 1983, 2039 |
| 118, 298 | PGi | Area_PGi | Inferior_Parietal | 17 | 150 | 4791, 4970 |
| 119, 299 | PGp | Area_PGp | Inferior_Parietal | 17 | 143 | 2501, 3740 |
| 120, 300 | PGs | Area_PGs | Inferior_Parietal | 17 | 151 | 4552, 3366 |
| 121, 301 | 23d | Area_23d | Posterior_Cingulate | 18 | 32 | 1261, 1513 |
| 122, 302 | 31a | Area_31a | Posterior_Cingulate | 18 | 162 | 1260, 1116 |
| 123, 303 | 31pd | Area_31pd | Posterior_Cingulate | 18 | 161 | 1428, 864 |
| 124, 304 | 31pv | Area_31p_ventral | Posterior_Cingulate | 18 | 35 | 950, 1022 |
| 125, 305 | 7m | Area_7m | Posterior_Cingulate | 18 | 30 | 2128, 2067 |
| 126, 306 | d23ab | Area_dorsal_23_a+b | Posterior_Cingulate | 18 | 34 | 1607, 1106 |
| 127, 307 | DVT | Dorsal_Transitional_Visual_Area | Posterior_Cingulate | 18 | 142 | 1806, 2176 |
| 128, 308 | PCV | PreCuneus_Visual_Area | Posterior_Cingulate | 18 | 27 | 2245, 2416 |
| 129, 309 | POS1 | Parieto-Occipital_Sulcus_Area_1 | Posterior_Cingulate | 18 | 31 | 2531, 2727 |
| 130, 310 | POS2 | Parieto-Occipital_Sulcus_Area_2 | Posterior_Cingulate | 18 | 15 | 3261, 3093 |
| 131, 311 | ProS | ProStriate_Area | Posterior_Cingulate | 18 | 121 | 1222, 1055 |
| 132, 312 | RSC | RetroSplenial_Complex | Posterior_Cingulate | 18 | 14 | 2830, 3067 |
| 133, 313 | v23ab | Area_ventral_23_a+b | Posterior_Cingulate | 18 | 33 | 916, 1089 |
| 134, 314 | 10r | Area_10r | AntCing_MedPFC | 19 | 65 | 1589, 1053 |
| 135, 315 | 10v | Area_10v | AntCing_MedPFC | 19 | 88 | 3906, 2667 |
| 136, 316 | 25 | Area_25 | AntCing_MedPFC | 19 | 164 | 1911, 2135 |
| 137, 317 | 33pr | Area_33_prime | AntCing_MedPFC | 19 | 58 | 1354, 1316 |
| 138, 318 | 8BM | Area_8BM | AntCing_MedPFC | 19 | 63 | 3122, 3436 |
| 139, 319 | 9m | Area_9_Middle | AntCing_MedPFC | 19 | 69 | 6338, 5881 |
| 140, 320 | a24 | Area_a24 | AntCing_MedPFC | 19 | 61 | 2085, 2152 |
| 141, 321 | a24pr | Anterior_24_prime | AntCing_MedPFC | 19 | 59 | 1095, 1474 |
| 142, 322 | a32pr | Area_anterior_32_prime | AntCing_MedPFC | 19 | 179 | 1759, 1118 |
| 143, 323 | d32 | Area_dorsal_32 | AntCing_MedPFC | 19 | 62 | 2228, 2374 |
| 144, 324 | p24 | Area_posterior_24 | AntCing_MedPFC | 19 | 180 | 2394, 2442 |
| 145, 325 | p24pr | Area_Posterior_24_prime | AntCing_MedPFC | 19 | 57 | 1422, 1724 |
| 146, 326 | p32 | Area_p32 | AntCing_MedPFC | 19 | 64 | 1180, 1765 |
| 147, 327 | p32pr | Area_p32_prime | AntCing_MedPFC | 19 | 60 | 1569, 1305 |
| 148, 328 | pOFC | Posterior_OFC_Complex | AntCing_MedPFC | 19 | 166 | 2486, 2836 |
| 149, 329 | s32 | Area_s32 | AntCing_MedPFC | 19 | 165 | 604, 1015 |
| 150, 330 | 10d | Area_10d | OrbPolaFrontal | 20 | 72 | 3644, 3096 |
| 151, 331 | 10pp | Polar_10p | OrbPolaFrontal | 20 | 90 | 1997, 2487 |
| 152, 332 | 11l | Area_11l | OrbPolaFrontal | 20 | 91 | 3531, 3793 |
| 153, 333 | 13l | Area_13l | OrbPolaFrontal | 20 | 92 | 2429, 1757 |
| 154, 334 | 47m | Area_47m | OrbPolaFrontal | 20 | 66 | 799, 781 |
| 155, 335 | 47s | Area_47s | OrbPolaFrontal | 20 | 94 | 2795, 3080 |
| 156, 336 | a10p | Area_anterior_10p | OrbPolaFrontal | 20 | 89 | 1964, 1748 |
| 157, 337 | OFC | Orbital_Frontal_Complex | OrbPolaFrontal | 20 | 93 | 4560, 5232 |
| 158, 338 | p10p | Area_posterior_10p | OrbPolaFrontal | 20 | 170 | 2116, 2365 |
| 159, 339 | 44 | Area_44 | Inferior_Frontal | 21 | 74 | 2435, 2589 |
| 160, 340 | 45 | Area_45 | Inferior_Frontal | 21 | 75 | 3762, 2962 |
| 161, 341 | 47l | Area_47l_(47_lateral) | Inferior_Frontal | 21 | 76 | 2527, 2592 |
| 162, 342 | a47r | Area_anterior_47r | Inferior_Frontal | 21 | 77 | 4167, 3763 |
| 163, 343 | IFJa | Area_IFJa | Inferior_Frontal | 21 | 79 | 1513, 1405 |
| 164, 344 | IFJp | Area_IFJp | Inferior_Frontal | 21 | 80 | 960, 740 |
| 165, 345 | IFSa | Area_IFSa | Inferior_Frontal | 21 | 82 | 2057, 2641 |
| 166, 346 | IFSp | Area_IFSp | Inferior_Frontal | 21 | 81 | 1589, 1730 |
| 167, 347 | p47r | Area_posterior_47r | Inferior_Frontal | 21 | 171 | 2133, 1761 |
| 168, 348 | 46 | Area_46 | Dorsolateral_Prefrontal | 22 | 84 | 4863, 4394 |
| 169, 349 | 8Ad | Area_8Ad | Dorsolateral_Prefrontal | 22 | 68 | 3386, 3492 |
| 170, 350 | 8Av | Area_8Av | Dorsolateral_Prefrontal | 22 | 67 | 4807, 5902 |
| 171, 351 | 8BL | Area_8B_Lateral | Dorsolateral_Prefrontal | 22 | 70 | 3377, 4078 |
| 172, 352 | 8C | Area_8C | Dorsolateral_Prefrontal | 22 | 73 | 4085, 3134 |
| 173, 353 | 9-46d | Area_9-46d | Dorsolateral_Prefrontal | 22 | 86 | 4534, 4666 |
| 174, 354 | 9a | Area_9_anterior | Dorsolateral_Prefrontal | 22 | 87 | 3706, 3048 |
| 175, 355 | 9p | Area_9_Posterior | Dorsolateral_Prefrontal | 22 | 71 | 3426, 2488 |
| 176, 356 | a9-46v | Area_anterior_9-46v | Dorsolateral_Prefrontal | 22 | 85 | 3314, 2628 |
| 177, 357 | i6-8 | Inferior_6-8_Transitional_Area | Dorsolateral_Prefrontal | 22 | 97 | 1764, 2418 |
| 178, 358 | p9-46v | Area_posterior_9-46v | Dorsolateral_Prefrontal | 22 | 83 | 2871, 4635 |
| 179, 359 | s6-8 | Superior_6-8_Transitional_Area | Dorsolateral_Prefrontal | 22 | 98 | 1336, 2132 |
| 180, 360 | SFL | Superior_Frontal_Language_Area | Dorsolateral_Prefrontal | 22 | 26 | 3873, 3055 |

Column 1 (Reordered ID) shows the order in HCPex based on the HCP-MMP1_UniqueRegionList.csv, as described in the Methods, of the 360 cortical regions originally defined by Glasser et al (2016). The names of the cortical divisions shown in column 4 come from the same .csv file. The sixth column shows the original order used by Glasser et al (2016). Abbreviations: L=left hemisphere, R=right. MT+_Complex, MT+_Complex_and_Neighboring_Visual_Areas; SomaSens_Motor, Somatosensory_and_Motor; ParaCentral_MidCing, Paracentral_Lobular_and_Mid_Cingulate; Insula_FrontalOperc, Insular_and_Frontal_Opercular; TPO, Temporo-Parieto-Occipital_Junction; AntCing_MedPFC, Anterior_Cingulate_and_Medial_Prefrontal; OrbPolaFrontal, Orbital_and_Polar_Frontal.

**Table S2. The subcortical regions in the extended HCPex atlas (Huang, Rolls et al (2022)).**

| **ID (L,R)** | **Abbreviation** | **Full Name** | **Voxel numbers (1mm3) (L,R)** |
| --- | --- | --- | --- |
| 361, 394 | AV | Thalamus: Anteroventral Nucleus | 256, 280 |
| 362, 395 | CeM | Thalamus: Central medial | 96, 88 |
| 363, 396 | CL | Thalamus: Central lateral | 16, 16 |
| 364, 397 | CM | Thalamus: Centralmedian | 424, 376 |
| 365, 398 | LD | Thalamus: Laterodorsal | 48, 8 |
| 366, 399 | LGN | Thalamus: Lateral Geniculate | 328, 256 |
| 367, 400 | LP | Thalamus: Lateral Posterior | 256, 264 |
| 368, 401 | L-Sg | Thalamus: Limitans Suprageniculate | 32, 16 |
| 369, 402 | MDl | Thalamus: Mediodorsolateral parvocellular | 328, 320 |
| 370, 403 | MDm | Thalamus: Mediodorsomedial magnocellular | 1104, 1192 |
| 371, 404 | MGN | Thalamus: Medial Geniculate | 160, 168 |
| 372, 405 | MV(Re) | Thalamus: Reuniens | 8, 8 |
| 373, 406 | Pf | Thalamus: Parafascicular | 56, 72 |
| 374, 407 | PuA | Thalamus: Pulvinar anterior | 320, 280 |
| 375, 408 | PuI | Thalamus: Pulvinar inferior | 336, 280 |
| 376, 409 | PuL | Thalamus: Pulvinar lateral | 304, 256 |
| 377, 410 | PuM | Thalamus: Pulvinar medial | 1904, 1680 |
| 378, 411 | VA | Thalamus: Ventral Anterior | 608, 616 |
| 379, 412 | VLa | Thalamus: Ventral Lateral Anterior | 880, 856 |
| 380, 413 | VLp | Thalamus: Ventral Lateral Posterior | 1384, 1288 |
| 381, 414 | VPL | Thalamus: Ventral posterolateral | 1600, 1368 |
| 382, 415 | Putam | Putamen | 7896, 7744 |
| 383, 416 | Caud | Caudate | 6896, 6952 |
| 384, 417 | NAc | Nucleus Accumbens | 600, 632 |
| 385, 418 | Gpe | Globus pallidus externalis | 1232, 1144 |
| 386, 419 | Gpi | Globus pallidus internalis | 632, 624 |
| 387, 420 | Amyg | Amygdala | 1608, 1632 |
| 388, 421 | SNpc | Substantia nigra pars compacta | 184, 200 |
| 389, 422 | SNpr | Substantia nigra pars reticulata | 408, 440 |
| 390, 423 | VTA | Ventral tegmental area | 40, 48 |
| 391, 424 | MB | Mammillary bodies | 112, 104 |
| 392, 425 | Septum | Septal nuclei | 248, 136 |
| 393, 426 | Nb | Nucleus basalis | 584, 600 |

Fig. S1-1. Example coronal slices showing regions defined in the HCPex atlas and added subcortical regions. The abbreviations are as in Tables S1 and S2. The y values for the coronal slices are in MNI coordinates.


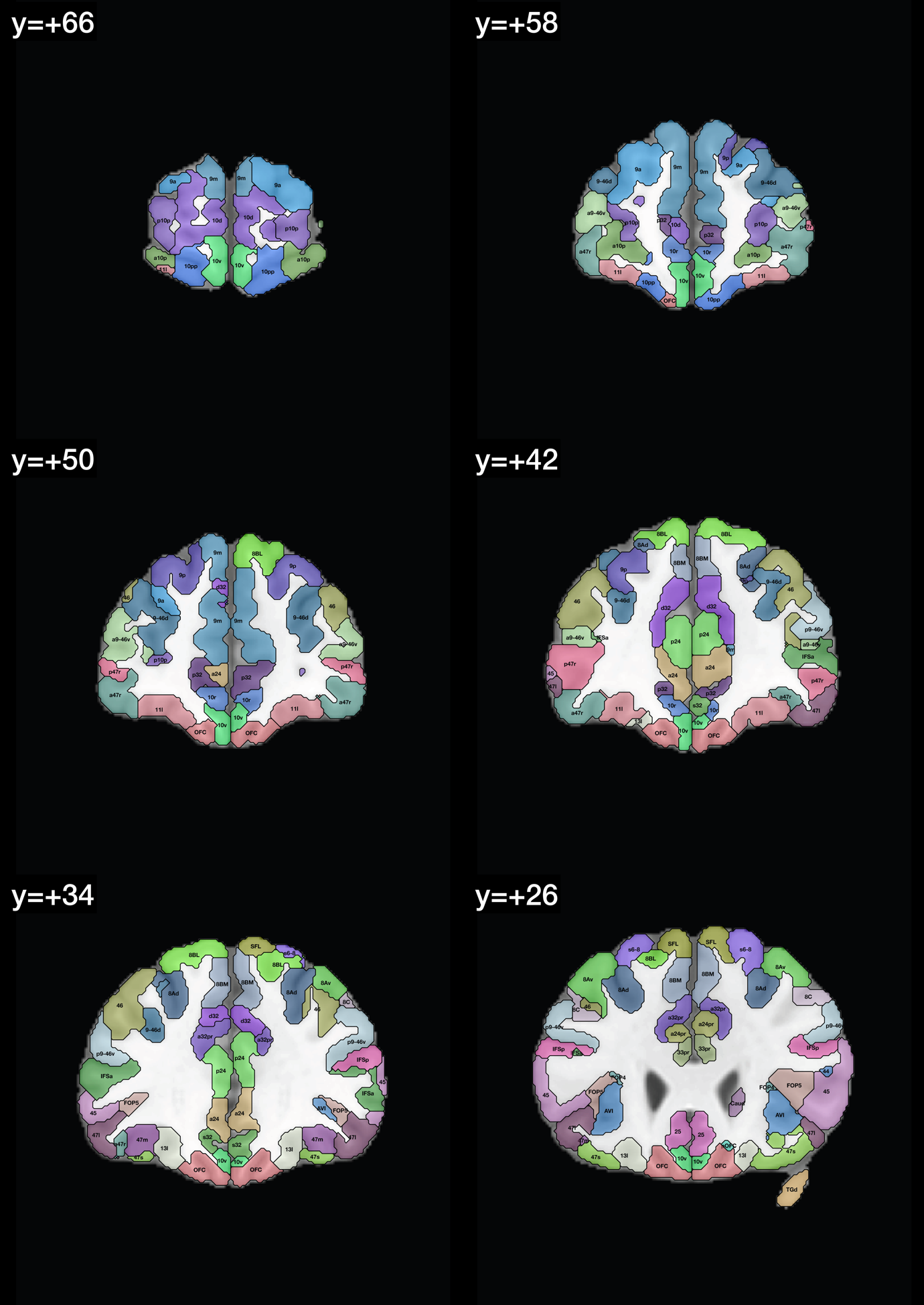


Fig. S1-2. Example coronal slices showing regions defined in the HCPex atlas and added subcortical regions. The abbreviations are as in Tables S1 and S2. The y values for the coronal slices are in MNI coordinates.


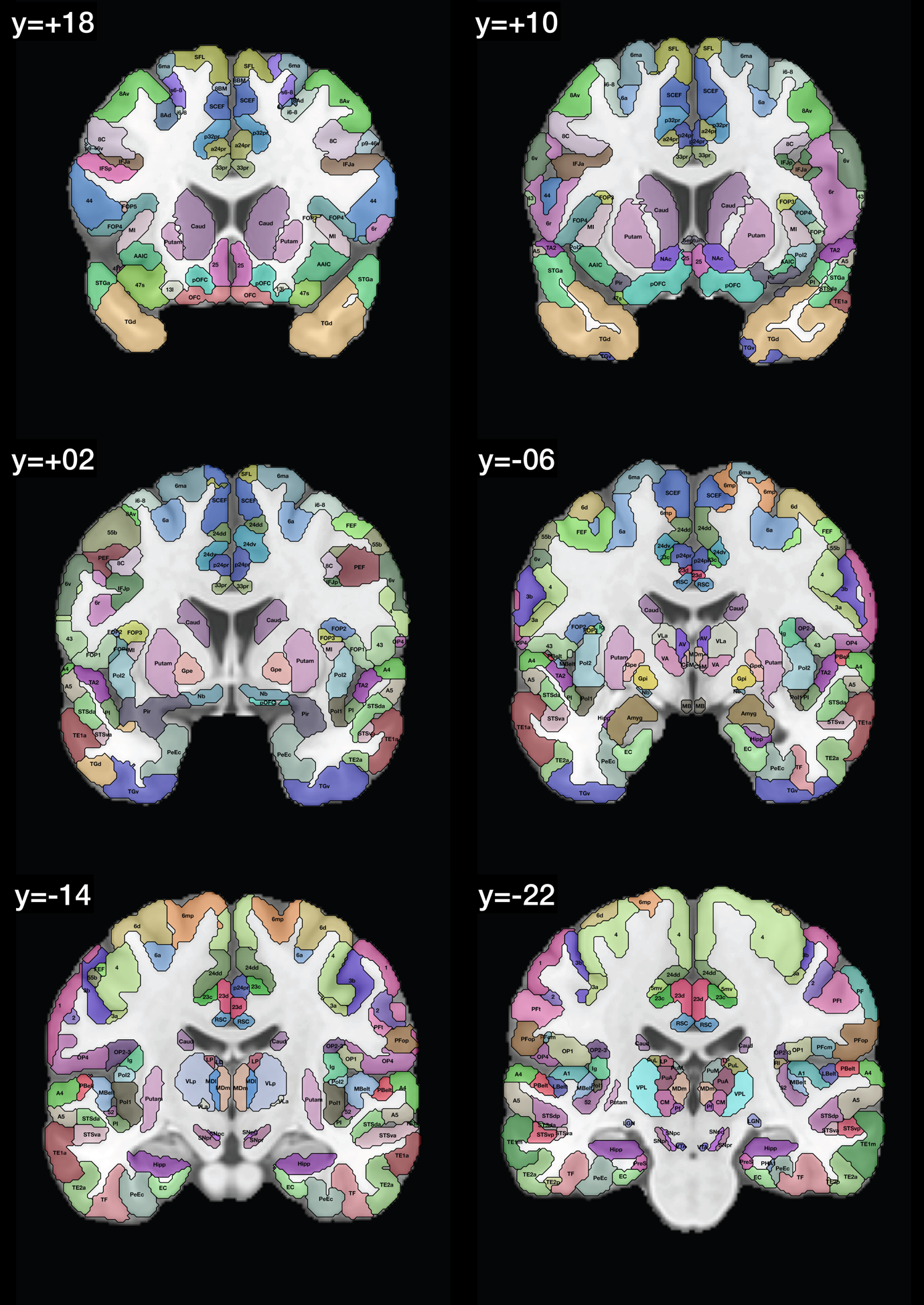


Fig. S1-3. Example coronal slices showing regions defined in the HCPex atlas and added subcortical regions. The abbreviations are as in Tables S1 and S2. The y values for the coronal slices are in MNI coordinates.


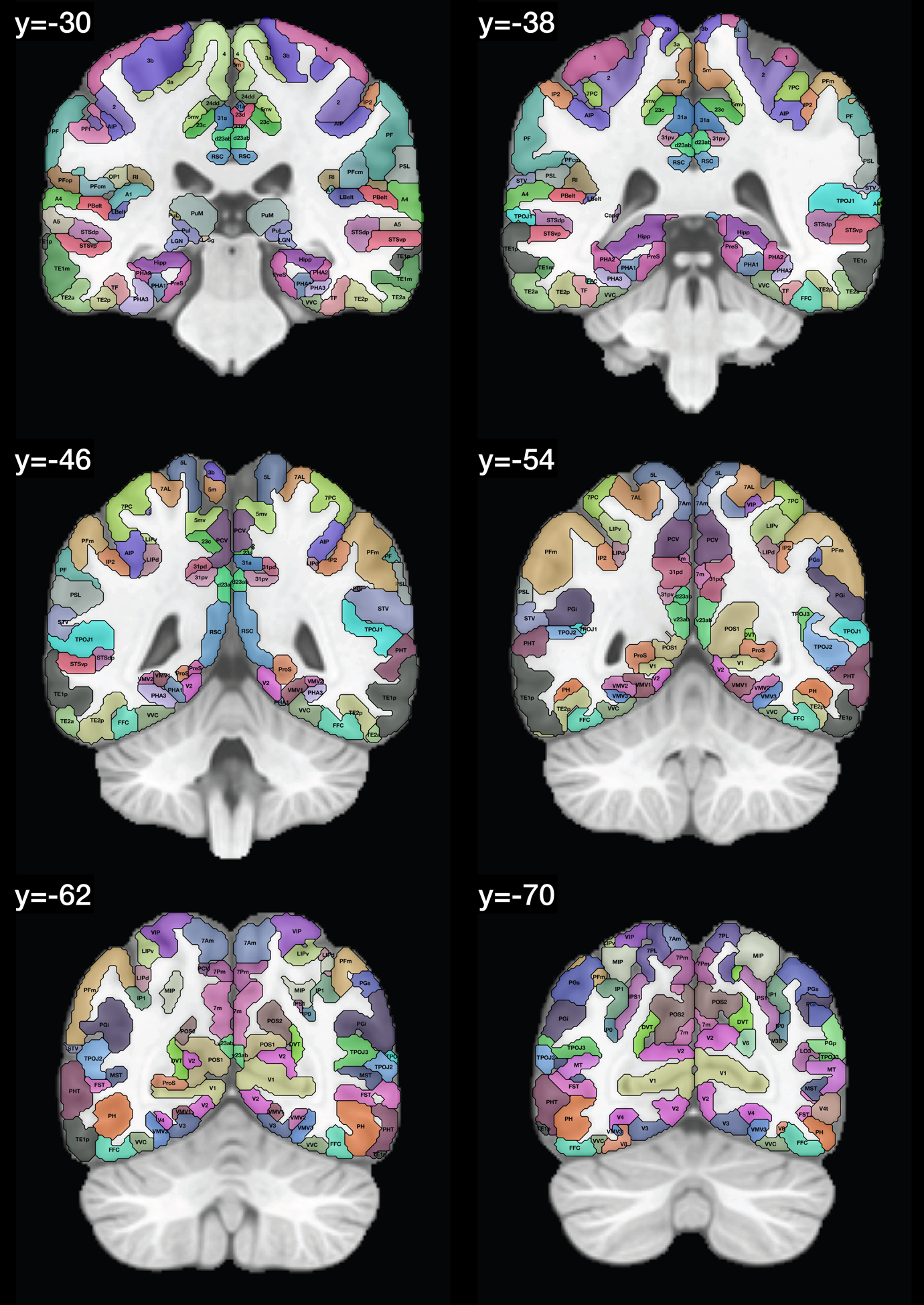


Fig. S1-4. Example coronal slices showing regions defined in the HCPex atlas and added subcortical regions. The abbreviations are as in Tables S1 and S2. The y values for the coronal slices are in MNI coordinates.


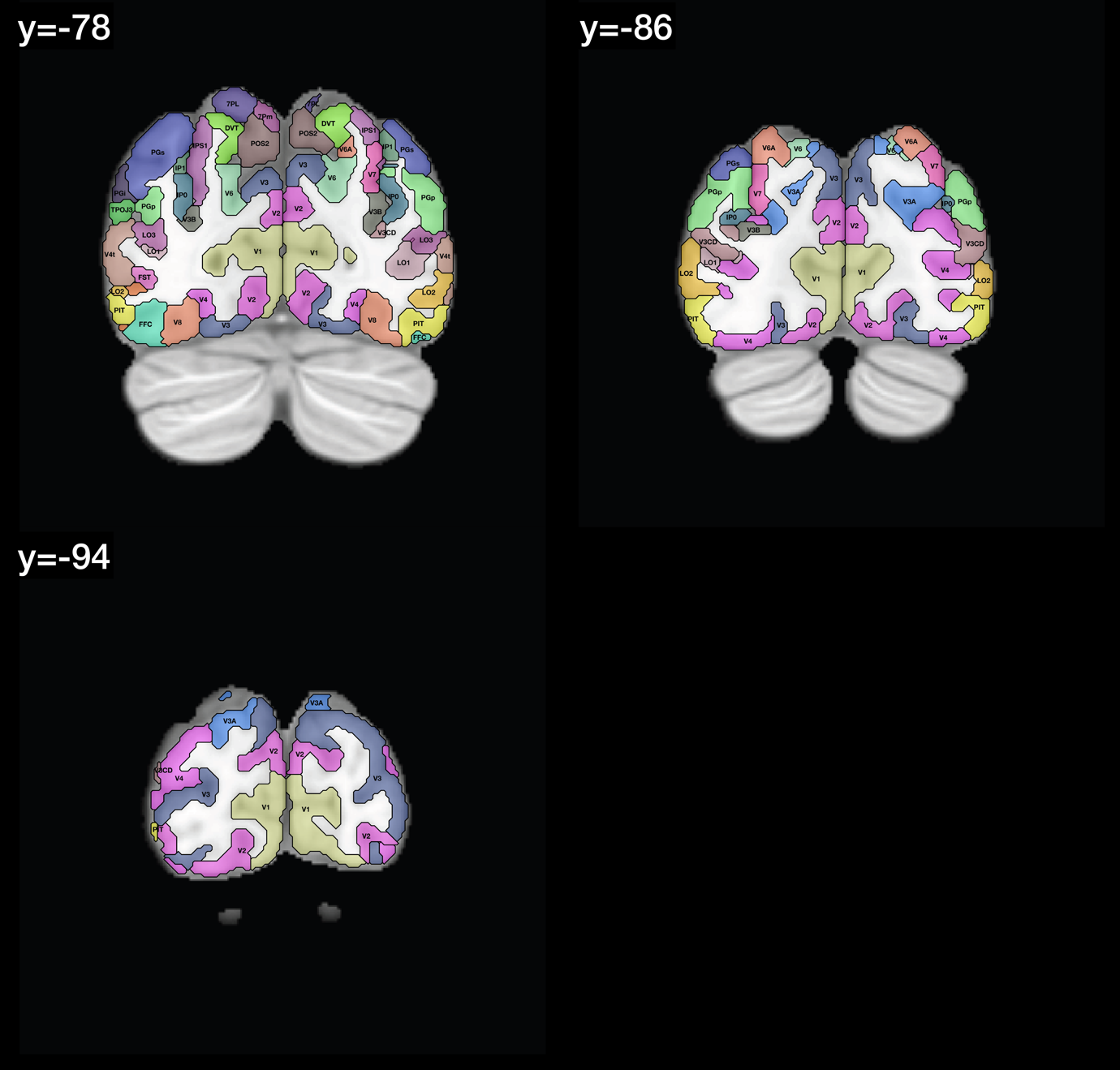


Fig. S1-5. Cortical areas from the HCP-MMP1 atlas (Glasser, et al., 2016) shown on views of the human brain with the sulci opened sufficiently to show the cortical areas in the sulci, and the subcortical areas that are also included in the extended atlas HCPex (Huang, et al., 2022). The abbreviations for the different brain regions are provided in Tables S1 and S2.


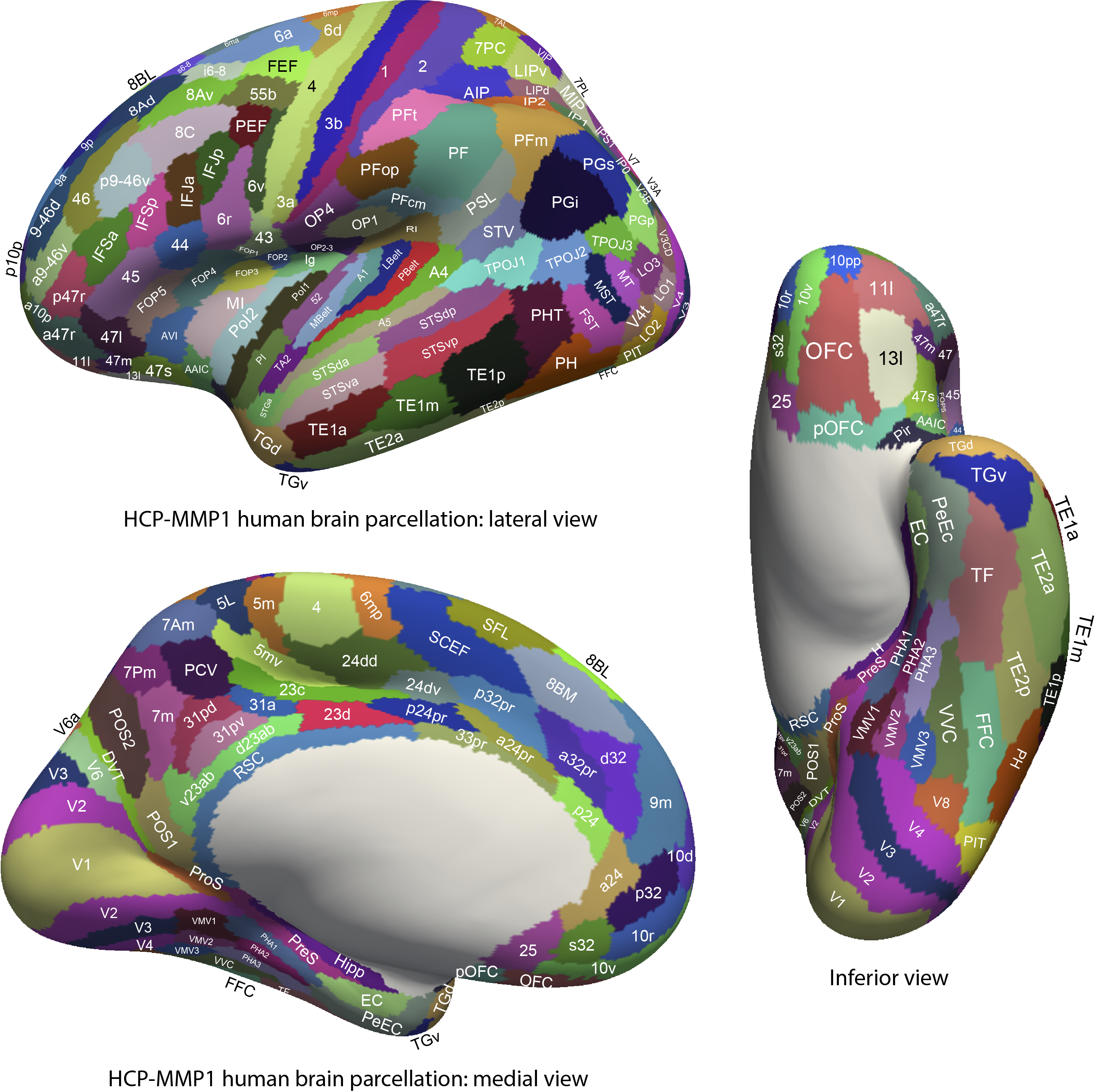


Fig. S1-6. Anatomical regions of the human visual and other cortical regions. Regions are shown as defined in the HCP-MMP atlas (Glasser, et al., 2016), and in its extended version HCPex (Huang, et al., 2022). The regions are shown on images of the human brain without the sulci expanded to show which cortical HCP-MMP regions are normally visible, for comparison with Fig. S1-5. (The ICBM153 MNI T1 image was used to prepare this figure.) Abbreviations are provided in Table S1.


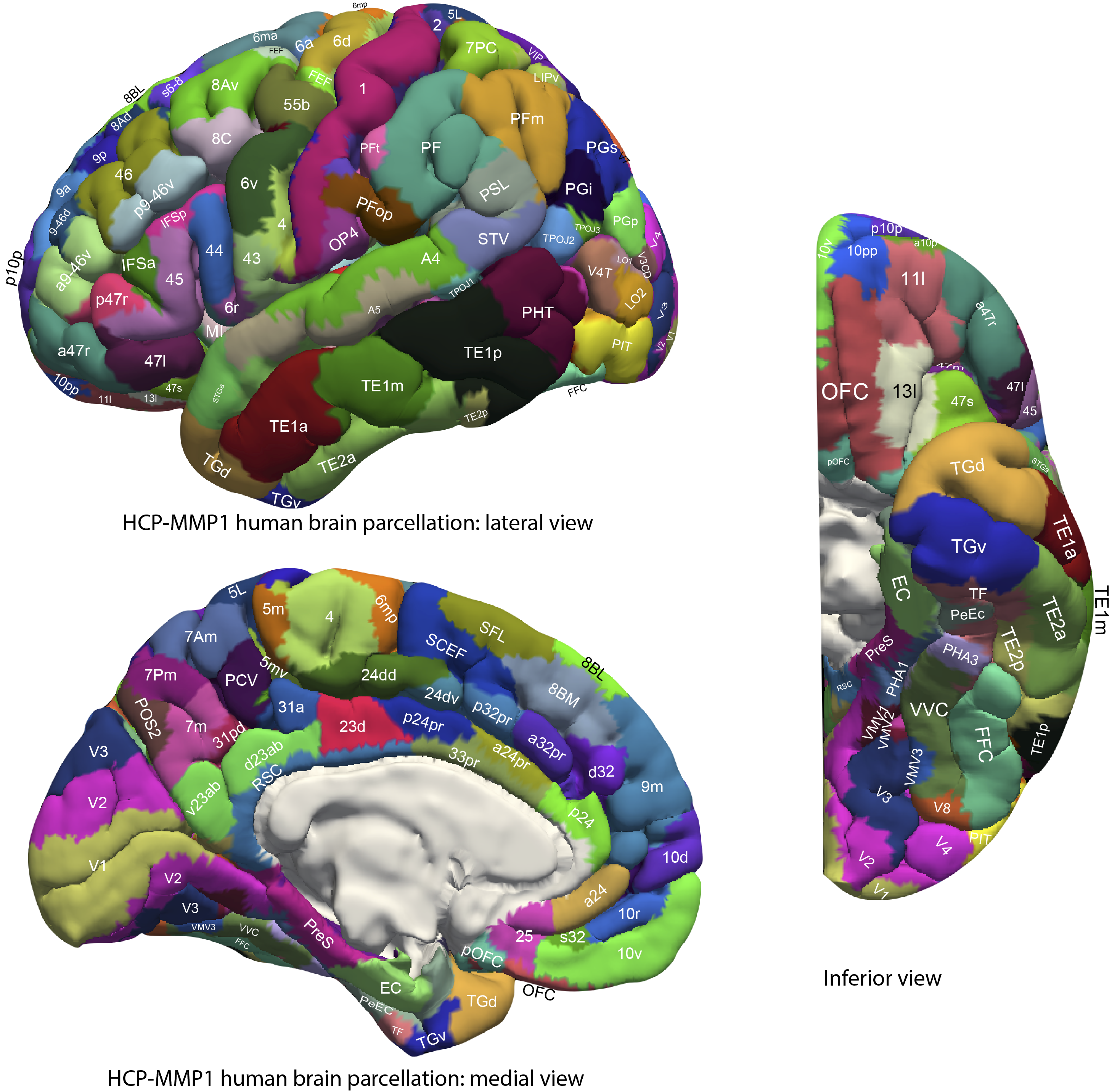


**Details of the Hopf Effective Connectivity algorithm**

*Introduction*

Effective connectivity measures the effect of one brain region on another, and utilizes differences detected at different times in the signals in each connected pair of brain regions to infer effects of one brain region on another. One such approach is dynamic causal modelling, but is applied most easily to activation studies, and is typically limited to measuring the effective connectivity between just a few brain areas (Friston, 2009; Valdes-Sosa, et al., 2011; Bajaj, et al., 2016), though there have been moves to extend it to resting state studies and more brain areas (Frassle, et al., 2017; Razi, et al., 2017). The method used here (Rolls, et al., 2022) was developed from a Hopf algorithm to enable measurement of effective connectivity between many brain areas, described by Deco et al (2019). A principle is that the functional connectivity is measured at time *t* and time *t* + *tau*, where *tau* is typically 2 s to take into account the time within which a change in the BOLD signal can occur, and then the effective connectivity model is trained by error correction until it can generate the functional connectivity matrices at time *t* and time *t* + *tau*. Further details of the algorithm, and the development that enabled it to measure the effective connectivity in each direction (Rolls, et al., 2022), are described next.

To measure the effective connectivity, we use a whole-brain model that allows us to simulate the BOLD activity across all brain regions and time. We use the so-called Hopf computational model, which integrates the dynamics of Stuart-Landau oscillators, to enable the activity (in this case the BOLD signal) of each brain region to be generated from the underlying effective connectivity in both directions between every pair of brain regions (Deco, et al., 2017b). As mentioned above, we include in the model 360 cortical brain areas (Huang, et al., 2022). The local dynamics of each brain area (node) which simulate the BOLD signal are given by Stuart-Landau oscillators which express the normal form of a supercritical Hopf bifurcation, describing the transition from noisy to oscillatory dynamics (Kuznetsov, 2013). It has been shown that the Hopf whole-brain model successfully simulates empirical electrophysiology (Freyer, et al., 2011; Freyer, et al., 2012), MEG (Deco, et al., 2017a) and fMRI (Kringelbach, et al., 2015; Deco, et al., 2017b; Kringelbach and Deco, 2020). The development of the Hopf algorithm that enables it to measure the effective connectivity in both directions between every pair of nodes is described by Rolls, Deco et al (2022).

*Overview of the effective connectivity measurement algorithm*

The steps of the algorithm can be summarized as follows:

1. From the empirically measured time series of the BOLD signal for each of *N* brain areas bandpass filtered between 0.008 and 0.08 Hz we calculate the *N*x*N* empirical functional connectivity matrix FC^emp^ by the Pearson correlation between the time series of each pair of brain regions. We also create an *FC*^tau_emp^ *N*x*N* lagged time series matrix in which the entry for each brain region is the correlation between the BOLD signal at time *t* and *t*+*tau* calculated over the whole empirical time series. *Tau* is typically set to 2 s, a minimal useful period in the BOLD signal in which a change can be detected. The lagged correlation matrix FC^tau_emp^ provides the delayed information that enables the effective connectivity to be measured in both directions between each pair of nodes, the brain regions.

2. The *N*x*N* effective connectivity (EC) matrix to be calculated can be initialized with zeros, or with a structural connection matrix obtained from for example diffusion MRI. The effective connectivity matrix is read by convention from column to row, with the effective connectivity between each pair of nodes (brain regions) 1:*N* in one direction shown in the lower left triangle, and the effective connectivity in the opposite direction in the upper right triangle. If the EC matrix is initialized with a structural connection matrix, this can have the potential advantage that nodes with no possible anatomical connection can be left at 0 and ignored in the calculations, which has the potential to increase the accuracy of the algorithm for a given number of nodes in the EC matrix, as fewer nodes need to be taken into consideration in calculating the updates to the EC matrix. If the EC matrix is initialized with zeros, this has the potential advantage that any errors in the structural connectivity matrix cannot influence the results. In practice, it has been found that with up to 360 brain areas and typical time series for the BOLD signal and structural connectivity matrices, the effective connectivity can be calculated as well with the initialization with zeros as with the structural connectivity initialization (with correlations between the ECs calculated in these two ways typically 0.98), and therefore the initialization with zeros is used in the work described, as it makes fewer assumptions.

3. The ‘natural oscillation frequency’ (or ‘intrinsic frequency’) of each brain region or node is measured as the frequency with the peak power from the power spectrum of the BOLD signal for each node.

4. A Stuart-Landau oscillatory system with the *x* oscillatory component for each of the *N* nodes (its ‘natural oscillation frequency’ measured from the BOLD signal) and the *y* oscillatory components provided with the same ‘natural oscillation frequency’ parameters is simulated with a Hopf model. The *N* oscillators are connected by the EC matrix, and noise is injected into the system so that it just oscillates. This oscillatory system is simulated to generate simulated BOLD signals for each of the *N* brain areas.

5. The EC matrix is then updated over a series of iterations using gradient descent. The error signal is the difference between *FC,* the simulated functional connectivity matrix from the current EC matrix, and FC^emp^, the empirically measured functional connectivity matrix, together with the corresponding difference between the simulated FC^tau^ and the empirical FC^tau_emp^ matrix.

6. The EC matrix is that which has been computed when the correlations between the simulated and empirical FC matrices, and the simulated matrix FC^tau^ and the empirical FC^tau_emp^ are at their maximum, which are typically 0.75-0.8 after 50 iterations.

*The Hopf whole brain model using Stuart-Landau oscillators*

The Hopf whole-brain model, which integrates the activity of Stuart-Landau oscillators expressing the activity of each brain region *i* can be expressed mathematically as follows:

$\frac{dx_{i}}{dt}= \overset{Local Dynamics}{\overbrace{\left[ a_{i}-x_{i}^{2}-y_{i}^{2} \right]x_{i}-\omega_{i}y_{i}}} +\overset{Coupling}{\overbrace{G\sum_{j=1}^{N} C_{ij}\left( x_{j}-x_{i} \right)}} +\overset{Gaussian Noise}{\overbrace{\beta\eta_{i}\left( t \right)}}$ (1)

$\frac{dy_{i}}{dt}= \left[ a_{i}-x_{i}^{2}-y_{i}^{2} \right]y_{i}+\omega_{i}x_{i} + G\sum_{j=1}^{N} C_{ij}\left( y_{j}-y_{i} \right) + \beta\eta_{i}\left( t \right)$ (2)

The pair ($x_{i}(t),y_{i}(t))$ represent the state of the dynamical system modelling brain area (node) *i*, given its interactions with all other brain areas, at a given time *t*. Equations 1 and 2 describe the dynamics of this system in Cartesian coordinates, where the $x_{i}(t)$ term represents the simulated BOLD signal data of brain area *i*. The values of $y_{i}(t)$ are relevant to the dynamics of the system but are not part of the information read out from the system.

Equations 1 and 2 describe the coupling of Stuart-Landau oscillators through an effective connectivity matrix *C*. In these equations, $\eta_{i}\left( t \right)$ provides additive Gaussian noise with standard deviation β. The Stuart-Landau oscillators for each brain area *i* express a Hopf normal form that has a supercritical bifurcation at $a_{i}$=0, so that if $a_{i}$>0 the system has a stable limit cycle with frequency $f_{i}$*=*$\omega_{i}$*/2π* (where $\omega_{i}$ is the angular velocity), and when $a_{i}$<0 the system has a stable fixed point representing a low activity noisy state. The intrinsic frequency $f_{i}$ of each Stuart-Landau oscillator corresponding to a brain area is in the 0.008–0.08 Hz band (*i*=1, …, 360). The intrinsic frequencies are fitted from the data, as given by the frequency with the peak power of the narrowband BOLD signals of each brain region. The coupling term in Equations 1 and 2 representing the input received in node *i* from every other node *j*, is weighted by the corresponding effective connectivity $C_{ij}$. The coupling is the canonical diffusive coupling, which approximates the simplest (linear) part of a general coupling function (Deco, et al., 2019). *G* denotes the global coupling weight, scaling equally the total input received in each brain area. With the oscillators weakly coupled, the periodic orbit of the uncoupled oscillators is preserved.

Further insight can be obtained as follows. The local dynamics of each brain area are that of a Stuart-Landau oscillator, and in Equations 1 and 2 they are shown in Cartesian coordinates. However for insight into their dynamics they can be re-expressed in polar coordinates. This is performed by taking $r_{i}(t)$ to be $\sqrt{{x_{i}(t)}^{2}+{y_{i}(t)}^{2}}$, which can be interpreted as the amplitude of the Stuart-Landau oscillator modelling node *i* at time t, and $\theta_{i}(t)$ to be $arctan\left( \frac{y_{i}(t)}{x_{i}(t)} \right)$, which can be interpreted as the angle by which the oscillator for node *i* has rotated by time *t*. The coordinate transform yields the following equations for the local dynamics: $\dot{r}_{i}=\left( a_{i}-{r_{i}}^{2} \right)r_{i}, \dot{\theta}_{i}=\omega_{i}$. Hence the local dynamics of each brain region have a rate of change of $\theta$ with respect to time (a rate of oscillation) that is constant. Similarly, we see that the rate of change of amplitude with respect to time will vanish if and only if $r_{i}$=0 or $r_{i}=\sqrt{a_{i}}$, (clearly only possible if $a_{i}\geq0)$. On closer inspection of the equation governing $r_{i}$ we see for $a_{n}\leq$0 that$\dot{r}_{i}$ is strictly negative for all non-zero values of $r_{i}$, hence the system converges towards a state of no amplitude. For $a_{n}>$0 we see for all non-zero values of $r_{i}$ that $\dot{r}_{i}$ is strictly negative for $r_{i}>\sqrt{a_{i}}$ and strictly positive for $r_{i}<\sqrt{a_{i}}$, so all systems that are initialised with a non-zero amplitude converge to a state where $r_{i}=\sqrt{a_{i}}$.

To put this more formally, the system undergoes a supercritical bifurcation at $a_{i}$=0, so that if $a_{i}$>0 the system has a stable limit cycle given by $r_{i}=\sqrt{a_{i}},$ $\dot{\theta}_{i}=\omega_{i}$ (with frequency $f_{i}=\omega_{i}/2\pi$), and if $a_{i}$<0 the system has a stable fixed point $r_{i}=0$. However, such asymptotic stability of the model is rather unrealistic. The value of $\beta$ in the Hopf whole-brain model is the standard deviation of the Gaussian noise, and this is chosen to be sufficiently high that for a value of $a_{i}$ close to the bifurcation point, such asymptotic stability is avoided. The intrinsic frequency $f_{i}$ for each brain region is determined as follows. For each brain area *i*, the empirical time-series data is averaged across participants and through a discrete Fourier transform the modal frequency (that with the peak power), with the exclusion of high frequency noise, is obtained and set to be the intrinsic frequency of the given brain area. The intrinsic frequency $f_{i}$ of each Stuart-Landau oscillator corresponding to a brain area is in the 0.008–0.08 Hz band (*i*=1, …, 360).

The coupling term in Equations 1 and 2 acts to align the phases and frequencies of the oscillators in connected brain regions, and represents the input received in node *i* from every other node *j* and is weighted by the corresponding effective connectivity $C_{ij}$. The $(x_{j}-x_{i})$ term acts to force the dynamics of brain region *i* to more closely match and indeed synchronise with brain region *j* (with $C_{ij}$ > 0).

*Gradient descent to optimize the effective connectivity matrix*

The effective connectivity matrix is found by gradient descent from its initial value, informed by errors in functional connectivity predictions made using the Hopf model from the current effective connectivity matrix. The gradient descent is performed in order to fit the simulated to the empirical functional connectivity (FC) pairs and the lagged FC(tau) pairs. By this, we are able to infer a non-symmetric Effective Connectivity matrix (see Gilson et al (2016)). Note that FC^tau^*,* ie the lagged functional connectivity between pairs, lagged at tau s, breaks the symmetry and thus is fundamental for our purpose. Specifically, we compute the distance between the simulated model FC and empirical data FC^emp^, as well as the simulated model FC^tau^ and data FC^tau_emp^ and adjust each effective connection (entry in the effective connectivity matrix) separately with a gradient-descent approach. The model is run repeatedly with the updated effective connectivity until the fit converges towards a stable value. The update rule for an entry $C_{ij}$in the effective connectivity matrix is

$C_{ij}=C_{ij}+\varepsilon\left( FC_{ij}^{emp}-FC_{ij}+FC_{ij}^{tau\_emp}-FC_{ij}^{tau} \right)$ (3)

where $\epsilon is$a learning rate constant, and *i* and *j* are the nodes.

For the implementation, we set *tau* to be 2 s, selecting the appropriate number of TRs to achieve this.

The convergence of the algorithm is illustrated elsewhere (Rolls, et al., 2022). The correlations between the empirical functional connectivities and those simulated from the estimated effective connectivities for both time *t* and *t+tau* reach values close to 0.8.

*Interpretation of the effective connectivity measured by the Hopf algorithm*

If the Hopf effective connectivity algorithm is used with an anatomical connectivity mask, then the effective connectivity for anatomically unconnected nodes (brain areas) is not updated by the effective connectivity algorithm. This enables the algorithm to measure what might be termed ‘anatomical effective connectivity’. We note that in practice the anatomical maps measured with diffusion tractography are not very sparse (Huang, et al., 2021), so that only some links are not included in the effective connectivity map that is produced. We also note that if there are any errors in the diffusion tractography connection matrix, for example some missing anatomical connection links, then those links will not be included in the effective connectivity map.

If the Hopf effective connectivity algorithm is initialized with zeros, then all connectivities in the matrix can be updated by the algorithm. This ensures that there are no errors in the effective connectivity map that is generated by the algorithm due to any imperfections in the anatomical connection matrix. The effective connectivity calculated in this way reflects signals in one part of the brain that follow signals in another part of the brain with a time delay that is termed here τ (*tau*), independently of whether there is a direct anatomical connection or not. This is analogous to dynamic causal modelling and most applications of Granger causality to brain connectivity (Friston, et al., 2014; Bajaj, et al., 2016; Frassle, et al., 2017; Razi, et al., 2017), which impose no anatomical constraints on possible pathways between the nodes, i.e. the brain regions.

In practice, we have found that with the anatomical connection map we generated using diffusion tractography (Huang, et al., 2021), which is not very sparse, the effective connectivity matrices generated when starting with the anatomical connection matrix and the initial matrix with zeros are very similar, with typical correlations of 0.98. This is reassuring, and indicates that possible imperfections in the anatomical connection map do not produce problems in the effective connectivity matrix; and correspondingly that the Hopf effective connectivity algorithm assigns zero or close to zero effective connectivities when there is no known anatomical connection between a pair of brain regions. If a different very sparse anatomical connection matrix was used to initialize the Hopf effective connectivity algorithm, then the correlation might be lower. In practice, we prefer the initialization with the zeros in the connection matrix, as is makes fewer assumptions, but always check the results when the algorithm is initialized with an anatomical map. The analyses described in this paper used initialization of the effective connectivity matrices with zeros.


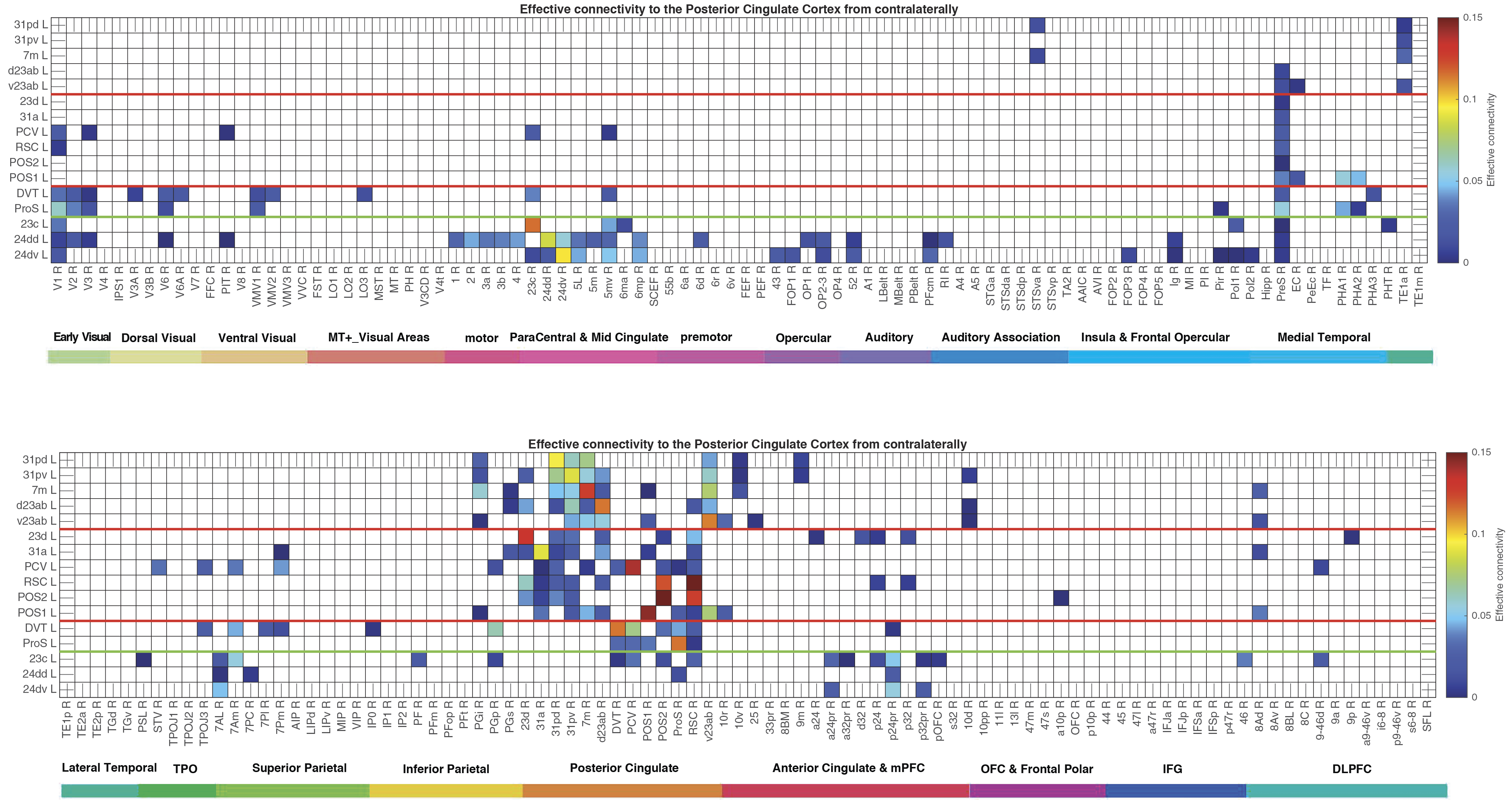


Fig. S2. Effective connectivity to the Left (L) posterior cingulate division regions and mid-cingulate cortex from all 180 cortical areas in the Right (R) hemisphere. All effective connectivities greater than 0 are shown, and effective connectivities of 0 are shown as a blank. The connectivities from the first set of cortical regions are shown above, and from the second set below. The three groups of posterior cingulate cortex / RSC areas are separated by red lines; and the mid-cingulate cortex effective connectivity is shown below the green line. Abbreviations: see Table S1. The effective connectivity is read from column to row.


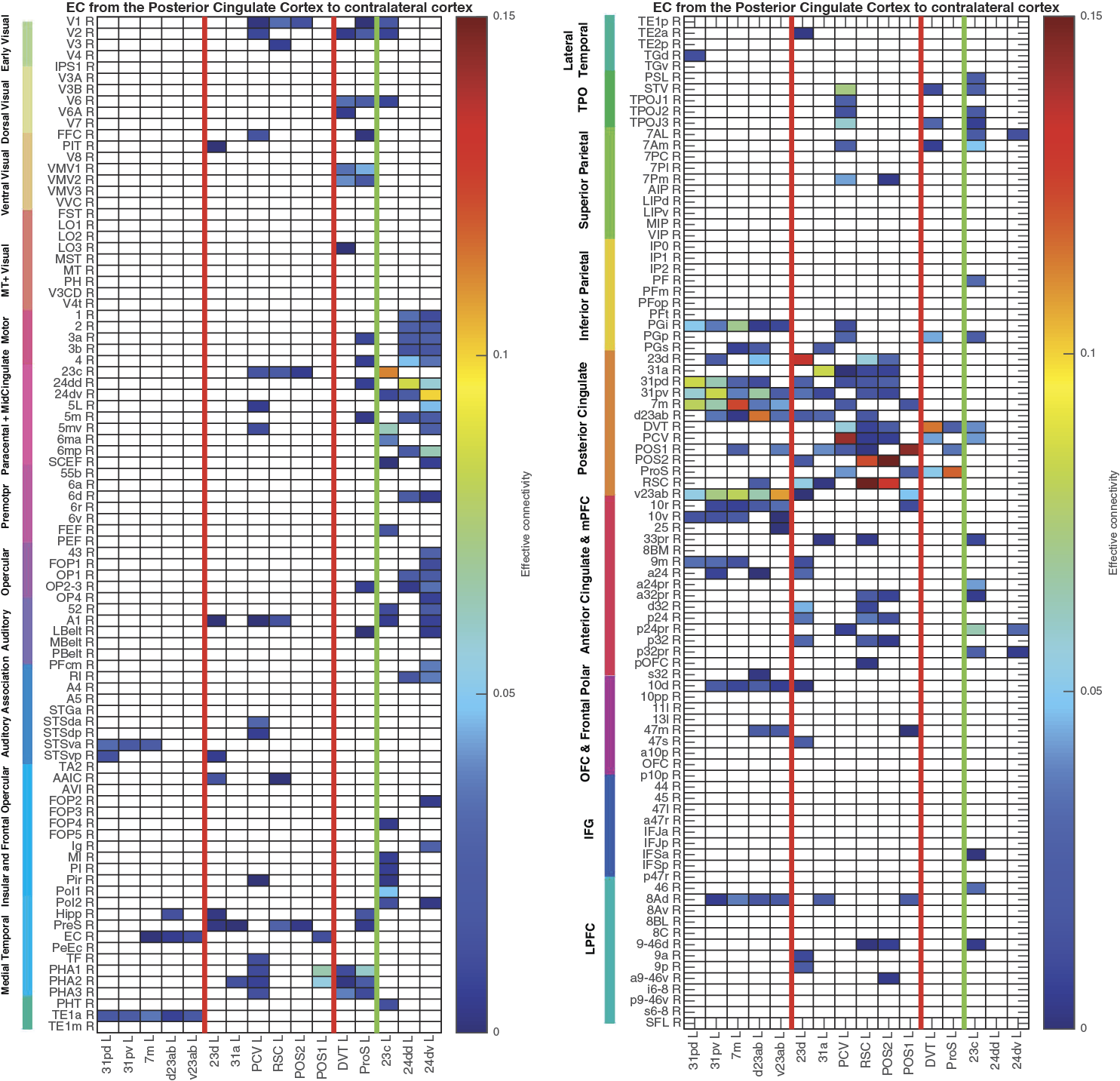


Fig. S3. Effective connectivity (EC) from the Left posterior cingulate division regions and mid-cingulate cortex to all 180 cortical areas in the Right hemisphere. All effective connectivities greater than 0 are shown, and effective connectivities of 0 are shown as a blank. The connectivities to the first set of cortical regions are shown on the left, and to the second set on the right. The three groups of posterior cingulate division regions are separated by red lines; and the mid-cingulate cortex effective connectivity is shown to the right of the green line. Abbreviations: see Table S1. The effective connectivity is read from column to row.


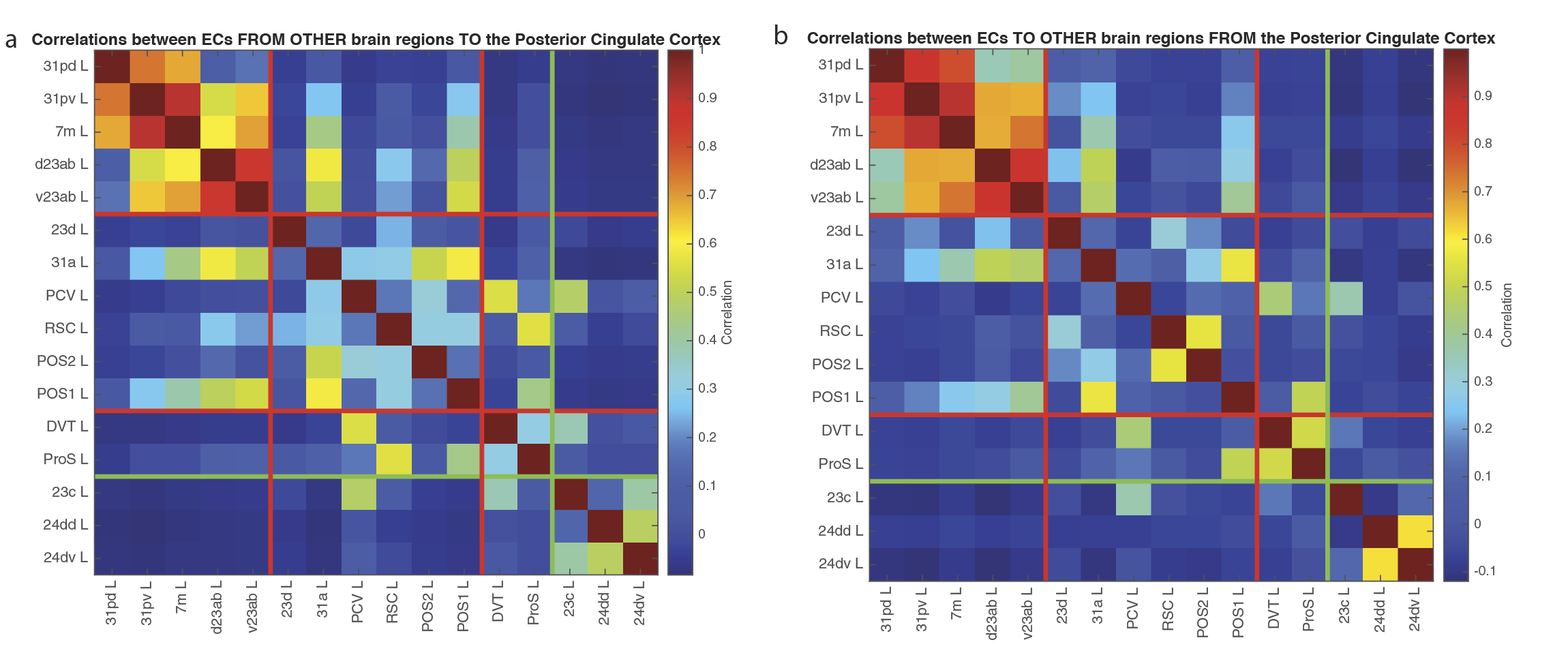


Fig. S4. a. Correlations between the effective connectivities from cortical areas to different regions in the posterior cingulate division regions and mid-cingulate cortex. b. Correlations between the effective connectivities to cortical areas from different regions in the posterior cingulate division and mid-cingulate cortex. Abbreviations: see Table S1. Based partly on these correlations and those for the functional connectivities in Fig. S5, three groups were identified in the PCC / RSC separated by red lines: Group 1 - 31pd, 31pv, 7m, d23ab and v23ab; Group2 - RSC, 31a, PCV, 23d, POS2, POS1; Group 3 – DVT, ProS. The mid-cingulate cortex identified by green lines is regions 23c, 24dd and 24dv.


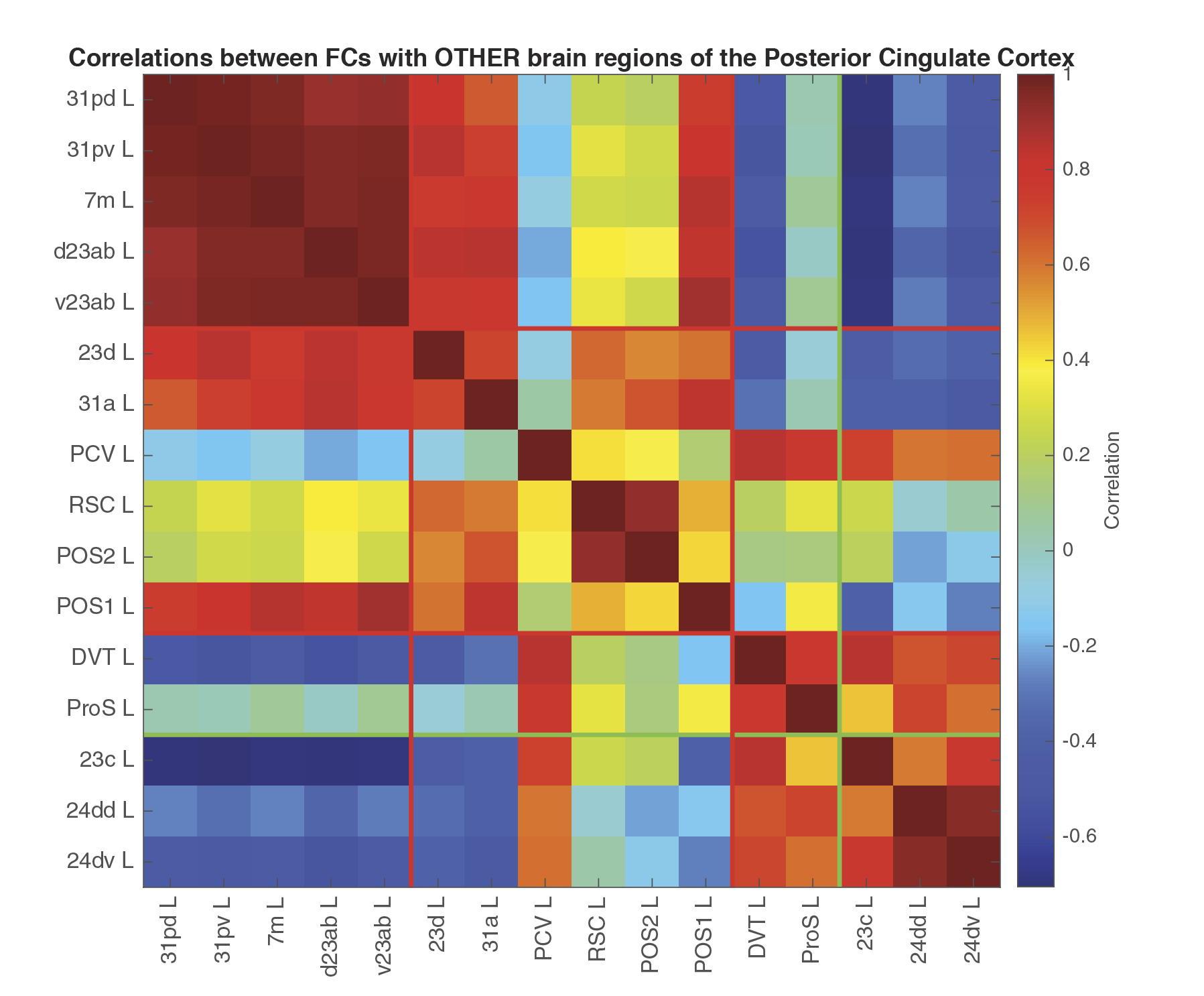


Fig. S5. Correlations between the functional connectivities with other cortical areas for the 16 different regions in the posterior cingulate division and the mid-cingulate cortex. Abbreviations: see Table S1.


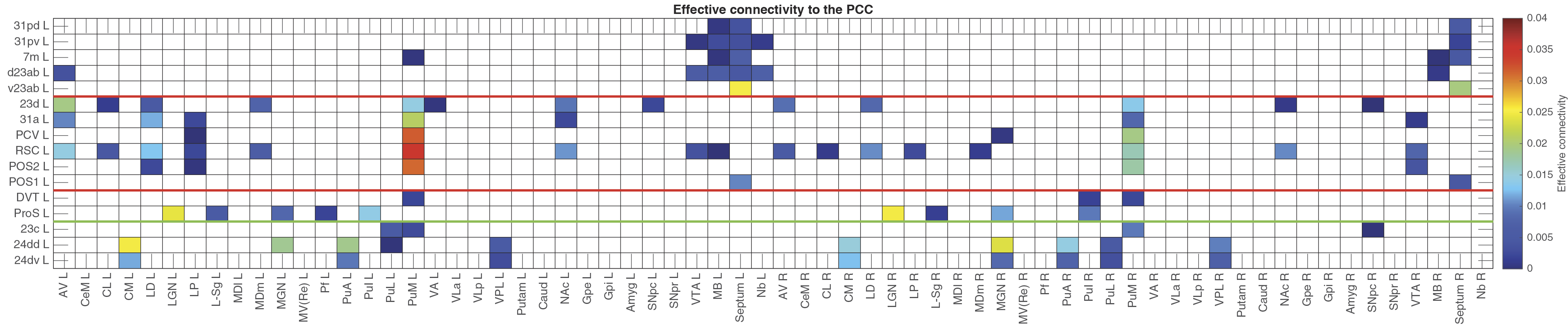


Fig. S6. Effective connectivity from subcortical regions to the posterior cingulate division regions and mid-cingulate cortex. The three groups of posterior cingulate division regions are separated by red lines; and the mid-cingulate cortex effective connectivity is shown below the green line. Abbreviations: see Table S1. The effective connectivity is read from column to row.


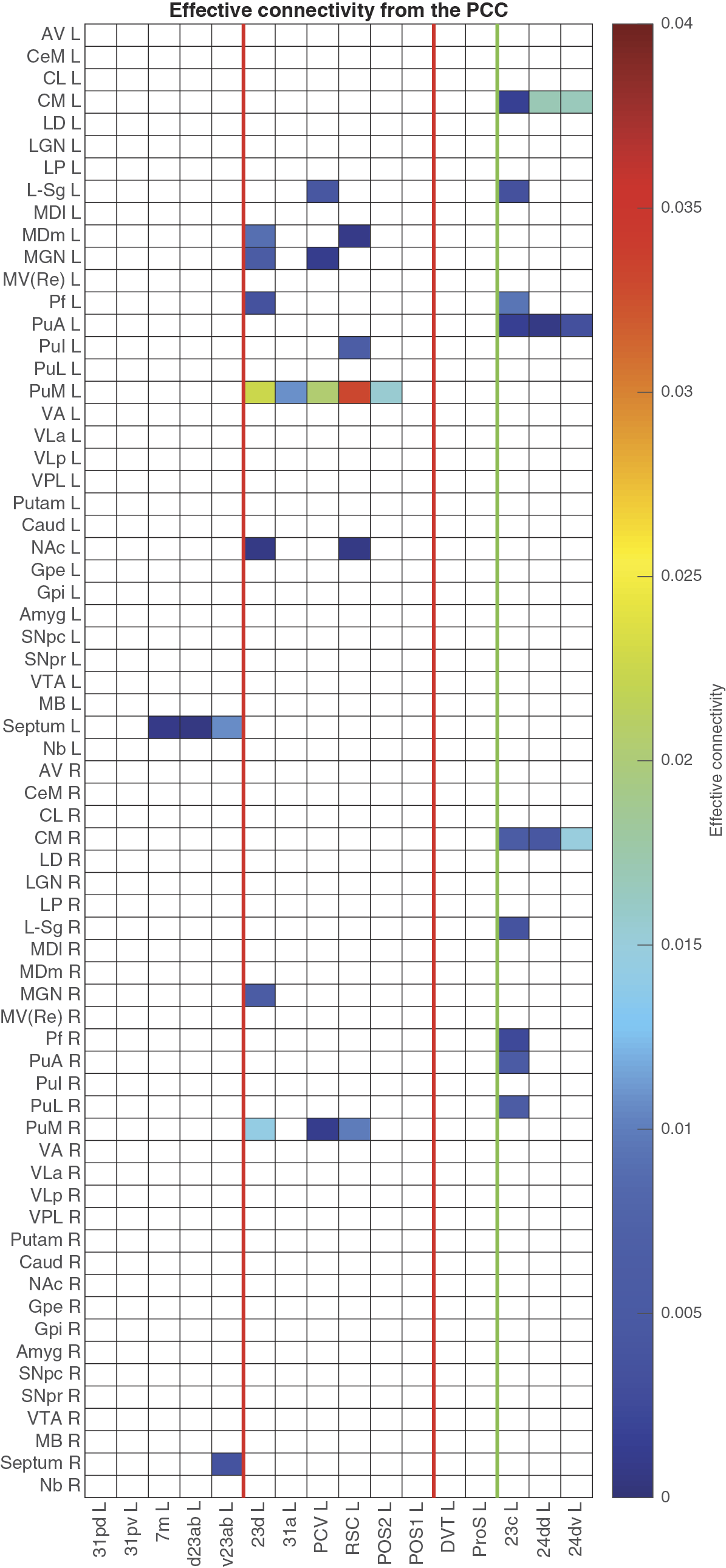


Fig. S7. Effective connectivity from the posterior cingulate division regions and mid-cingulate cortex to subcortical regions. The three groups of posterior cingulate cortex division regions are separated by red lines; and the mid-cingulate cortex effective connectivity is shown to the right of the green line. Abbreviations: see Table S1. The effective connectivity is read from column to row.


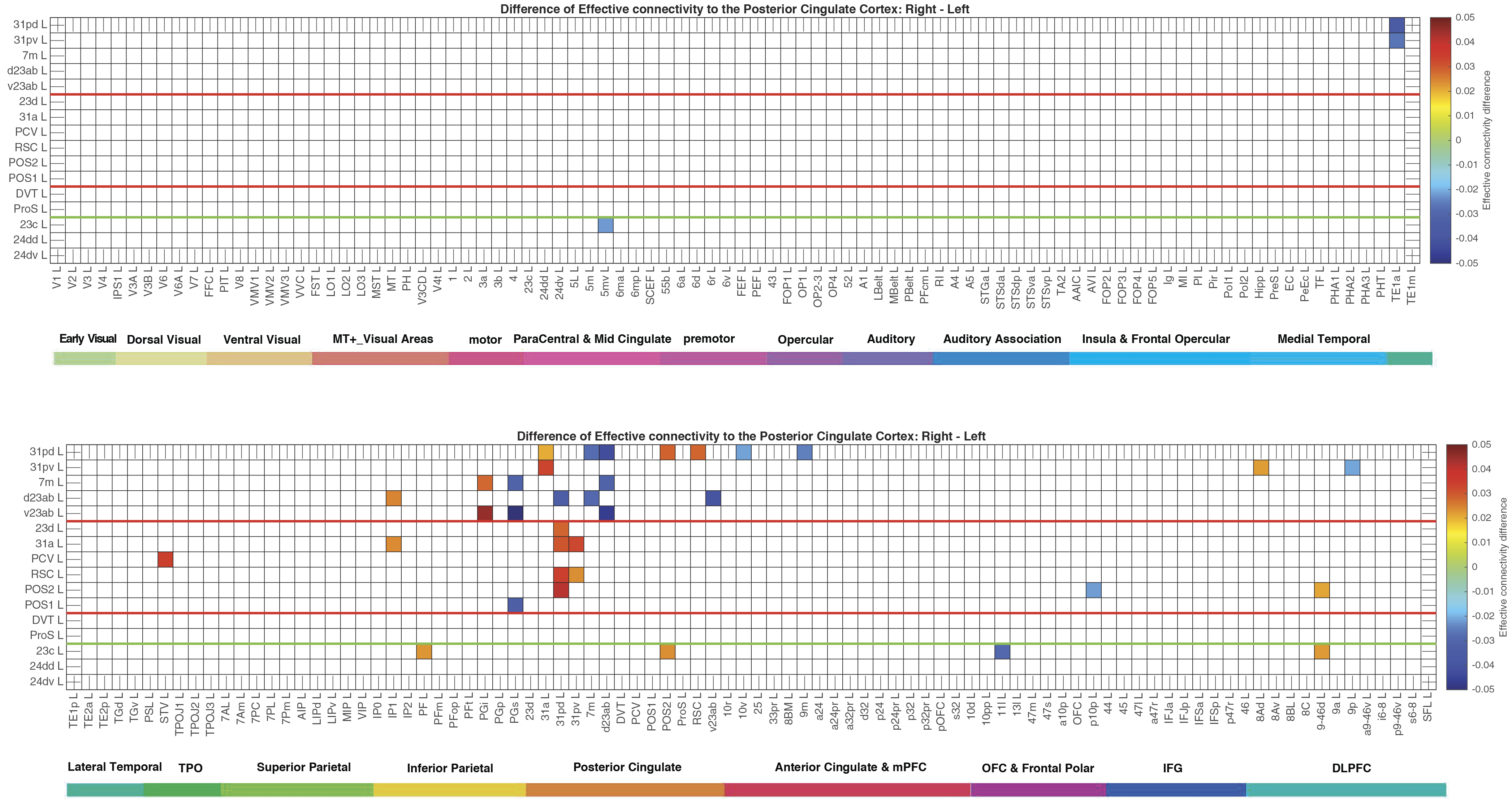


Fig. S8. Effective connectivity differences right hemisphere – left hemisphere *TO* the posterior cingulate cortex division regions (the rows) *FROM* 180 cortical areas (the columns) in the left hemisphere. The effective connectivity difference is read from column to row. Effective connectivity differences in the range -0.02 to 0.02 are shown as blank. Thus a red color in the matrix reflects a stronger effective connectivity for that link in the right hemisphere. Abbreviations: see Table S1. The three groups of posterior cingulate division regions are separated by red lines; and the mid-cingulate cortex functional connectivity is shown below the green line.


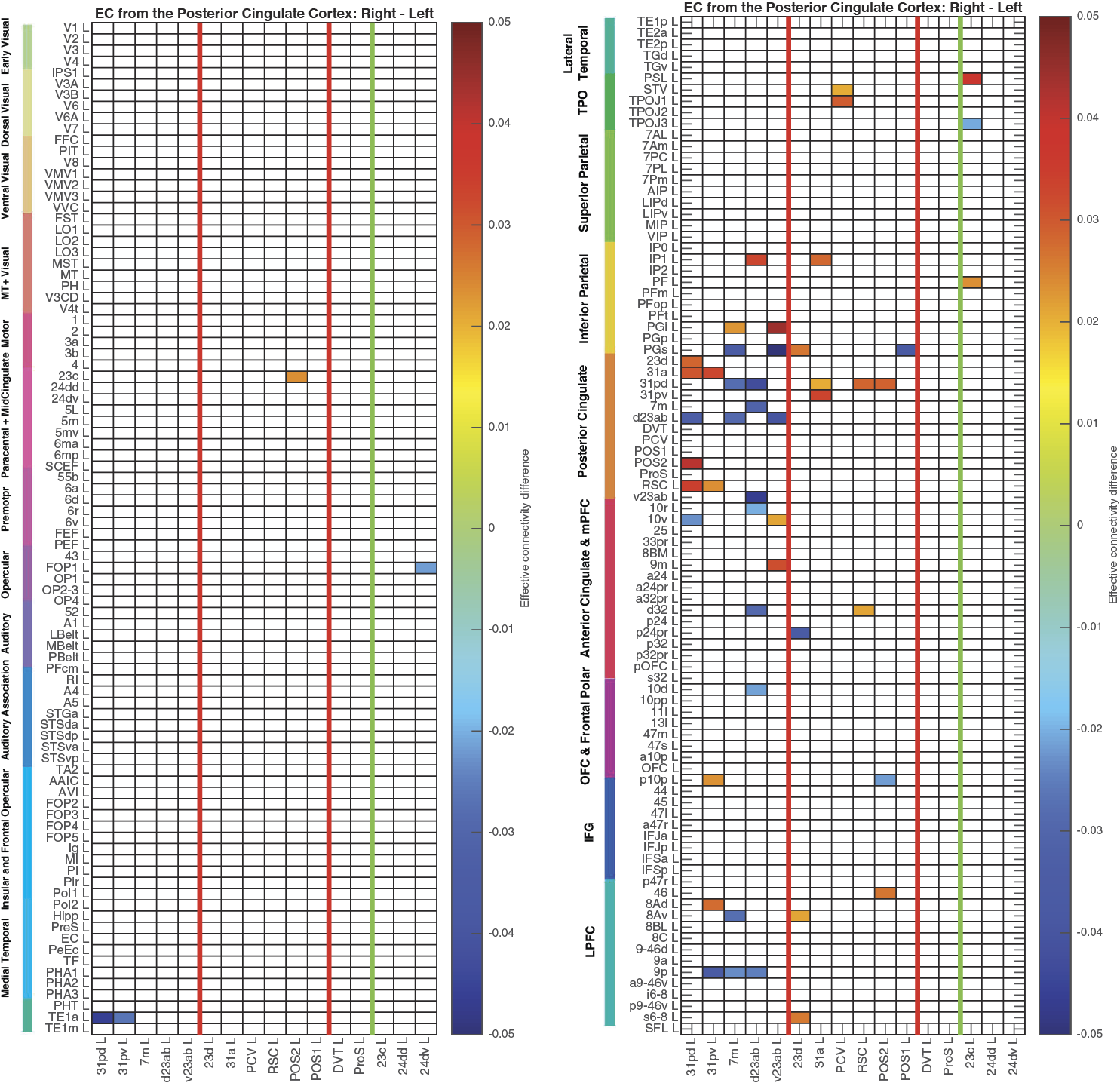


Fig. S9. **Effective connectivity difference right hemisphere – left hemisphere *FROM* the posterior cingulate division regions and mid-cingulate cortex *TO* 180 cortical areas in the left hemisphere.** The effective connectivity difference is read from column to row. Effective connectivity differences in the range -0.02 to 0.02 are shown as blank. Abbreviations: see Table S1. The three groups of posterior cingulate division regions are separated by red lines; and the mid-cingulate cortex functional connectivity is shown to the right of the green line.


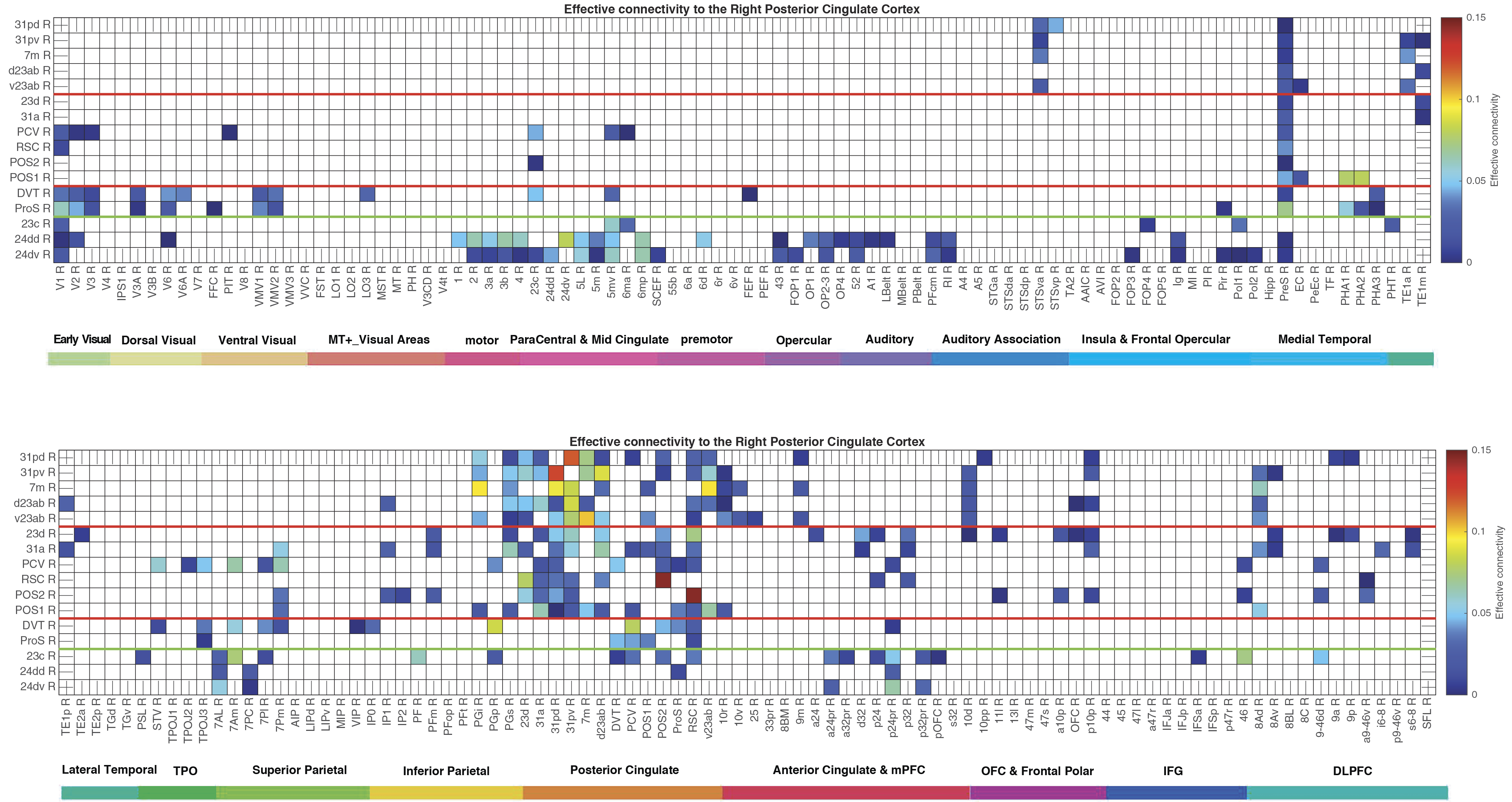


**Fig. S10. Effective connectivity *TO* the right posterior cingulate division regions (the rows) *FROM* 180 cortical areas (the columns) in the right hemisphere.** The effective connectivity is read from column to row. Effective connectivities of 0 are shown as blank. All effective connectivity maps are scaled to show 0.11 as the maximum, as this is the highest effective connectivity found between this set of brain regions. The effective connectivity algorithm for the whole brain is set to have a maximum of 0.2, and this was for connectivity between V1 and V2. Abbreviations: see Table S1. The three groups of posterior cingulate division regions are separated by red lines; and the mid-cingulate cortex effective connectivity is shown below the green line. The upper matrix is for the connectivity from the first 90 right hemisphere cortical regions listed in Table S1, and the lower matrix is for the next 90 regions listed in Table S1.


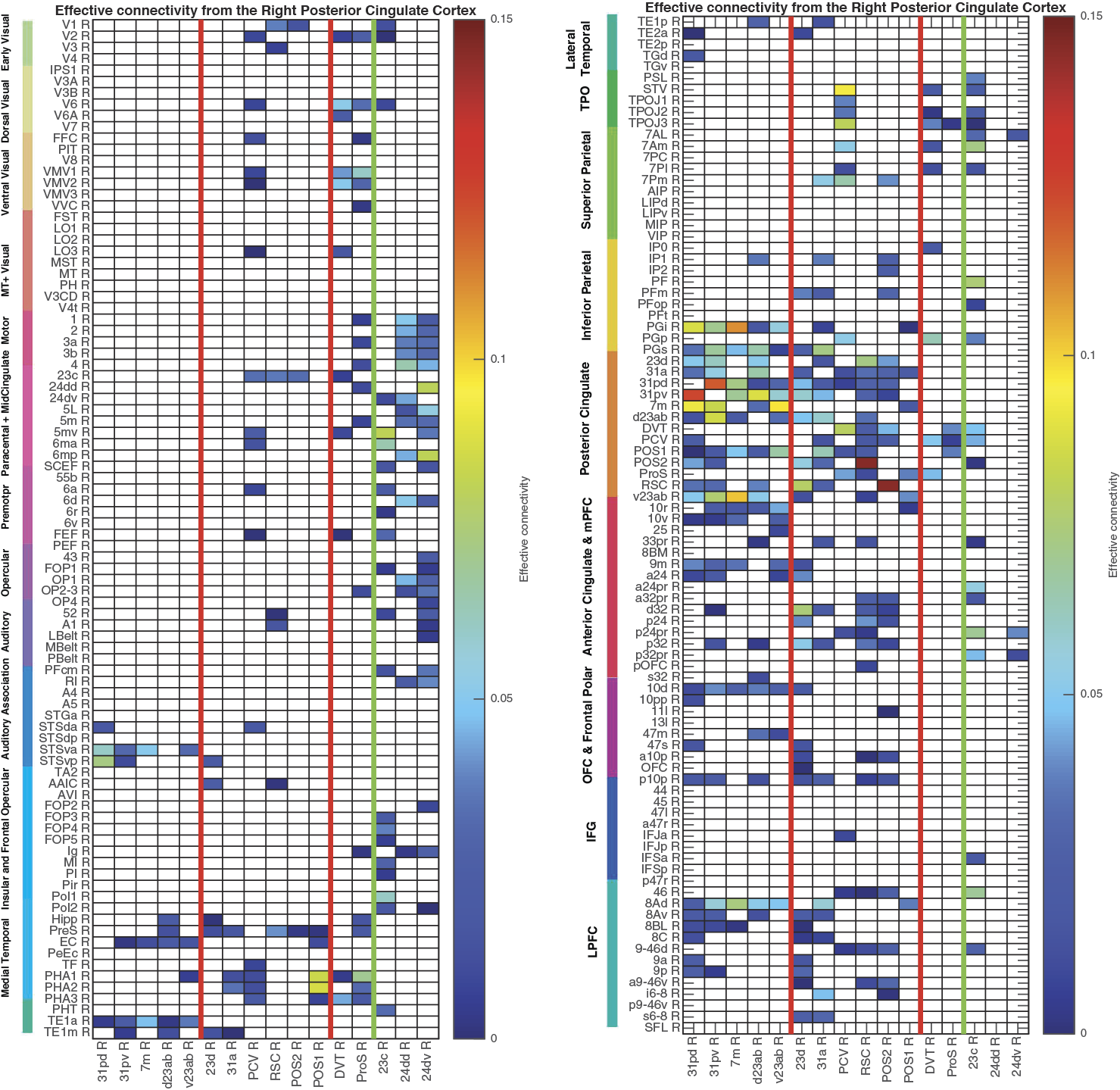


**Fig. S11. Effective connectivity *FROM* the right posterior cingulate division regions and mid-cingulate cortex *TO* 180 cortical areas in the right hemisphere.** The effective connectivity is read from column to row. Effective connectivities of 0 are shown as blank Abbreviations: see Table S1. The three groups of posterior cingulate division regions are separated by red lines; and the mid-cingulate cortex effective connectivity is shown to the right of the green line. The left matrix is for the connectivity to the first 90 right hemisphere cortical regions listed in Table S1, and the lower matrix is for the next 90 regions listed in Table S1.

References

Bajaj, S., Adhikari, B.M., Friston, K.J., Dhamala, M. (2016) Bridging the gap: Dynamic Causal Modeling and Granger Causality analysis of resting state functional magnetic resonance imaging. Brain Connect, 6:652-661.

Deco, G., Cabral, J., Woolrich, M.W., Stevner, A.B.A., van Hartevelt, T.J., Kringelbach, M.L. (2017a) Single or multiple frequency generators in on-going brain activity: A mechanistic whole-brain model of empirical MEG data. Neuroimage, 152:538-550.

Deco, G., Kringelbach, M.L., Jirsa, V.K., Ritter, P. (2017b) The dynamics of resting fluctuations in the brain: metastability and its dynamical cortical core. Sci. Rep., 7:3095.

Deco, G., Cruzat, J., Cabral, J., Tagliazucchi, E., Laufs, H., Logothetis, N.K., Kringelbach, M.L. (2019) Awakening: Predicting external stimulation to force transitions between different brain states. Proceedings of the National Academy of Sciences, 116:18088-18097.

Frassle, S., Lomakina, E.I., Razi, A., Friston, K.J., Buhmann, J.M., Stephan, K.E. (2017) Regression DCM for fMRI. Neuroimage, 155:406-421.

Freyer, F., Roberts, J.A., Becker, R., Robinson, P.A., Ritter, P., Breakspear, M. (2011) Biophysical mechanisms of multistability in resting-state cortical rhythms. J. Neurosci., 31:6353-61.

Freyer, F., Roberts, J.A., Ritter, P., Breakspear, M. (2012) A canonical model of multistability and scale-invariance in biological systems. PLoS Comput. Biol., 8:e1002634.

Friston, K. (2009) Causal modelling and brain connectivity in functional magnetic resonance imaging. PLoS Biol., 7:e33.

Friston, K.J., Kahan, J., Biswal, B., Razi, A. (2014) A DCM for resting state fMRI. Neuroimage, 94:396-407.

Gilson, M., Moreno-Bote, R., Ponce-Alvarez, A., Ritter, P., Deco, G. (2016) Estimation of directed effective connectivity from fMRI functional connectivity hints at asymmetries in the cortical connectome. PLoS Comput. Biol., 12:e1004762.

Glasser, M.F., Coalson, T.S., Robinson, E.C., Hacker, C.D., Harwell, J., Yacoub, E., Ugurbil, K., Andersson, J., Beckmann, C.F., Jenkinson, M., Smith, S.M., Van Essen, D.C. (2016) A multi-modal parcellation of human cerebral cortex. Nature, 536:171-8.

Huang, C.-C., Rolls, E.T., Hsu, C.-C.H., Feng, J., Lin, C.-P. (2021) Extensive cortical connectivity of the human hippocampal memory system: beyond the "what" and "where" dual-stream model. Cereb. Cortex, 31:4652-4669.

Huang, C.C., Rolls, E.T., Feng, J., Lin, C.P. (2022) An extended Human Connectome Project multimodal parcellation atlas of the human cortex and subcortical areas. Brain Struct Funct, 227:763-778.

Kringelbach, M.L., McIntosh, A.R., Ritter, P., Jirsa, V.K., Deco, G. (2015) The rediscovery of slowness: exploring the timing of cognition. Trends Cogn. Sci., 19:616-628.

Kringelbach, M.L., Deco, G. (2020) Brain states and transitions: insights from computational neuroscience. Cell Rep, 32:108128.

Kuznetsov, Y.A. (2013) Elements of applied bifurcation theory. New York. Springer Science & Business Media.

Razi, A., Seghier, M.L., Zhou, Y., McColgan, P., Zeidman, P., Park, H.J., Sporns, O., Rees, G., Friston, K.J. (2017) Large-scale DCMs for resting-state fMRI. Netw Neurosci, 1:222-241.

Rolls, E.T., Deco, G., Huang, C.C., Feng, J. (2022) The effective connectivity of the human hippocampal memory system. Cereb. Cortex, 32:3706-3725.

Valdes-Sosa, P.A., Roebroeck, A., Daunizeau, J., Friston, K. (2011) Effective connectivity: influence, causality and biophysical modeling. Neuroimage, 58:339-61.

Winterburn, J.L., Pruessner, J.C., Chavez, S., Schira, M.M., Lobaugh, N.J., Voineskos, A.N., Chakravarty, M.M. (2013) A novel in vivo atlas of human hippocampal subfields using high-resolution 3 T magnetic resonance imaging. Neuroimage, 74:254-65.
